# Supplementary material for: Postnatal maternal depressive symptoms and behavioural outcomes in term-born and preterm-born toddlers: a longitudinal UK community cohort study
Source: BMJ Open. 2022 Sep 1;12(9):e058540. doi: 10.1136/bmjopen-2021-058540 (PMC9438072; doi:10.1136/bmjopen-2021-058540)
Supplement: Supplementary data [file bmjopen-2021-058540supp001.pdf]

Supplemental material

Supplementary Table 1: CBCL internalising symptom model predictors using multiple imputation.

Supplementary Table 2: CBCL externalising symptom model predictors using multiple imputation.

Supplementary Table 3: CBCL and Q-CHAT model predictors using complete case analysis without interaction.

Supplementary Table 4: EPDS score predictors.

Supplementary Table 5: EPDS score predictors including interaction ‘term x time-lag’.

Supplementary Figure 1: Histogram showing the distribution of maternal EPDS scores at term-equivalent age.

Supplementary Table 1: CBCL internalising symptom model predictors using multiple imputation**.**

	**B [95%CI]**	**p**	**f^2**
**Maternal EPDS**	0.22 [0.08, 0.36]	.003 **	0.03
**Maternal BMI**	-0.04 [-0.13, 0.06]	.436	-
**Multiple pregnancy**	0.58 [-1.10, 2.27]	.497	-
**Parity**			
**1**	-0.37 [-1.41, 0.67]	.487	-
**2**	-1.33 [-3.09, 0.42]	.136	-
**3+**	0.64 [-1.34, 2.62]	.524	-
**IMD rank**	-0.41 [-0.91, 0.10]	.115	-
**Gestational age at birth (weeks)**	0.22 [-0.00, 0.44]	.053	-
**Birthweight (kg)**	-0.97 [-1.90, -0.05]	.038 *	0.005
**Sex: female**	-0.51 [-1.35, 0.33]	.232	-
**Corrected age at assessment (months)**	0.02 [-0.41, 0.45]	.923	-
**Cognition**	-0.05 [-0.10, -0.01]	.016 *	0.01

p<0.05 *; p<0.01 **; p<0.001 ***
Adjusted $R^2 = 0.0566$.
B = unstandardised coefficient.
Outcome variable = Child Behaviour Checklist internalising sub-score at 18 months. Maternal EPDS = maternal Edinburgh Postnatal Depression Scale score at term-equivalent age. Multiple pregnancy = dummy variable of twin/triplet pregnancy. Parity = dummy variable, one/two/three+ previous child(ren). Corrected age at assessment (months) = age at behavioural assessment, corrected for gestational age. Cognition = infant Bayley III score at 18 months.
Effect size (Cohen's f^2, calculated from squared part correlations for predictors significant to 0.05): 0.02 = small, 0.15 = medium and 0.35 = large.[1]
- indicates data not given, as predictor not significant to 0.05.

Supplementary Table 2: CBCL externalising symptom model predictors using multiple imputation.

	B [95%CI]	p	f^2
**Maternal EPDS**	0.40 [0.20, 0.61]	<.001 ***	0.05
**Maternal BMI**	0.01 [-0.17, 0.15]	.933	-
**Multiple pregnancy**	2.51 [-0.29, 5.31]	.079	-
**Parity**			
**1**	-1.06 [-2.53, 0.42]	.160	-
**2**	-0.61 [-3.55, 2.33]	.682	-
**3+**	-0.96 [-4.47, 2.56]	.593	-
**IMD rank**	-0.24 [-1.11, 0.63]	.585	-
**Gestational age at birth (weeks)**	-0.07 [-0.40, 0.27]	.701	-
**Birthweight (kg)**	1.03 [-0.38, 2.44]	.153	-
**Sex: female**	-1.80 [-3.07, -0.53]	.006 **	0.06
**Corrected age at assessment (months)**	-0.40 [-0.95, 0.16]	.161	-
**Cognition**	0.03 [-0.03, 0.10]	.322	-

p<0.05 *; p<0.01 **; p<0.001 ***
Adjusted R^2 = 0.0612.
B = unstandardised coefficient.
Outcome variable = Child Behaviour Checklist externalising sub-score at 18 months. Maternal EPDS = maternal Edinburgh Postnatal Depression Scale score at term-equivalent age. Multiple pregnancy = dummy variable of twin/triplet pregnancy. Parity = dummy variable, one/two/three+ previous child(ren). Corrected age at assessment (months) = age at behavioural assessment, corrected for gestational age.
Cognition = infant Bayley III score at 18 months.
Effect size (Cohen's f^2, calculated from squared part correlations for predictors significant to 0.05): 0.02 = small, 0.15 = medium and 0.35 = large.[1]
- indicates data not given, as predictor not significant to 0.05.

Supplementary Table 3: CBCL and Q-CHAT model predictors using complete case analysis without interaction.

	**CBCL**		**Q-CHAT**	
	**B [95%CI]**	**p**	**B [95%CI]**	**p**
**Maternal EPDS**	0.88 [0.35, 1.41]	.001 **	0.21 [-0.02, 0.44]	.069
**Maternal BMI**	-0.01 [-0.38, 0.37]	.963	0.01 [-0.16, 0.17]	.930
**Multiple pregnancy**	1.50 [-6.87, 9.87]	.724	0.43 [-2.50, 3.37]	.772
**Parity**				
**1**	-2.83 [-6.60, 0.94]	.141	-1.54 [-3.41, 0.34]	.108
**2**	-3.49 [-10.3, 3.35]	.316	0.13 [-2.85, 3.10]	.933
**3+**	-1.38 [-10.0, 7.30]	.755	-1.43 [-4.50, 1.64]	.360
**IMD rank**	-1.44 [-3.56, 0.67]	.181	-1.75 [-2.70, -0.79]	<.001 ***
**Gestational age at birth (weeks)**	0.01 [-0.90, 0.91]	.987	0.10 [-0.33, 0.54]	.639
**Birthweight (kg)**	-0.65 [-4.38, 3.08]	.733	-1.81 [-3.63, 0.00]	.050
**Sex: female**	-4.57 [-7.82, -1.31]	.006 **	-2.12 [-3.60, -0.64]	.005 **
**Corrected age at assessment (months)**	-0.84 [-2.26, 0.59]	.247	-0.41 [-1.18, 0.35]	.290
**Cognition**	-0.03 [-0.20, 0.13]	.689	-0.23 [-0.30, -0.15]	<.001 ***

p<0.05 *; p<0.01 **; p<0.001 ***
CBCL adjusted $R^2 = 0.0862$. Q-CHAT adjusted $R^2 = 0.2103$.
B = unstandardised coefficient.
CBCL = Child Behaviour Checklist externalising sub-score at 18 months. Q-CHAT = Quantitative Checklist for Autism in Toddlers score at 18 months. Maternal EPDS = maternal Edinburgh Postnatal Depression Scale score at term-equivalent age. Multiple pregnancy = dummy variable of twin/triplet pregnancy. Parity = dummy variable, one/two/three+ previous child(ren). Gestation (weeks) = dummy variable: 34-36+6 weeks and ≥37 weeks gestation at birth. Corrected age at assessment (months) = age at behavioural assessment, corrected for gestational age. Cognition = infant Bayley III score at 18 months.

Supplementary Table 4: EPDS score predictors.

	**IRR [95%CI]**	**p**
**Time-lag (weeks)**	1.01 [0.97, 1.05]	.647
**Gestation:term**	0.91 [0.64, 1.31]	.627
**IMD rank**	1.00 [1.00, 1.00]	.103
**Multiple pregnancy**	0.66 [0.46, 0.96]	.031 *
**Parity**		
**1**	0.79 [0.66, 0.95]	.011 *
**2**	0.87 [0.60, 1.28]	.491
**3+**	0.84 [0.54, 1.31]	.445
**Birthweight (kg)**	0.98 [0.83, 1.17]	.847
**Sex:female**	1.13 [0.98, 1.31]	.098

$p<0.05$ *; $p<0.01$ **; $p<0.001$ ***

Pseudo $R^2 = 0.0228$

IRR = incidence rate ratio

Outcome variable = maternal Edinburgh Postnatal Depression Scale (EPDS) score at term-equivalent age. Time-lag (weeks) = time in weeks between birth and EPDS assessment. Gestation:term = dummy variable, term (≥37 weeks) vs preterm (<37 weeks) gestation at birth. Multiple pregnancy = dummy variable of twin/triplet pregnancy. Parity = dummy variable, one/two/three+ previous child(ren).

Supplementary Table 5: EPDS score predictors including interaction 'term x time-lag'.

	**IRR [95%CI]**	**p**
**Time-lag (weeks)**	1.00 [0.97, 1.04]	.823
**Gestation: term**	0.88 [0.58, 1.34]	.553
**IMD rank**	1.00 [1.00, 1.00]	.104
**Multiple pregnancy**	0.66 [0.46, 0.96]	.029 *
**Parity**		
**1**	0.79 [0.66, 0.95]	.010 *
**2**	0.87 [0.60, 1.28]	.480
**3+**	0.85 [0.55, 1.31]	.458
**Birthweight (kg)**	0.97 [0.83, 1.15]	.756
**Sex:female**	1.13 [0.98, 1.30]	.100
**Term x time-lag (weeks)**	1.01 [0.94, 1.10]	.735

$p<0.05$ *; $p<0.01$ **; $p<0.001$ ***
Pseudo $R^2 = 0.0230$
IRR = incidence rate ratio
Outcome variable = maternal Edinburgh Postnatal Depression Scale (EPDS) score at term-equivalent age. Time-lag (weeks) = time in weeks between birth and EPDS assessment. Gestation: term = dummy variable, term (≥37 weeks) vs preterm (<37 weeks) gestation at birth. Multiple pregnancy = dummy variable of twin/triplet pregnancy. Parity = dummy variable, one/two/three+ previous child(ren). Term x time-lag (weeks): interaction term between term gestation at birth and time-lag between birth and maternal EPDS assessment.

Supplementary Figure 1: Histogram showing the distribution of maternal EPDS scores at term-equivalent age.

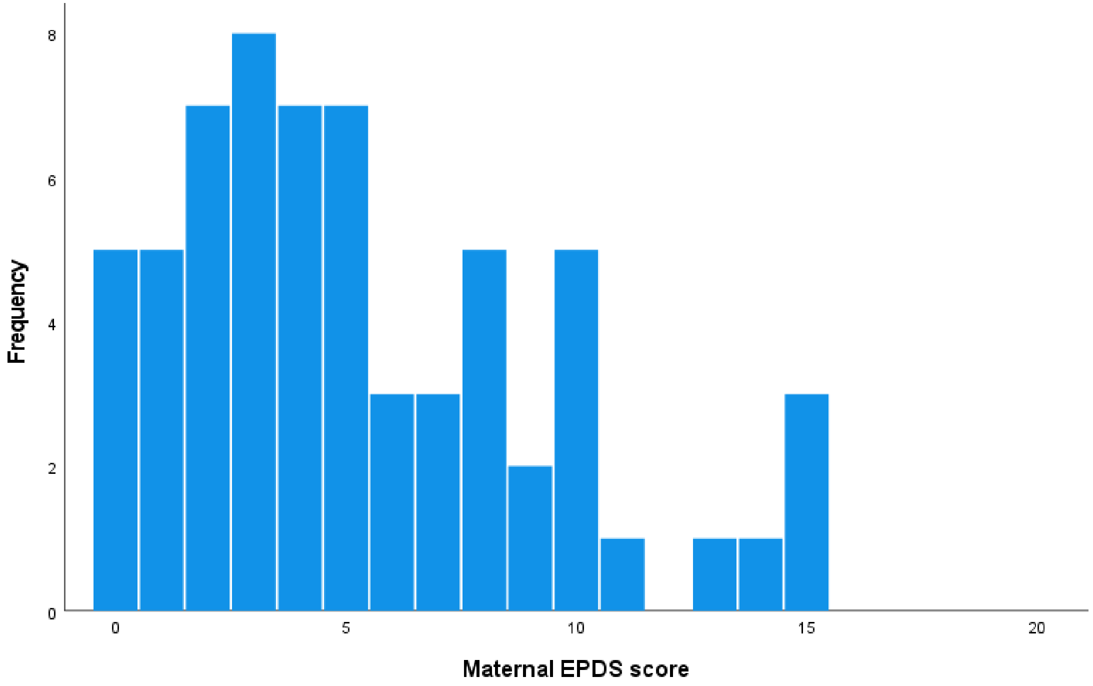

Supplemental reference list

1. Cohen J. *Statistical Power Analysis for the Behavioral Sciences*. 2nd ed. L. Erlbaum Associates; 1988.
