# Supplementary material for: Postnatal maternal depressive symptoms and behavioural outcomes in term-born and preterm-born toddlers: a longitudinal UK community cohort study
Source: BMJ Open. 2022 Sep 1;12(9):e058540. doi: 10.1136/bmjopen-2021-058540 (PMC9438072; doi:10.1136/bmjopen-2021-058540)
Supplement: Author's manuscript [file bmjopen-2021-058540.draft_revisions.pdf]

BMJ Open is committed to open peer review. As part of this commitment we make the peer review history of every article we publish publicly available.

When an article is published we post the peer reviewers' comments and the authors' responses online. We also post the versions of the paper that were used during peer review. These are the versions that the peer review comments apply to.

The versions of the paper that follow are the versions that were submitted during the peer review process. They are not the versions of record or the final published versions. They should not be cited or distributed as the published version of this manuscript.

BMJ Open is an open access journal and the full, final, typeset and author-corrected version of record of the manuscript is available on our site with no access controls, subscription charges or pay-per-view fees (http://bmjopen.bmj.com).

If you have any questions on BMJ Open's open peer review process please email info.bmjopen@bmj.com

BMJ Open

Postnatal maternal depressive symptoms and behavioural outcomes in term- and preterm-born toddlers

Journal:	*BMJ Open*
Manuscript ID	bmjopen-2021-058540
Article Type:	Original research
Date Submitted by the Author:	20-Oct-2021
Complete List of Authors:	Kleine, Ira; King's College London, Centre for the Developing Brain, School of Bioengineering and Imaging Sciences, Faculty of Life Sciences & Medicine<br>Vamvakas, George; King's College London, Department of Biostatistics and Health Informatics, Institute of Psychiatry, Psychology and Neuroscience<br>Lautarescu, Alexandra; King's College London, Centre for the Developing Brain, School of Bioengineering and Imaging Sciences, Faculty of Life Sciences & Medicine; King's College London, Forensic and Neurodevelopmental Sciences, Institute of Psychiatry, Psychology and Neuroscience<br>Falconer, Shona; King's College London, Centre for the Developing Brain, School of Bioengineering and Imaging Sciences, Faculty of Life Sciences & Medicine<br>Chew, Andrew; King's College London, Centre for the Developing Brain, School of Bioengineering and Imaging Sciences, Faculty of Life Sciences & Medicine<br>Counsell, Serena ; King's College London, Centre for the Developing Brain, School of Bioengineering and Imaging Sciences, Faculty of Life Sciences & Medicine<br>Pickles, Andrew; King's College London, Department of Biostatistics and Health Informatics, Institute of Psychiatry, Psychology and Neuroscience<br>Edwards, David; King's College London, Centre for the Developing Brain, School of Bioengineering and Imaging Sciences, Faculty of Life Sciences & Medicine<br>Nosarti, Chiara; King's College London, Centre for the Developing Brain, School of Bioengineering and Imaging Sciences, Faculty of Life Sciences & Medicine; King's College London, Department of Child and Adolescent Psychiatry, Institute of Psychiatry, Psychology and Neuroscience
Keywords:	MENTAL HEALTH, Child & adolescent psychiatry < PSYCHIATRY, Developmental neurology & neurodisability < PAEDIATRICS, Depression & mood disorders < PSYCHIATRY

SCHOLARONE™
Manuscripts

I, the Submitting Author has the right to grant and does grant on behalf of all authors of the Work (as defined in the below author licence), an exclusive licence and/or a non-exclusive licence for contributions from authors who are: i) UK Crown employees; ii) where BMJ has agreed a CC-BY licence shall apply, and/or iii) in accordance with the terms applicable for US Federal Government officers or employees acting as part of their official duties; on a worldwide, perpetual, irrevocable, royalty-free basis to BMJ Publishing Group Ltd ("BMJ") its licensees and where the relevant Journal is co-owned by BMJ to the co-owners of the Journal, to publish the Work in this journal and any other BMJ products and to exploit all rights, as set out in our licence.

The Submitting Author accepts and understands that any supply made under these terms is made by BMJ to the Submitting Author unless you are acting as an employee on behalf of your employer or a postgraduate student of an affiliated institution which is paying any applicable article publishing charge ("APC") for Open Access articles. Where the Submitting Author wishes to make the Work available on an Open Access basis (and intends to pay the relevant APC), the terms of reuse of such Open Access shall be governed by a Creative Commons licence – details of these licences and which Creative Commons licence will apply to this Work are set out in our licence referred to above.

Other than as permitted in any relevant BMJ Author's Self Archiving Policies, I confirm this Work has not been accepted for publication elsewhere, is not being considered for publication elsewhere and does not duplicate material already published. I confirm all authors consent to publication of this Work and authorise the granting of this licence.

For peer review only

Postnatal maternal depressive symptoms and behavioural outcomes in term- and preterm-born toddlers

Kleine, I[a] (MBBS); Vamvakas, G[b] (MSc); Lautarescu A[a,c] (MPhil); Falconer, S[a] (PhD); Chew A[a] (MD); Counsell SJ[a] (PhD); Pickles, A[b] (FMedSci); Edwards, AD[a] (FMedSci); Nosarti, C[a,d] (PhD).

[a] Centre for the Developing Brain, School of Bioengineering and Imaging Sciences, Faculty of Life Sciences & Medicine, King's College London, St Thomas' Hospital, London SE1 7EH, UK.

[b] Department of Biostatistics and Health Informatics, Institute of Psychiatry, Psychology and Neuroscience, King's College London, London SE5 8AF, UK.

[c] Forensic and Neurodevelopmental Sciences, Institute of Psychiatry, Psychology and Neuroscience, King's College London, London SE5 8AF, UK.

[d] Department of Child and Adolescent Psychiatry, Institute of Psychiatry, Psychology and Neuroscience, King's College London, London SE5 8AF, UK.

Post-publication corresponding author's contact information

Prof Chiara Nosarti

Centre for the Developing Brain, School of Bioengineering and Imaging Sciences

King's College London

St Thomas' Hospital

London

SE1 7EH

UK

chiara.nosarti@kcl.ac.uk

+44 (0)207 848 0133

For peer review only - http://bmjopen.bmj.com/site/about/guidelines.xhtml

Abstract

Objectives: To examine the association between maternal depressive symptoms in the immediate postnatal period and offspring's mental health in a large cohort of term- and preterm-born toddlers.

Design and Participants: Data were drawn from the Developing Human Connectome Project. Maternal postnatal depressive symptoms were assessed at term, and children's outcomes were evaluated at a median corrected age of 18.4 months (range 17.3 – 24.3).

Exposure and outcomes: Preterm birth was defined as <37 weeks completed gestation. Maternal depressive symptoms were assessed with the Edinburgh Postnatal Depression Scale (EPDS). Toddlers' outcome measures were parent-rated Child Behaviour Checklist $1^{1/2}$-5 Total (CBCL) and Quantitative Checklist for Autism in Toddlers (Q-CHAT) scores. Toddlers' cognition was assessed with the Bayley Scales of Infant and Toddler Development – Third Edition (Bayley-III).

Results: Higher maternal EPDS scores were associated with toddlers' higher CBCL (B=0.93, 95% CI 0.43-1.44, p<0.001, f^2=0.05) and Q-CHAT scores (B=0.27, 95% CI 0.03-0.52, p=.031, f^2=0.01). Higher maternal EPDS scores were not associated with toddlers' cognitive outcomes. Maternal EPDS, toddlers' CBCL and Q-CHAT scores did not differ between preterm (n=97; 19.1% of the total sample) and term participants.

Conclusions: Our findings indicate that children whose mothers had increased depressive symptoms in the early postnatal period, including subclinical symptoms, exhibit more maternally-reported behavioural problems in toddlerhood. These associations were independent of gestational age. Further research is needed to confirm the clinical significance of these findings.

For peer review only

Strengths and limitations of this study

- Prospective study with a large sample, using multiple imputation to reduce non-response bias.
- Maternal depressive symptoms assessed as a continuous variable, providing more nuanced information about the significance of subclinical symptoms.
- Maternal depressive symptoms assessed earlier than in previous studies, enabling recognition of early screening opportunities for families.
- Potential shared method variance bias through parent-completed child behavioural assessments.
- Unknown paternal and parental factors, such as comorbid psychiatric conditions, that may confound our findings.

Keywords

Postpartum Depression; Child Development; Autism; Mental Health; Child Behavior; Mood Disorder.

Declarations

Funding

The DHCP project was funded by the European Research Council under the European Union Seventh Framework Programme (FR/2007-2013)/ERC Grant Agreement no. 319456. The authors acknowledge infrastructure support from the National Institute for Health Research Mental Health Biomedical Research Centre at South London, Maudsley NHS Foundation Trust, King's College London, the National Institute for Health Research Mental Health Biomedical Research Centre at Guys, and St Thomas' Hospitals NHS Foundation Trust. The study was also supported in part by the Engineering and Physical Sciences Research Council / Wellcome Trust Centre for Medical Engineering at King's College London (grant WT 203148/Z/16/Z) and the Medical Research Council (UK) (grants MR/K006355/1 and MR/L011530/1) and the MRC Centre for Neurodevelopmental Disorders at King's College London. AP receives a NIHR SI award (NF-SI-0617-10120). AL is supported by the UK Medical Research Council (MR/N013700) and King's College London member of the MRC Doctoral Training Partnership in Biomedical Sciences. The views expressed are those of the authors and not necessarily those of the NHS, the NIHR, or the Department of Health and Social Care.

For peer review only - http://bmjopen.bmj.com/site/about/guidelines.xhtml

Conflict of interest / Competing interests

ADE received financial support from the EU-AIMS-Trials (European Research Council under the European Union Seventh Framework Programme) as co-Principal Investigator. ADE received consulting fees from Chiesi Farmaceutici (advice on neuroprotection in newborn infants) and Medtronix (unpaid participation in scientific advice committee). ADE has a patent on Xenon as an organ protectant (No P023708WO). ADE was Chair of the Data Monitoring and Ethics Committee for the Baby-Oscar Trial, and served on the Data Monitoring and Ethics Committee for the PAEN Trial.

There are no other relationships or activities that could appear to have influenced the submitted work.

Availability of data and material

Research data will be available as part of Developing Human Connectome Project (http://www.developingconnectome.org/).

Code availability

Not applicable.

Ethics approval

This study was approved by the UK National Research Ethics Authority (14/LO/1169) and conducted in accordance with the World Medical Association's Code of Ethics (Declaration of Helsinki).

Consent to participate

Written informed consent was given by children's carer(s) at recruitment into the study.

Consent for publication

Not applicable.

Patient and Public Involvement statement

The current study was developed in consultation with the Weston Programme for Family Centered Research, which involves parents to define what research is valuable to them, and to allow them to lead it with support from the scientists in the Centre for the Developing Brain.

For peer review only - http://bmjopen.bmj.com/site/about/guidelines.xhtml

Introduction

Postnatal depression affects approximately 12% of mothers worldwide.[1] In contrast to 'baby blues', which is a state of emotional lability that affects between 30-80% of women in the first few days after birth and typically resolves spontaneously within two weeks,[2] postnatal depression is more severe and starts in the first few months post-partum[1]. Stressful life events have been linked to a heightened risk of developing postnatal depression,[3] with rates as high as 40% in women who give birth before term completion (i.e., preterm, < 37 gestational weeks),[4] likely due to heightened stress associated with perinatal complications.[5]

Women with postnatal depression tend to be less responsive to their baby's needs and to display less affection.[6] Therefore, in the short-term postpartum depression may affect mother-infant interactions[7] and in the long-term it may lead to alterations in brain development,[8] emotional difficulties,[9] less secure attachment, cognitive and behavioural problems in childhood, and a possible increased risk of autism spectrum disorder (ASD).[10,11] Large cohort studies, such as the Avon Longitudinal Study of Parents and Children (ALSPAC), have shown that these associations are even evident when maternal depression is measured on a continuum of symptoms rather than a dichotomous diagnosis,[12–14] supporting the notion that elevated sub-diagnostic psychiatric symptoms can also negatively impact on children's development.[15]

Studies investigating the underlying causes that may link maternal postnatal depression to child outcomes have implicated several biological and environmental variables. For instance, genetic and epigenetic factors have been shown to both mediate and mitigate the intergenerational transmission of psychiatric disorders,[16] while lower quality parenting, interparental conflict, and socioeconomic deprivation have been shown to exacerbate children's developmental risk.[11] In addition, preterm birth has been associated with alterations in early brain development,[17] as well as neurological, behavioural and cognitive problems in childhood and beyond.[18,19] Therefore, it is complex to disentangle the possible effects of postnatal maternal mental health and those of perinatal clinical factors on specific outcomes in preterm children, as these may involve both maternal psychosocial and biological variables and child preterm-related neurodevelopmental morbidity. Furthermore, a question that remains unanswered is whether perinatal clinical risk accentuates the association between maternal postnatal depressive symptoms and child outcome. Previous research has proposed a diathesis-stress model, whereby preterm birth is regarded as a

For peer review only - http://bmjopen.bmj.com/site/about/guidelines.xhtml

vulnerability factor that makes preterm infants more prone to suboptimal environmental influences compared to term infants.[20,21] On the other hand, the differential susceptibility model frames preterm birth as a plasticity factor that makes infants more likely to have both poorer outcomes in negative environments, as well as better outcomes in supportive environments.[21,22]

Given that mothers of preterm children experience elevated levels of distress,[23] are at high risk of developing postnatal depression,[4] and that preterm children themselves are vulnerable to psychiatric sequelae,[24] we aimed to investigate the association between very early symptoms of maternal postnatal depression and child behavioural and emotional outcomes, as well as whether this association was influenced by gestational age. We hypothesise that early postnatal maternal depressive symptoms would be more elevated in mothers of preterm compared to term infants and that these would impact preterm children's behavioural and emotional outcomes to a greater degree than their term counterparts.

Methods

Sample

Participants were enrolled in the Developing Human Connectome Project (DHCP, http://www.developingconnectome.org/). Toddlers were invited to the Centre for the Developing Brain, St Thomas' Hospital, London, for neurodevelopmental assessment between 17 and 24 months post-expected delivery date. Inclusion criteria for our follow-up study were: mother and baby attendance for magnetic resonance imaging (MRI) at term corrected age; completed toddler neurodevelopmental assessment. 509 toddlers met these inclusion criteria by the date of closure for this analysis (26/02/2020). This study was approved by the UK National Research Ethics Authority (14/LO/1169) and conducted in accordance with the World Medical Association's Code of Ethics (Declaration of Helsinki). Written informed consent was given by children's carer(s) at recruitment into the study.

Maternal variables

Maternal age, parity, Body Mass Index (BMI) and postcode were collected at enrolment into the DHCP study. Parity was coded as 0, 1, 2, or ≥3 previous children. Index of Multiple Deprivation (IMD) rank was computed from the current maternal postcode using the 2019 IMD classification,[25] and provided a proxy for family socioeconomic status. Lower IMD rank corresponds to greater social deprivation.

For peer review only - http://bmjopen.bmj.com/site/about/guidelines.xhtml

Maternal depressive symptoms were measured using the Edinburgh Postnatal Depression Scale (EPDS)[26] at term corrected age. The EPDS is a 10-item screening questionnaire completed by mothers, with higher scores reflecting a higher likelihood of depressive disorders. A score of 13 can be used as a cut-off indicating high-level symptoms, although a cut-off of 11 maximises the sensitivity and specificity of the screening tool for depression.[27]

<u>Child variables</u>

Infant *clinical characteristics* included: sex, gestational age at birth, birth weight, and pregnancy size (singleton/twin/triplet).

Behavioural outcomes were assessed using the Child Behaviour Checklist/$1^{1/2}$-5 (CBCL), a parent-completed 100-item questionnaire, in which the parent rates the child's behaviour over the preceding two months using a 3-point Likert scale ("not true", "somewhat or sometimes true", and "very true or often true"). Responses are categorised into syndrome profiles, and these are subsequently grouped into internalising (emotional reactivity, anxiety/depression, somatic complaints, and withdrawal), externalising (attention problems, aggressive behaviour) and total (internalising, externalising, sleep and other) problem scales. Higher scores indicate increased emotional and behavioural problems. Total scores are classified into a normal range (≤92rd centile, T ≤64), borderline range (93rd-97th centile, T 65-69), and clinical range (≥98th centile, T ≥70). The CBCL is known to have high reliability and validity for measuring children's emotional and behavioural problems.[28]

ASD traits were assessed using the Quantitative Checklist for Autism in Toddlers (Q-CHAT). The Q-CHAT is a parent-completed 25-item questionnaire, in which the child's behaviour is scored on a 5-point (0-4) frequency scale. Higher total scores correspond to a higher frequency of autistic traits. The Q-CHAT shows good test-retest reliability, face validity and specificity, yet poor positive predictive value.[29,30]

Cognitive assessment was performed using the Bayley Scales of Infant and Toddler Development – Third Edition (Bayley-III). The Bayley-III provides scores for a child's overall cognitive, language and motor development. The cognitive standardised composite score was used in this study; scores between 70-85 indicate mild cognitive impairment, and

For peer review only

For peer review only - http://bmjopen.bmj.com/site/about/guidelines.xhtml

scores lower than 70 indicate moderate-severe impairment[31]. Reliability and validity of the Bayley-III have been shown to be robust.[32]

Assessments were carried out by staff experienced in the neurocognitive assessments of toddlers.

Analysis

Descriptive statistics and one-way ANOVA tests were performed in IBM SPSS Statistics for Windows v.25. All other analyses were carried out in Stata v.16.

Multiple imputation (MI) was carried out to account for missing data in CBCL (11/509, 2.24%), Q-CHAT (9/509, 1.8%), maternal EPDS (73/509, 14.3%), maternal BMI (27/509, 5.3%) and IMD rank (3/509, 0.6%). Variables were imputed simultaneously using the 'mi impute chained' procedure that performs imputation by chained equations. The imputation models included all variables that appear in the corresponding analysis models and also had the same structural form as the analysis models. They additionally included all variables correlating with the incomplete variables, as well as all predictors of the probability of a value being missing.[33] Maternal depression and CBCL were imputed using Poisson regression; Q-CHAT, maternal BMI, and the IMD rank were imputed using linear regression. 40 MI datasets were created. To assess the stability of our MI parameters, we extracted the Monte Carlo error of each parameter estimate and examined whether the error for the coefficient was less than 10% of the parameter's standard error estimate. MI estimates were used for the primary analyses and compared to the estimates from complete-case (CC, individuals who had no missing data pre-imputation) analyses. Normal probability plots of residuals from the CC analyses were examined.

The analysis models used multiple linear regression with standard errors that allowed for intragroup correlation and were fitted using the 'mi estimate' procedure, which estimates effects after application of Rubin's rules.[34] For continuous variables, Cohen's f-squared effect sizes were calculated using $f^2 = (R_{AB}^2 - R_A^2)/(1 - R_{AB}^2)$, where R_{AB}^2 is the R-squared value from a regression model that includes the variable of interest as well as all the covariates used in the model, and R_A^2 is the R-squared value from the regression model that includes only the covariates.[35,36] For binary variables, Cohen's f-squared effect sizes were produced after

For peer review only - http://bmjopen.bmj.com/site/about/guidelines.xhtml

estimating first the Cohen's d using the formula: $f^2 = \frac{d}{2k}$, where k is the number of groups. As a measure of dispersion, Cohen's d used the average root mean-square error over the MI datasets. Adjusted R-squared values after MI were extracted after estimating the model with the user-written 'mibeta' command with the 'fisherz' option,[37] which calculates R-squared measures for linear regression with MI data. The significance of the joint effect of the categorical variable parity was assessed using `mi test' which performs Wald tests of composite linear hypotheses.

Primary outcome measures were children's total CBCL raw score and Q-CHAT score. S*econdary outcome measures* were CBCL internalising and externalising scores. The effect of maternal EPDS score was adjusted for IMD rank, maternal age, maternal BMI, maternal parity, pregnancy size, and the following child's variables: gestational age, birth weight, Bayley-III cognitive composite score, and corrected age at assessment. The interaction between gestational age and maternal depression was explored using a complete case analysis in both CBCL and Q-CHAT models. EPDS, CBCL and Q-CHAT scores were compared between term and preterm infants using the complete case dataset.

In order to investigate the specificity of the association between maternal EPDS scores and child's behavioural outcomes (versus cognitive outcomes) we repeated the analyses using the Bayley-III cognitive composite score as primary outcome, with the following confounders: IMD rank, maternal age, maternal BMI, maternal parity, pregnancy size, and the following child's variables: gestational age, birth weight, corrected age at assessment, and Q-CHAT score. CBCL score was not included in the model predicting cognitive outcome, because cognition was not a significant predictor of CBCL (see Results).

As all mothers had their EPDS score measured near term-corrected age, we further investigated the association between time elapsing between baby's birth and mother's EPDS assessment and EPDS score, in order to avoid erroneously identifying 'baby blues' in mothers of term-born infants versus postnatal depression in mothers of preterm infants. This post-hoc analysis was performed using Poisson regression.

For peer review only

Results

509 toddlers were followed up at a median corrected age of 18.4 months (range 17.3 – 24.3 months). 51 (10.0%) of these were twins, and 3 (0.59%) were triplets. 21/509 (4.13%) of mothers scored above a clinical cut-off (≥13) on the EPDS.(26) Demographic data are shown in Table 1. 400 (78.6%) children had complete data. Missing data were imputed and thus all 509 subjects were included in the primary and secondary analyses. One participant was excluded from the cognition analysis after examining the quintiles of the residuals against the theoretical quintiles of a normal distribution. The mean CBCL T score was 46.9 (SD 9.5) (Table 1); 484 (95.1%) of participants had a CBCL score in the normal range, 14 (2.8%) were borderline, and no participants scored in the clinical range. The mean Q-CHAT score was 30.5 (SD 9.3) (Table 1).

Predictors of children's CBCL and Q-CHAT scores after multiple imputation are shown in Table 2. Higher maternal EPDS score was associated with children's higher CBCL Total score (B=0.93, 95% CI 0.43-1.44, $p<0.001$, $f^2=0.05$) and Q-CHAT score (B=0.27, 95% CI 0.03-0.52, p=.031, $f^2=0.01$) (Table 2). These associations are presented graphically in Figure 1 and Figure 2, respectively. Higher maternal EPDS score was associated with both internalising (B=0.22, 95% CI 0.08-0.36, $p<0.01$, $f^2=0.03$) and externalising (B=0.40, 95% CI 0.20-0.61, $p<0.001$, $f^2=0.05$) symptoms in children (Supplementary Table 1 and 2, respectively). Comparison of the imputed model analyses to the complete-case analyses showed that results were consistent for the CBCL model (Supplementary Table 3). Comparison for the Q-CHAT model showed that maternal EPDS was a significant predictor in the imputed model, but not in the complete-case analysis (Supplementary Table 3).

Maternal EPDS scores did not differ between preterm and term groups in the complete dataset ($t(434)=0.11$, p=0.92). CBCL scores ($t(496)=0.95$, p=0.34) and Q-CHAT scores ($t(122.6)=0.50$, p=0.62) did not differ between preterm and term groups in the complete dataset. Maternal EPDS score did not disproportionately affect preterm children with respect to CBCL or Q-CHAT scores (Table 3).

Mothers who gave birth prematurely (<37 weeks gestation) had their EPDS score assessed on average 7.7 weeks later post-delivery than mothers who gave birth at term (preterm participants M=8.9 (SD 4.8), term participants M=1.2 (SD 1.3); $t(99.4)=15.5$, p<.001). The time-lag between birth and EPDS assessment did not predict maternal EPDS score, and there

For peer review only - http://bmjopen.bmj.com/site/about/guidelines.xhtml

was no evidence of a significant interaction between gestation and birth-to-assessment time-lag (Supplementary Table 4 and 5, respectively).

Boys had higher CBCL and Q-CHAT scores than girls. Higher Q-CHAT scores were associated with lower IMD rank (i.e., greater socio-economic deprivation) and lower Bayley-III cognitive composite scores. Parity was not a significant predictor of outcome in any of the models (Table 2).

The mean Bayley III cognitive composite score in our sample was 100 (SD 11.4) (Table 1); this corresponds to the standardised test mean.[31] 480 (94.3%) of participants had a normal cognitive score, 24 (4.7%) had mild impairment, and 5 (1%) had moderate-severe impairment. Predictors of children's cognitive score are shown in Table 4. Maternal EPDS score at term was not associated with toddlers' cognitive outcomes (B=-0.22, 95% CI -0.50-0.05, p=.108) (Table 4).

Discussion

Principal findings

Contrary to our predictions, mothers of preterm infants did not display more depressive symptoms compared to mothers of term infants. Moreover, gestational age not influence the association between maternal depressive symptoms and infants' behavioural outcomes in toddlerhood. These results suggest that preterm birth may not be a vulnerability or plasticity factor with respect to the effect of maternal postnatal depression on infants' behavioural development in the first 18 months of life. However, our results do suggest that more maternal self-reported depressive symptoms shortly after birth are associated with greater toddlers' behavioural problems and ASD traits, but not with cognitive outcomes. Given that fewer than 5% of the mothers in our cohort had EPDS scores above a clinical threshold,[26] our findings indicate that even subclinical depressive symptoms adversely impact children's behavioural outcomes. In addition, our cohort was typically developing with few CBCL scores reaching a concerning threshold; our results could be interpreted within the conceptual framework of mental illness lying on a continuum with typical behavioural traits.[38]

Comparison to prior literature

For peer review only - http://bmjopen.bmj.com/site/about/guidelines.xhtml

The finding that more preterm infants were not disproportionately affected by maternal depressive symptoms supports Hadfield et al.'s findings that maternal distress at 9 months did not differentially impact very preterm (<34 weeks) or late preterm (34-36^{+6} weeks) infants with respect to socioemotional outcomes, although paternal distress did have an impact on very preterm infants' outcomes.[39] However, our results differ from Gueron-Sela et al.'s finding that very preterm (28-33 weeks) 12 month old infants' social outcomes were more influenced by maternal emotional distress at 6 months than term infants' outcomes.[22] The inconsistent findings may be due to methodological differences: for instance, our infant assessment being conducted at 18 months corrected age when social competency is more developed, our assessment of maternal depressive symptoms being in the very early postnatal period, or our use of a screening measure, the Q-CHAT, as a measure of ASD traits. Importantly, the lack of support for a diathesis-stress or differential susceptibility model of maternal mental state on younger preterm infants in our study must be viewed in the context of our results also showing no difference in CBCL and Q-CHAT scores between term and preterm infants. This is in contrast to the existing literature that preterm infants are more likely to develop behavioural problems, such as ADHD, in childhood and adolescence.[19,24] It is possible that the phenotypes of neurodevelopmental and neuropsychiatric disorders assessed with the chosen behavioural measures may not be sufficiently expressed at 18 months corrected age.[40] In addition, as briefly discussed above, much of the existing literature emphasises the risk of extreme (<28 weeks) or very preterm (28-33 weeks) birth on later mental health outcomes,[19,24] whereas only 3.5% and 5.5% of our participants fell within the extreme and very preterm birth group, respectively, and we thus may not have the power to show any subtle effects.

Our results with respect to internalising and externalising behavioural outcomes are in line with previous studies, including large population cohort studies, that show an association between postnatal maternal depression and young children's emotional and behavioural problems.[11] The only previous study investigating this association in infants at 18 months found maternal depression to be associated with internalising and dysregulated behaviour, but not externalising symptoms.[41] This difference between our and Conroy et al.'s findings may have arisen from their exclusion of infants born <36 weeks and their use of a clinical diagnosis of depression for mothers, rather than the dimensional approach we employed. Interestingly, our finding that even subclinical depressive symptoms may adversely impact

For peer review only - http://bmjopen.bmj.com/site/about/guidelines.xhtml

children's behavioural outcomes is in line with recent data showing that low- as well as high-level depressive symptoms are associated with internalising and externalising symptoms in children aged 3 years.[42]

The results showing an association between maternal postnatal depressive symptoms and childhood ASD are less robust and need to be interpreted with caution. Although some prior studies have reported an association between antenatal maternal depression and offspring's ASD,[10,43] and postnatal depression has been suggested as a potential focus of cross-domain studies of ASD,[44] there is no clear aetiological role of maternal postnatal depression in the development of ASD *per se*. Also, given that mothers with ASD are more likely to suffer from perinatal depression than mothers without ASD,[45] and ASD is highly heritable,[46] maternal depression may be a confounder in our observed results.

Strengths & limitations of the study

The strengths of this study lie primarily in its large sample and prospective data collection. Moreover, the use of multiple imputation methodology has facilitated retention of a complete dataset, thus minimising non-response bias and increasing parameter precision. A strength in comparison to prior population cohort studies is that we assessed very early maternal depressive symptoms. Given the complex interplay of biological and environmental factors in the aetiology of mental health disorders, the inclusion of a substantive proportion of preterm infants in our cohort also offers an important insight into the role of preterm birth in influencing mental health outcomes; moreover, our results represent the full gestational spectrum, rather than discrete gestational categories. In addition, using maternal depression as a continuous, rather than dichotomous, variable allows a more nuanced understanding of the role maternal postnatal depressive symptoms may play in influencing children's outcomes.

There are five main limitations to this study. Firstly, differences in birth-to-EPDS-assessment time-lags are a potential confounder, given the time-sensitive nature of early-onset temporary baby blues and later-onset pathological postnatal depression. Mothers of infants born at term were assessed early post-delivery, within the period one would anticipate baby blues to present, whereas mothers of preterm participants were on average assessed later, when postnatal depression predominates.[1,2] Although our post-hoc analyses showed no association between the time elapsed from birth to EPDS assessment and maternal EPDS score, providing reassurance that our assessments of mothers of term-born infants were not inflated

For peer review only - http://bmjopen.bmj.com/site/about/guidelines.xhtml

by the common, temporary symptoms of baby blues, it is however possible that we did not capture the full extent of later-onset depressive symptoms in mothers of term-born infants. This may explain why maternal EPDS scores did not differ between preterm and term groups in our complete dataset analysis, contrary to the current literature.[23] Secondly, a number of important confounders that are likely to affect children's behavioural outcomes were not assessed in this study, including genetic risk for psychiatric disorders,[47] parental psychiatric co-morbidities,[41] chronicity of postnatal depressive symptoms,[42] antenatal maternal depression, paternal depression and subsequent parent-infant attachment, and inter-parental conflict.[11] In this study we did not systematically collect maternal psychiatric history and our focus was on symptoms rather than a diagnosis of depression. Thus, we are unable to conclude whether our observed associations between early postnatal maternal depressive symptoms and children's mental health outcomes are moderated or mediated by other parental factors. Thirdly, whilst our study included a substantive proportion of preterm infants (97/509, 19%), the sample was not random, as preterm children were selectively recruited for the DHCP; indeed, preterm infants are over-represented in our sample when compared to the UK population incidence (7.3%),[48] which may limit the study's generalisability to the general population. Fourthly, the effect sizes of the association between maternal EPDS score and behavioural problems and ASD traits, respectively, were small; this raises questions regarding the clinical significance of our findings and potentially explains some of the inconsistency between this and previous studies. Even within our analyses, the association between maternal depressive symptoms and ASD traits was not observed in our complete case analysis, thus calling into question the validity of this result. It is also important to highlight the continuum of ASD traits that are conceptualised by the Q-CHAT,[29] as well as its poor positive predictive value;[30] the presence of traits does not imply a diagnosis of ASD, and this distinction may also explain the contrast to previous studies. Fifthly, the outcome measures used in this study were parent-completed questionnaires and it is possible that reporting bias with shared method variance may have skewed our results, as maternal depression has been shown to influence reporting of ASD traits,[49] including the Q-CHAT,[50] and CBCL scores.[51]

Implications of our findings

Of greatest importance to clinicians and policymakers is our finding that even *subclinical* maternal depressive symptoms are associated with behavioural outcomes of offspring. This has significant implications for the risk-stratification of women and their babies in the

For peer review only - http://bmjopen.bmj.com/site/about/guidelines.xhtml

postnatal period, during which contact with medical professionals is already established. Identifying high risk families and providing appropriate supportive measures at the early postnatal stage may help to prevent future psychiatric morbidity.

Future research

Further follow-up of large cohorts of preterm and term infants, to an age when behavioural phenotypes may become more pronounced, is needed to investigate whether the long-term developmental trajectories of term and ex-preterm infants are differentially susceptible to changes of postnatal maternal mental health. Such follow-up should use independent, objective assessments of child behavioural outcomes. Further study is also needed to elucidate the role of maternal depression in the aetiology of ASD, controlling for both diagnostic and sub-clinical maternal ASD symptomatology. Finally, it is crucial for future research to elucidate the interplay of biochemical and neurodevelopmental changes that may mediate and confound the translation of environmental exposures into distal behavioural phenotypes.

Conclusion

This prospective longitudinal cohort study found no evidence to support the concept of preterm birth as a vulnerability or plasticity factor with respect to the effect of maternal depressive symptoms on behavioural development. However, we do show that early subclinical maternal postnatal depressive symptoms are associated with behavioural problems in children. This adds to the increasing body of literature indicating the role of subclinical and early postnatal depressive symptoms in the aetiology of childhood mental health disorders. These findings are of great relevance to child and public health, and have potentially significant implications for developing strategies to facilitate effective screening and support for women and children, enabling all to reach their full potential.

<u>Acknowledgements & contributions</u>

We thank all DHCP investigators for their contribution to the study. We thank Dr Oliver Gale-Grant MRes (Centre for the Developing Brain, King's College London; Department of Forensic & Neurodevelopmental Sciences, King's College London) for providing the IMD rank data. We are very grateful to the families who generously took part in this research.

For peer review only - http://bmjopen.bmj.com/site/about/guidelines.xhtml

Conceptualization: SC, ADE, CN; **Methodology:** IK, GV, SC, AP, ADE, CN; **Investigation:** SF, AC; **Data curation:** IK, AL; **Formal analysis:** IK, GV, AP, CN; **Writing – original draft preparation:** IK; **Writing – Review & Editing:** GV, AL, SF, AC, SC, AP, ADE, CN; **Visualisation:** IK, GV; **Funding acquisition:** SC, ADE; **Supervision:** AP, ADE, CN.

References

1. Woody CA, Ferrari AJ, Siskind DJ, Whiteford HA, Harris MG. A systematic review and meta-regression of the prevalence and incidence of perinatal depression. *J Affect Disord*. 2017;219:86-92. doi:10.1016/j.jad.2017.05.003

2. Henshaw C. Mood disturbance in the early puerperium: a review. *Arch Women's Ment Health*. 2003;6(2):s33-s42. doi:10.1007/s00737-003-0004-x

3. Beck CT. Predictors of postpartum depression: an update. *Nurs Res*. 2001;50(5):275-285.

4. Vigod SN, Villegas L, Dennis C-L, Ross LE. Prevalence and risk factors for postpartum depression among women with preterm and low-birth-weight infants: a systematic review. *BJOG Int J Obstet Gynaecol*. 2010;117(5):540-550. doi:10.1111/j.1471-0528.2009.02493.x

5. Blom EA, Jansen PW, Verhulst FC, et al. Perinatal complications increase the risk of postpartum depression. The Generation R Study. *BJOG Int J Obstet Gynaecol*. 2010;117(11):1390-1398. doi:10.1111/j.1471-0528.2010.02660.x

6. Beck CT. Postpartum depression: a metasynthesis. *Qual Health Res*. 2002;12(4):453-472. doi:10.1177/104973202129120016

7. Grace SL, Evindar A, Stewart DE. The effect of postpartum depression on child cognitive development and behavior: a review and critical analysis of the literature. *Arch Womens Ment Health*. 2003;6(4):263-274. doi:10.1007/s00737-003-0024-6

8. Lebel C, Walton M, Letourneau N, Giesbrecht GF, Kaplan BJ, Dewey D. Prepartum and Postpartum Maternal Depressive Symptoms Are Related to Children's Brain Structure in Preschool. *Biol Psychiatry*. 2016;80(11):859-868. doi:10.1016/j.biopsych.2015.12.004

9. Goodman SH, Rouse MH, Connell AM, Broth MR, Hall CM, Heyward D. Maternal depression and child psychopathology: a meta-analytic review. *Clin Child Fam Psychol Rev*. 2011;14(1):1-27. doi:10.1007/s10567-010-0080-1

10. Hagberg KW, Robijn AL, Jick S. Maternal depression and antidepressant use during pregnancy and the risk of autism spectrum disorder in offspring. *Clin Epidemiol*. 2018;10:1599-1612. doi:10.2147/CLEP.S180618

For peer review only - http://bmjopen.bmj.com/site/about/guidelines.xhtml

11. Stein A, Pearson RM, Goodman SH, et al. Effects of perinatal mental disorders on the fetus and child. *The Lancet*. 2014;384(9956):1800-1819. doi:10.1016/S0140-6736(14)61277-0

12. Van Batenburg-Eddes T, Brion MJ, Henrichs J, et al. Parental depressive and anxiety symptoms during pregnancy and attention problems in children: a cross-cohort consistency study. *J Child Psychol Psychiatry*. 2013;54(5):591-600. doi:10.1111/jcpp.12023

13. Barker ED, Jaffee SR, Uher R, Maughan B. The contribution of prenatal and postnatal maternal anxiety and depression to child maladjustment. *Depress Anxiety*. 2011;28(8):696-702. doi:10.1002/da.20856

14. Pearson RM, Evans J, Kounali D, et al. Maternal depression during pregnancy and the postnatal period: risks and possible mechanisms for offspring depression at 18 years. *JAMA Psychiatry*. 2013;70(12):1312-1319. doi:10.1001/jamapsychiatry.2013.2163

15. Kleine I, Falconer S, Roth S, et al. Early postnatal maternal trait anxiety is associated with the behavioural outcomes of children born preterm <33 weeks. *J Psychiatr Res*. 2020;131:160-168. doi:10.1016/j.jpsychires.2020.09.010

16. Feder A, Nestler EJ, Charney DS. Psychobiology and molecular genetics of resilience. *Nat Rev Neurosci*. 2009;10(6):446-457. doi:10.1038/nrn2649

17. Dimitrova R, Pietsch M, Christiaens D, et al. Heterogeneity in brain microstructural development following preterm birth. *Cereb Cortex*. 2020;30(9):4800-4810. doi:10.1093/cercor/bhaa069

18. Fitzallen GC, Sagar YK, Taylor HG, Bora S. Anxiety and Depressive Disorders in Children Born Preterm: A Meta-Analysis. *J Dev Behav Pediatr JDBP*. 2021;42(2):154-162. doi:10.1097/DBP.0000000000000898

19. Allotey J, Zamora J, Cheong-See F, et al. Cognitive, motor, behavioural and academic performances of children born preterm: a meta-analysis and systematic review involving 64 061 children. *BJOG Int J Obstet Gynaecol*. 2018;125(1):16-25. doi:10.1111/1471-0528.14832

20. Jaekel J, Pluess M, Belsky J, Wolke D. Effects of maternal sensitivity on low birth weight children's academic achievement: a test of differential susceptibility versus diathesis stress. *J Child Psychol Psychiatry*. 2015;56(6):693-701. doi:10.1111/jcpp.12331

21. Belsky J, Bakermans-Kranenburg MJ, van IJzendoorn MH. For better and for worse: differential susceptibility to environmental influences. *Curr Dir Psychol Sci*. 2007;16(6):300-304. doi:10.1111/j.1467-8721.2007.00525.x

22. Gueron-Sela N, Atzaba-Poria N, Meiri G, Marks K. The caregiving environment and developmental outcomes of preterm infants: diathesis stress or differential susceptibility effects? *Child Dev*. 2015;86(4):1014-1030. doi:https://doi.org/10.1111/cdev.12359

For peer review only - http://bmjopen.bmj.com/site/about/guidelines.xhtml

23. Carson C, Redshaw M, Gray R, Quigley MA. Risk of psychological distress in parents of preterm children in the first year: evidence from the UK Millennium Cohort Study. *BMJ Open*. 2015;5(12):e007942. doi:10.1136/bmjopen-2015-007942

24. Johnson S, Wolke D. Behavioural outcomes and psychopathology during adolescence. *Early Hum Dev*. 2013;89(4):199-207. doi:10.1016/j.earlhumdev.2013.01.014

25. Ministry of Housing, Communities & Local Government. The English Indices of Deprivation 2019. Published online 2019. https://assets.publishing.service.gov.uk/government/uploads/system/uploads/attachment_data/file/835115/IoD2019_Statistical_Release.pdf

26. Cox JL, Holden JM, Sagovsky R. Detection of postnatal depression: development of the 10-item Edinburgh Postnatal Depression Scale. *Br J Psychiatry*. 1987;150(6):782-786. doi:10.1192/bjp.150.6.782

27. Levis B, Negeri Z, Sun Y, Benedetti A, Thombs BD. Accuracy of the Edinburgh Postnatal Depression Scale (EPDS) for screening to detect major depression among pregnant and postpartum women: systematic review and meta-analysis of individual participant data. *BMJ*. 2020;371. doi:10.1136/bmj.m4022

28. Achenbach TM, Rescorla LA. *Manual for the ASEBA Preschool Forms & Profiles*.; 2000.

29. Allison C, Baron-Cohen S, Wheelwright S, et al. The Q-CHAT (Quantitative Checklist for Autism in Toddlers): a normally distributed quantitative measure of autistic traits at 18-24 months of age: preliminary report. *J Autism Dev Disord*. 2008;38(8):1414-1425. doi:10.1007/s10803-007-0509-7

30. Allison C, Matthews FE, Ruta L, et al. Quantitative Checklist for Autism in Toddlers (Q-CHAT). A population screening study with follow-up: the case for multiple time-point screening for autism. *BMJ Paediatr Open*. 2021;5(1):e000700. doi:10.1136/bmjpo-2020-000700

31. Bayley N. *Bayley Scales of Infant and Toddler Development - Third Edition*. 3rd ed. Harcourt Assessment; 2006.

32. Albers CA, Grieve AJ. Review of Bayley Scales of Infant and Toddler Development--Third Edition. *J Psychoeduc Assess*. 2007;25(2):180-190. doi:10.1177/0734282906297199

33. White IR, Royston P, Wood AM. Multiple imputation using chained equations: Issues and guidance for practice. *Stat Med*. 2011;30(4):377-399. doi:https://doi.org/10.1002/sim.4067

34. Rubin DB. Inference and missing data. *Biometrika*. 1976;63(3):581-592. doi:10.2307/2335739

35. Cohen J. *Statistical Power Analysis for the Behavioral Sciences*. 2nd ed. L. Erlbaum Associates; 1988.

For peer review only

For peer review only - http://bmjopen.bmj.com/site/about/guidelines.xhtml

36. Selya AS, Rose JS, Dierker LC, Hedeker D, Mermelstein RJ. A practical guide to calculating Cohen's f2, a measure of local effect size, from PROC MIXED. *Front Psychol*. 2012;3. doi:10.3389/fpsyg.2012.00111

37. Harel O. The estimation of R 2 and adjusted R 2 in incomplete data sets using multiple imputation. *J Appl Stat*. 2009;36(10):1109-1118. doi:10.1080/02664760802553000

38. Plomin R, Haworth CMA, Davis OSP. Common disorders are quantitative traits. *Nat Rev Genet*. 2009;10(12):872-878. doi:10.1038/nrg2670

39. Hadfield K, O'Brien F, Gerow A. Is level of prematurity a risk/plasticity factor at three years of age? *Infant Behav Dev*. 2017;47:27-39. doi:10.1016/j.infbeh.2017.03.003

40. Egger HL, Emde RN. Developmentally sensitive diagnostic criteria for mental health disorders in early childhood: the diagnostic and statistical manual of mental disorders-IV, the research diagnostic criteria-preschool age, and the diagnostic classification of mental health and developmental disorders of infancy and early childhood-revised. *Am Psychol*. 2011;66(2):95-106. doi:10.1037/a0021026

41. Conroy S, Pariante CM, Marks MN, et al. Maternal psychopathology and infant development at 18 months: the impact of maternal personality disorder and depression. *J Am Acad Child Adolesc Psychiatry*. 2012;51(1):51-61. doi:10.1016/j.jaac.2011.10.007

42. Kingston D, Kehler H, Austin M-P, et al. Trajectories of maternal depressive symptoms during pregnancy and the first 12 months postpartum and child externalizing and internalizing behavior at three years. *PloS One*. 2018;13(4):e0195365. doi:10.1371/journal.pone.0195365

43. Wiggins LD, Rubenstein E, Daniels J, et al. A phenotype of childhood autism is associated with preexisting maternal anxiety and depression. *J Abnorm Child Psychol*. 2019;47(4):731-740. doi:10.1007/s10802-018-0469-8

44. Babb JA, Deligiannidis KM, Murgatroyd CA, Nephew BC. Peripartum depression and anxiety as an integrative cross-domain target for psychiatric preventative measures. *Behav Brain Res*. 2015;0:32-44. doi:10.1016/j.bbr.2014.03.039

45. Pohl AL, Crockford SK, Blakemore M, Allison C, Baron-Cohen S. A comparative study of autistic and non-autistic women's experience of motherhood. *Mol Autism*. 2020;11(1):3. doi:10.1186/s13229-019-0304-2

46. Colvert E, Tick B, McEwen F, et al. Heritability of autism spectrum disorder in a UK population-based twin sample. *JAMA Psychiatry*. 2015;72(5):415-423. doi:10.1001/jamapsychiatry.2014.3028

47. Biel MG. Parental psychiatric symptoms and children's outcomes: toward understanding and responding to intergenerational risk in child psychiatry. *J Am Acad Child Adolesc Psychiatry*. 2018;57(9):632-633. doi:10.1016/j.jaac.2018.06.010

48. National Institute for Health and Care Excellence. Preterm labour and birth (NICE Guideline 25). Published online November 2015. Accessed January 27, 2019. https://www.nice.org.uk/guidance/ng25

For peer review only - http://bmjopen.bmj.com/site/about/guidelines.xhtml

49. Bennett T, Boyle M, Georgiades K, et al. Influence of reporting effects on the association between maternal depression and child autism spectrum disorder behaviors. *J Child Psychol Psychiatry*. 2012;53(1):89-96. doi:10.1111/j.1469-7610.2011.02451.x

50. Goh DA, Gan D, Kung J, et al. Child, Maternal and Demographic Factors Influencing Caregiver-Reported Autistic Trait Symptomatology in Toddlers. *J Autism Dev Disord*. 2018;48(4):1325-1337. doi:10.1007/s10803-018-3471-7

51. Friedlander S, Weiss DS, Traylor J. Assessing the influence of maternal depression on the validity of the Child Behavior Checklist. *J Abnorm Child Psychol*. 1986;14(1):123-133. doi:10.1007/bf00917228

For peer review only

For peer review only - http://bmjopen.bmj.com/site/about/guidelines.xhtml

Table 1: Socio-demographic, maternal and clinical characteristics (n=509)

Variable	Number (%)*
Sex: Male	274 (53.8)
Index of Multiple Deprivation (IMD) quintiles	
1 (least deprived) [a]	65 (12.8)
2	87 (17.2)
3	108 (21.3)
4	173 (34.2)
5 (most deprived)	73 (14.4)
Gestational age at birth (weeks), median [range]	39.7 [20 – 43]
Gestational category	
Preterm (<37 weeks)	97 (19.1)
Term (≥37 weeks)	412 (80.9)
Birthweight (g), median [range]	3290 [450 – 4750]
Multiple pregnancy	54 (10.6)
Maternal parity	
0	332 (65.2)
1	124 (24.4)
2	32 (6.3)
3+	21 (4.2)
Maternal BMI (kg/m^2), median [range]	23.2 [15.3 – 43.6]
Maternal age at infant's birth (years), mean (SD)	34.2 (4.8)
Bayley III cognitive composite score, mean (SD)	100 (11.4)
CBCL total T score, mean (SD)	46.9 (9.5)
Q-CHAT total score, mean (SD)	30.5 (9.3)
EPDS score, median [range]	4 [0 – 28]

[a] Quintile 1 corresponds to the highest, least deprived, IMD rankings.

* unless otherwise specified

For peer review only

Table 2: CBCL and Q-CHAT model predictors using multiple imputation without interaction. (Cf. Supplementary Table 3 for complete case analysis)

	CBCL			Q-CHAT		
	**B [95%CI]**	**p**	**f^2**	**B [95%CI]**	**p**	**f^2**
**Maternal EPDS**	0.93 [0.43, 1.44]	<.001 ***	0.05	0.27 [0.03, 0.52]	.031 *	0.01
**Maternal BMI**	-0.09 [-0.44, 0.26]	.621	-	0.06 [-0.13, 0.24]	.538	-
**Multiple pregnancy**	3.15 [-3.07, 9.37]	.320	-	1.33 [-2.62, 5.28]	.509	-
**Parity**						
**1**	-2.52 [-5.96, 0.93]	.151	-	-2.14 [-4.02, -0.27]	.025 [a]	-
**2**	-3.23 [-9.16, 2.70]	.285	-	0.88 [-1.99, 3.75]	.548	-
**3+**	-1.37 [-8.36, 5.61]	.699	-	-0.49 [-4.57, 3.60]	.815	-
**IMD rank**	-1.48 [-3.33, 0.37]	.117	-	-1.50 [-2.60, -0.40]	.008 **	0.02
**Gestational age at birth (weeks)**	0.10 [-0.65, 0.85]	.786	-	0.26 [-0.17, 0.70]	.233	-
**Birthweight (kg)**	0.56 [-2.65, 3.78]	.731	-	-1.24 [-2.93, 0.46]	.151	-
**Sex:female**	-4.14 [-6.96, -1.31]	.004 **	0.06	-1.95 [-3.42, -0.48]	.009 **	0.05
**Corrected age at assessment (months)**	-0.90 [-2.17, 0.37]	.166	-	-0.16 [-0.91, 0.59]	.677	-
**Cognition**	-0.05 [-0.20, 0.09]	.467	-	-0.27 [-0.35, -0.20]	<.001 ***	0.12

$p<0.05$ *; $p<0.01$ **; $p<0.001$ ***
CBCL model adjusted $R^2 = 0.0676$. Q-CHAT model adjusted $R^2 = 0.193$.
B = unstandardised coefficient.
CBCL = Child Behaviour Checklist score at 18 months. Q-CHAT = Quantitative Checklist for Autism in Toddlers score at 18 months. Maternal EPDS = maternal Edinburgh Postnatal Depression Scale score at term-corrected age. Multiple pregnancy = dummy variable of twin/triplet pregnancy. Parity = dummy variable, one/two/three+ previous child(ren). Corrected age at assessment (months) = age at behavioural assessment, corrected for gestational age. Cognition = infant Bayley III score at 18 months.
[a] Wald test of whole parity variable in Q-CHAT model: $F(3, 476.9) = 1.88$, $p = .133$
Effect size (Cohen's f^2, calculated from squared part correlations for predictors significant to 0.05): 0.02 = small, 0.15 = medium and 0.35 = large.[25]
- indicates data not given, as predictor not significant to 0.05.

For peer review only

For peer review only - http://bmjopen.bmj.com/site/about/guidelines.xhtml

Table 3: CBCL and Q-CHAT model predictors using complete case analysis with interaction of 'EPDS x term'.

	CBCL		Q-CHAT	
	**B [95%CI]**	**p**	**B [95%CI]**	**p**
**Maternal EPDS**	0.89 [-0.24, 2.02]	.121	0.24 [-0.28, 0.75]	.365
**Maternal BMI**	-0.01 [-0.39, 0.37]	.955	0.00 [-0.16, 0.17]	.982
**Multiple pregnancy**	1.76 [-6.65, 10.17]	.681	0.97 [-2.07, 4.01]	.532
**Parity**				
**1**	-2.75 [-6.49, 0.99]	.149	-1.42 [-3.30, 0.46]	.139
**2**	-3.49 [-10.36, 3.37]	.317	0.16 [-2.84, 3.16]	.917
**3+**	-1.17 [-9.69, 7.35]	.788	-1.13 [-4.24, 1.98]	.476
**IMD rank**	-1.41 [-3.54, 0.73]	.195	-1.68 [-2.64, -0.72]	.001 **
**Gestation: term**	1.25 [-8.34, 10.85]	.797	2.64 [-1.74, 7.02]	.236
**Birthweight (kg)**	-1.01 [-4.08, 2.05]	.516	-2.25 [-3.73, -0.78]	.003 **
**Sex: female**	-4.64 [-7.83, -1.44]	.005 **	-2.22 [-3.72, -0.71]	.004 **
**Corrected age at assessment (months)**	-0.83 [-2.27, 0.62]	.261	-0.39 [-1.18, 0.04]	.335
**Cognition**	-0.03 [-0.20, 0.14]	.720	-0.22 [-0.29, -.0.15]	<.001 ***
**EPDS x gestation:term**	-0.01 [-1.30, 1.28]	.991	-0.02 [-0.60, 0.56]	.950

p<0.05 *; p<0.01 **; p<0.001 ***
CBCL model adjusted $R^2 = 0.0865$. Q-CHAT model adjusted $R^2 = 0.215$.
B = unstandardised coefficient.
CBCL = Child Behaviour Checklist score at 18 months. Q-CHAT = Quantitative Checklist for Autism in Toddlers score at 18 months. Maternal EPDS = maternal Edinburgh Postnatal Depression Scale score at term-corrected age. Multiple pregnancy = dummy variable of twin/triplet pregnancy. Parity = dummy variable, one/two/three+ previous child(ren). Gestation: term = dummy variable, term (≥37 weeks) vs preterm (<37 weeks) gestation at birth. Corrected age at assessment (months) = age at behavioural assessment, corrected for gestational age. Cognition = infant Bayley III score at 18 months. EPDS x gestation:term = interaction term between maternal EPDS score and term gestation at birth.

For peer review only - http://bmjopen.bmj.com/site/about/guidelines.xhtml

Table 4: Cognition model predictors using multiple imputation.

	B [95%CI]	p
**Maternal EPDS**	-0.22 [-0.50, 0.05]	.108
**Maternal BMI**	-0.32 [-0.52, -0.13]	.001 **
**Multiple pregnancy**	1.65 [-2.49, 5.79]	.433
**Parity**		
**1**	-0.46 [-2.67, 1.76]	.686
**2**	-3.47 [-6.69, -0.25]	.035 [a]
**3+**	-4.57 [-9.53, 0.40]	.072
**IMD rank**	1.43 [0.37, 2.50]	.009 **
**Gestational age at birth (weeks)**	0.45 [-0.07, 0.96]	.091
**Birthweight (kg)**	0.81 [-1.24, 2.87]	.436
**Sex: female**	1.99 [0.24, 3.74]	.026 *
**Corrected age at assessment (months)**	-0.75 [-1.59, 0.08]	.075
**Q-CHAT score**	-0.39 [-0.50, -0.28]	<.001 ***

$p<0.05$ *; $p<0.01$ **; $p<0.001$ ***
Adjusted $R^2 = 0.231$
B = unstandardised coefficient.
Maternal EPDS = maternal Edinburgh Postnatal Depression Scale score at term-corrected age. Multiple pregnancy = dummy variable of twin/triplet pregnancy. Parity = dummy variable, one/two/three+ previous child(ren). Corrected age at assessment (months) = age at behavioural assessment, corrected for gestational age. Cognition = infant Bayley III score at 18 months. Q-CHAT score = infant's Q-CHAT score at 18 month assessment.
[a] Wald test of whole parity variable in cognition model: F(3, 482.9)=2.41, p=0.067

Fig.1 Children's predicted CBCL scores at 18 months are positively correlated to the maternal EPDS score at term corrected age

Fig.2 Children's predicted Q-CHAT scores at 18 months are positively correlated to the maternal EPDS score at term corrected age

For peer review only - http://bmjopen.bmj.com/site/about/guidelines.xhtml

For peer review only

Supplemental material

Supplementary Table 1: CBCL internalising symptom model predictors using multiple imputation

Supplementary Table 2: CBCL externalising symptom model predictors using multiple imputation

Supplementary Table 3: CBCL and Q-CHAT model predictors using complete case analysis without interaction.

Supplementary Table 4: EPDS score predictors

Supplementary Table 5: EPDS score predictors including interaction ‘term x time-lag’

For peer review only

Supplementary Table 1: CBCL internalising symptom model predictors using multiple imputation

	B [95%CI]	p	f^2
**Maternal EPDS**	0.22 [0.08, 0.36]	.003 **	0.03
**Maternal BMI**	-0.04 [-0.13, 0.06]	.436	-
**Multiple pregnancy**	0.58 [-1.10, 2.27]	.497	-
**Parity**			
**1**	-0.37 [-1.41, 0.67]	.487	-
**2**	-1.33 [-3.09, 0.42]	.136	-
**3+**	0.64 [-1.34, 2.62]	.524	-
**IMD rank**	-0.41 [-0.91, 0.10]	.115	-
**Gestational age at birth (weeks)**	0.22 [-0.00, 0.44]	.053	-
**Birthweight (kg)**	-0.97 [-1.90, -0.05]	.038 *	0.005
**Sex: female**	-0.51 [-1.35, 0.33]	.232	-
**Corrected age at assessment (months)**	0.02 [-0.41, 0.45]	.923	-
**Cognition**	-0.05 [-0.10, -0.01]	.016 *	0.01

$p<0.05$ *; $p<0.01$ **; $p<0.001$ ***
Adjusted $R^2 = 0.0566$.
B = unstandardised coefficient.
Outcome variable = Child Behaviour Checklist internalising sub-score at 18 months. Maternal EPDS = maternal Edinburgh Postnatal Depression Scale score at term-corrected age. Multiple pregnancy = dummy variable of twin/triplet pregnancy. Parity = dummy variable, one/two/three+ previous child(ren). Corrected age at assessment (months) = age at behavioural assessment, corrected for gestational age. Cognition = infant Bayley III score at 18 months.
Effect size (Cohen's f^2, calculated from squared part correlations for predictors significant to 0.05): 0.02 = small, 0.15 = medium and 0.35 = large.[1]
- indicates data not given, as predictor not significant to 0.05.

For peer review only

Supplementary Table 2: CBCL externalising symptom model predictors using multiple imputation

	B [95%CI]	p	f^2
**Maternal EPDS**	0.40 [0.20, 0.61]	<.001 ***	0.05
**Maternal BMI**	0.01 [-0.17, 0.15]	.933	-
**Multiple pregnancy**	2.51 [-0.29, 5.31]	.079	-
**Parity**			
**1**	-1.06 [-2.53, 0.42]	.160	-
**2**	-0.61 [-3.55, 2.33]	.682	-
**3+**	-0.96 [-4.47, 2.56]	.593	-
**IMD rank**	-0.24 [-1.11, 0.63]	.585	-
**Gestational age at birth (weeks)**	-0.07 [-0.40, 0.27]	.701	-
**Birthweight (kg)**	1.03 [-0.38, 2.44]	.153	-
**Sex: female**	-1.80 [-3.07, -0.53]	.006 **	0.06
**Corrected age at assessment (months)**	-0.40 [-0.95, 0.16]	.161	-
**Cognition**	0.03 [-0.03, 0.10]	.322	-

p<0.05 *; p<0.01 **; p<0.001 ***
Adjusted R^2 = 0.0612.
B = unstandardised coefficient.
Outcome variable = Child Behaviour Checklist externalising sub-score at 18 months. Maternal EPDS = maternal Edinburgh Postnatal Depression Scale score at term-corrected age. Multiple pregnancy = dummy variable of twin/triplet pregnancy. Parity = dummy variable, one/two/three+ previous child(ren). Corrected age at assessment (months) = age at behavioural assessment, corrected for gestational age. Cognition = infant Bayley III score at 18 months.
Effect size (Cohen's f^2, calculated from squared part correlations for predictors significant to 0.05): 0.02 = small, 0.15 = medium and 0.35 = large.[1]
- indicates data not given, as predictor not significant to 0.05.

For peer review only

Supplementary Table 3: CBCL and Q-CHAT model predictors using complete case analysis without interaction.

	CBCL		Q-CHAT	
	**B [95%CI]**	**p**	**B [95%CI]**	**p**
**Maternal EPDS**	0.88 [0.35, 1.41]	.001 **	0.21 [-0.02, 0.44]	.069
**Maternal BMI**	-0.01 [-0.38, 0.37]	.963	0.01 [-0.16, 0.17]	.930
**Multiple pregnancy**	1.50 [-6.87, 9.87]	.724	0.43 [-2.50, 3.37]	.772
**Parity**				
**1**	-2.83 [-6.60, 0.94]	.141	-1.54 [-3.41, 0.34]	.108
**2**	-3.49 [-10.3, 3.35]	.316	0.13 [-2.85, 3.10]	.933
**3+**	-1.38 [-10.0, 7.30]	.755	-1.43 [-4.50, 1.64]	.360
**IMD rank**	-1.44 [-3.56, 0.67]	.181	-1.75 [-2.70, -0.79]	<.001 ***
**Gestational age at birth (weeks)**	0.01 [-0.90, 0.91]	.987	0.10 [-0.33, 0.54]	.639
**Birthweight (kg)**	-0.65 [-4.38, 3.08]	.733	-1.81 [-3.63, 0.00]	.050
**Sex: female**	-4.57 [-7.82, -1.31]	.006 **	-2.12 [-3.60, -0.64]	.005 **
**Corrected age at assessment (months)**	-0.84 [-2.26, 0.59]	.247	-0.41 [-1.18, 0.35]	.290
**Cognition**	-0.03 [-0.20, 0.13]	.689	-0.23 [-0.30, -0.15]	<.001 ***

p<0.05 *; p<0.01 **; p<0.001 ***
CBCL adjusted R^2 = 0.0862. Q-CHAT adjusted R^2 = 0.2103.
B = unstandardised coefficient.
CBCL = Child Behaviour Checklist externalising sub-score at 18 months. Q-CHAT = Quantitative Checklist for Autism in Toddlers score at 18 months. Maternal EPDS = maternal Edinburgh Postnatal Depression Scale score at term-corrected age. Multiple pregnancy = dummy variable of twin/triplet pregnancy. Parity = dummy variable, one/two/three+ previous child(ren). Gestation (weeks) = dummy variable: 34-36+6 weeks and ≥37 weeks gestation at birth. Corrected age at assessment (months) = age at behavioural assessment, corrected for gestational age. Cognition = infant Bayley III score at 18 months.

For peer review only

Supplementary Table 4: EPDS score predictors

	IRR [95%CI]	p
**Time-lag (weeks)**	1.01 [0.97, 1.05]	.647
**Gestation:term**	0.91 [0.64, 1.31]	.627
**IMD rank**	1.00 [1.00, 1.00]	.103
**Multiple pregnancy**	0.66 [0.46, 0.96]	.031 *
**Parity**		
**1**	0.79 [0.66, 0.95]	.011 *
**2**	0.87 [0.60, 1.28]	.491
**3+**	0.84 [0.54, 1.31]	.445
**Birthweight (kg)**	0.98 [0.83, 1.17]	.847
**Sex:female**	1.13 [0.98, 1.31]	.098

p<0.05 *; p<0.01 **; p<0.001 ***
Pseudo $R^2 = 0.0228$
IRR = incidence rate ratio
Outcome variable = maternal Edinburgh Postnatal Depression Scale (EPDS) score at term-corrected age. Time-lag (weeks) = time in weeks between birth and EPDS assessment. Gestation:term = dummy variable, term (≥37 weeks) vs preterm (<37 weeks) gestation at birth. Multiple pregnancy = dummy variable of twin/triplet pregnancy. Parity = dummy variable, one/two/three+ previous child(ren).

For peer review only

Supplementary Table 5: EPDS score predictors including interaction 'term x time-lag'

	IRR [95%CI]	p
**Time-lag (weeks)**	1.00 [0.97, 1.04]	.823
**Gestation: term**	0.88 [0.58, 1.34]	.553
**IMD rank**	1.00 [1.00, 1.00]	.104
**Multiple pregnancy**	0.66 [0.46, 0.96]	.029 *
**Parity**		
**1**	0.79 [0.66, 0.95]	.010 *
**2**	0.87 [0.60, 1.28]	.480
**3+**	0.85 [0.55, 1.31]	.458
**Birthweight (kg)**	0.97 [0.83, 1.15]	.756
**Sex:female**	1.13 [0.98, 1.30]	.100
**Term x time-lag (weeks)**	1.01 [0.94, 1.10]	.735

$p<0.05$ *; $p<0.01$ **; $p<0.001$ ***
Pseudo $R^2 = 0.0230$
IRR = incidence rate ratio
Outcome variable = maternal Edinburgh Postnatal Depression Scale (EPDS) score at term-corrected age. Time-lag (weeks) = time in weeks between birth and EPDS assessment. Gestation: term = dummy variable, term (≥37 weeks) vs preterm (<37 weeks) gestation at birth. Multiple pregnancy = dummy variable of twin/triplet pregnancy. Parity = dummy variable, one/two/three+ previous child(ren). Term x time-lag (weeks): interaction term between term gestation at birth and time-lag between birth and maternal EPDS assessment.

For peer review only

Supplemental reference list

1. Cohen J. *Statistical Power Analysis for the Behavioral Sciences*. 2nd ed. L. Erlbaum Associates; 1988.

For peer review only

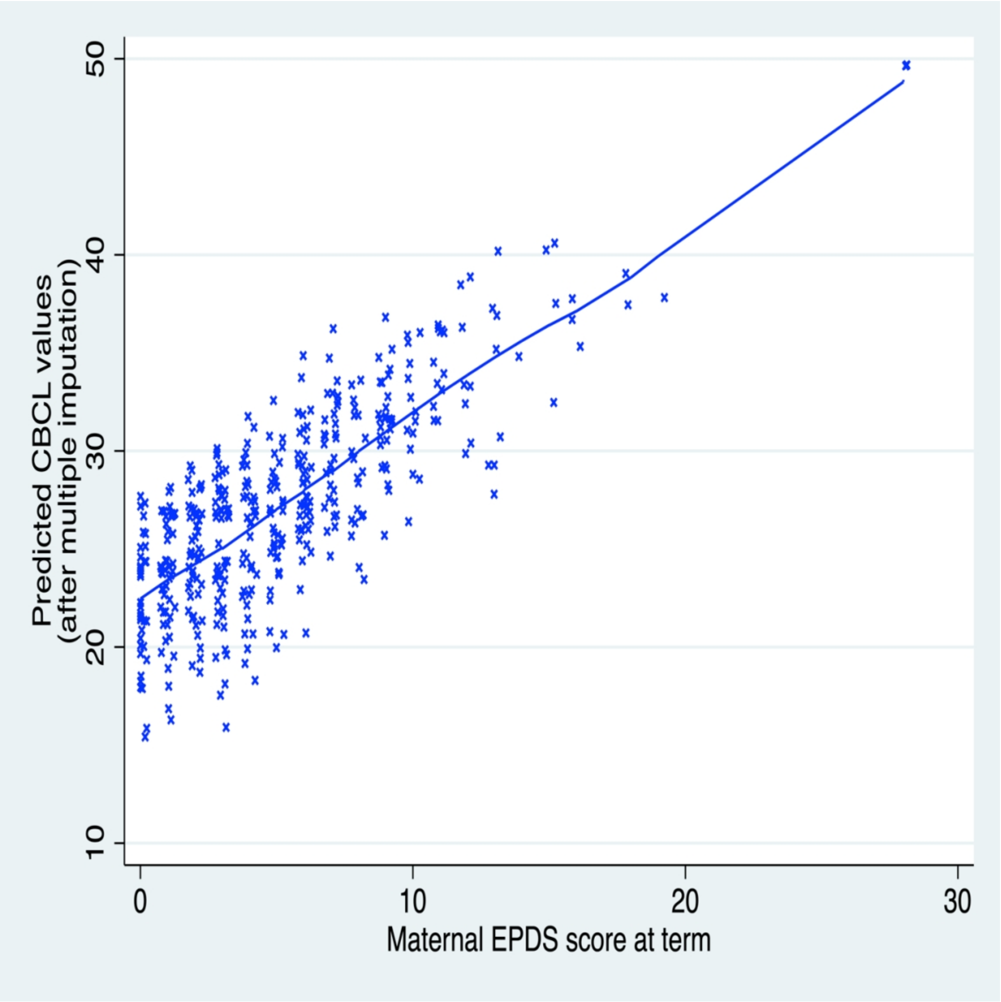

Fig.1 Children's predicted CBCL scores at 18 months are positively correlated to the maternal EPDS score at term-equivalent age

158x158mm (220 x 220 DPI)

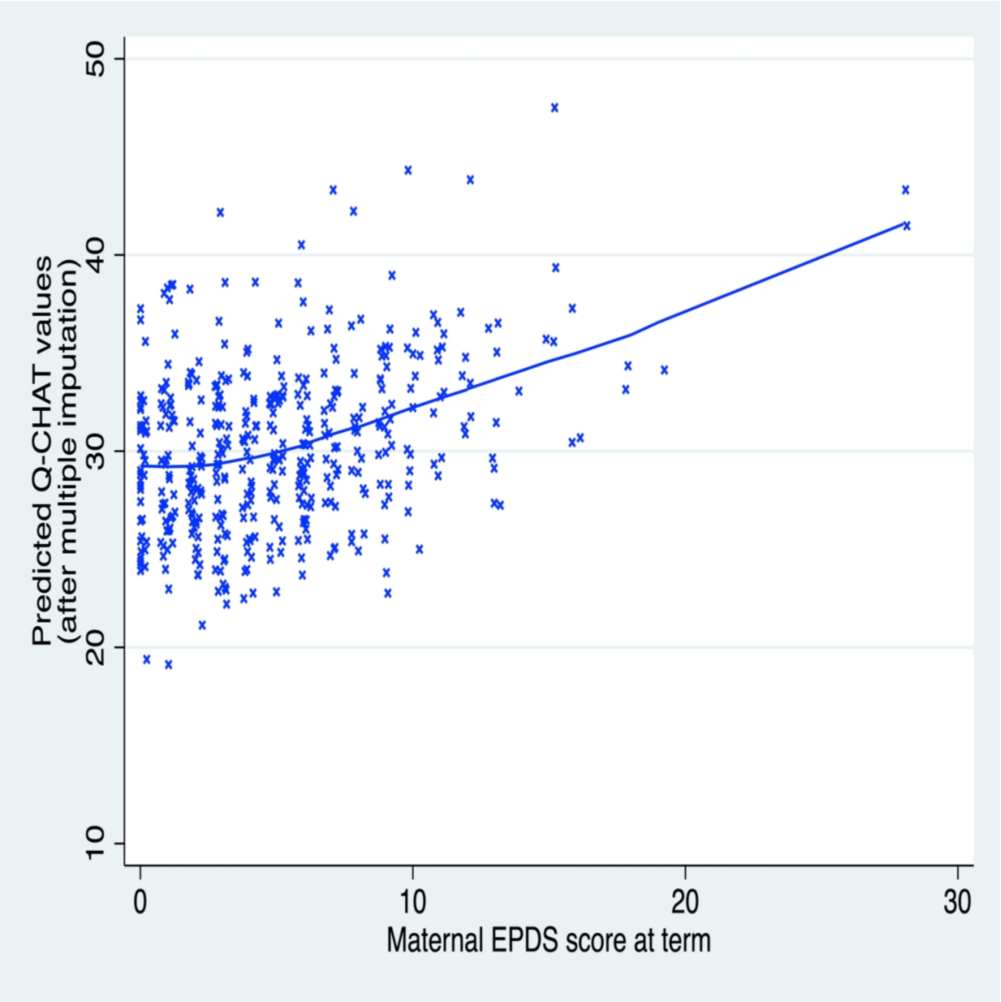

Fig.2 Children's predicted Q-CHAT scores at 18 months are positively correlated to the maternal EPDS score at term-equivalent age.

158x158mm (220 x 220 DPI)

STROBE Statement—Checklist of items that should be included in reports of ***cohort studies***

	Item No	Recommendation	Page No
**Title and abstract**	1	(*a*) Indicate the study's design with a commonly used term in the title or the abstract<br>(*b*) Provide in the abstract an informative and balanced summary of what was done and what was found	1, 2
**Introduction**			
Background/rationale	2	Explain the scientific background and rationale for the investigation being reported	5
Objectives	3	State specific objectives, including any prespecified hypotheses	6
**Methods**			
Study design	4	Present key elements of study design early in the paper	6
Setting	5	Describe the setting, locations, and relevant dates, including periods of recruitment, exposure, follow-up, and data collection	6
Participants	6	(*a*) Give the eligibility criteria, and the sources and methods of selection of participants. Describe methods of follow-up<br>(*b*) For matched studies, give matching criteria and number of exposed and unexposed	6
Variables	7	Clearly define all outcomes, exposures, predictors, potential confounders, and effect modifiers. Give diagnostic criteria, if applicable	6, 7
Data sources/ measurement	8*	For each variable of interest, give sources of data and details of methods of assessment (measurement). Describe comparability of assessment methods if there is more than one group	7
Bias	9	Describe any efforts to address potential sources of bias	8
Study size	10	Explain how the study size was arrived at	6
Quantitative variables	11	Explain how quantitative variables were handled in the analyses. If applicable, describe which groupings were chosen and why	8
Statistical methods	12	(*a*) Describe all statistical methods, including those used to control for confounding<br>(*b*) Describe any methods used to examine subgroups and interactions<br>(*c*) Explain how missing data were addressed<br>(*d*) If applicable, explain how loss to follow-up was addressed<br>(*e*) Describe any sensitivity analyses	8, 9
**Results**			
Participants	13*	(a) Report numbers of individuals at each stage of study—eg numbers potentially eligible, examined for eligibility, confirmed eligible, included in the study, completing follow-up, and analysed<br>(b) Give reasons for non-participation at each stage<br>(c) Consider use of a flow diagram	10
Descriptive data	14*	(a) Give characteristics of study participants (eg demographic, clinical, social) and information on exposures and potential confounders<br>(b) Indicate number of participants with missing data for each variable of interest<br>(c) Summarise follow-up time (eg, average and total amount)	10
Outcome data	15*	Report numbers of outcome events or summary measures over time	10

For peer review only

For peer review only - http://bmjopen.bmj.com/site/about/guidelines.xhtml

Main results	16	(*a*) Give unadjusted estimates and, if applicable, confounder-adjusted estimates and their precision (eg, 95% confidence interval). Make clear which confounders were adjusted for and why they were included<br>(*b*) Report category boundaries when continuous variables were categorized<br>(*c*) If relevant, consider translating estimates of relative risk into absolute risk for a meaningful time period	10
Other analyses	17	Report other analyses done—eg analyses of subgroups and interactions, and sensitivity analyses	10, 11
**Discussion**			
Key results	18	Summarise key results with reference to study objectives	11
Limitations	19	Discuss limitations of the study, taking into account sources of potential bias or imprecision. Discuss both direction and magnitude of any potential bias	13, 14
Interpretation	20	Give a cautious overall interpretation of results considering objectives, limitations, multiplicity of analyses, results from similar studies, and other relevant evidence	14, 15
Generalisability	21	Discuss the generalisability (external validity) of the study results	14
**Other information**			
Funding	22	Give the source of funding and the role of the funders for the present study and, if applicable, for the original study on which the present article is based	3

*Give information separately for exposed and unexposed groups.

Note: An Explanation and Elaboration article discusses each checklist item and gives methodological background and published examples of transparent reporting. The STROBE checklist is best used in conjunction with this article (freely available on the Web sites of PLoS Medicine at http://www.plosmedicine.org/, Annals of Internal Medicine at http://www.annals.org/, and Epidemiology at http://www.epidem.com/). Information on the STROBE Initiative is available at http://www.strobe-statement.org.

For peer review only

BMJ Open

Postnatal maternal depressive symptoms and behavioural outcomes in term- and preterm-born toddlers: a longitudinal UK community cohort study.

Journal:	*BMJ Open*
Manuscript ID	bmjopen-2021-058540.R1
Article Type:	Original research
Date Submitted by the Author:	19-Feb-2022
Complete List of Authors:	Kleine, Ira; King's College London, Centre for the Developing Brain, School of Bioengineering and Imaging Sciences, Faculty of Life Sciences & Medicine<br>Vamvakas, George; King's College London, Department of Biostatistics and Health Informatics, Institute of Psychiatry, Psychology and Neuroscience<br>Lautarescu, Alexandra; King's College London, Centre for the Developing Brain, School of Bioengineering and Imaging Sciences, Faculty of Life Sciences & Medicine; King's College London, Forensic and Neurodevelopmental Sciences, Institute of Psychiatry, Psychology and Neuroscience<br>Falconer, Shona; King's College London, Centre for the Developing Brain, School of Bioengineering and Imaging Sciences, Faculty of Life Sciences & Medicine<br>Chew, Andrew; King's College London, Centre for the Developing Brain, School of Bioengineering and Imaging Sciences, Faculty of Life Sciences & Medicine<br>Counsell, Serena ; King's College London, Centre for the Developing Brain, School of Bioengineering and Imaging Sciences, Faculty of Life Sciences & Medicine<br>Pickles, Andrew; King's College London, Department of Biostatistics and Health Informatics, Institute of Psychiatry, Psychology and Neuroscience<br>Edwards, David; King's College London, Centre for the Developing Brain, School of Bioengineering and Imaging Sciences, Faculty of Life Sciences & Medicine<br>Nosarti, Chiara; King's College London, Centre for the Developing Brain, School of Bioengineering and Imaging Sciences, Faculty of Life Sciences & Medicine; King's College London, Department of Child and Adolescent Psychiatry, Institute of Psychiatry, Psychology and Neuroscience
**Primary Subject Heading**:	Mental health
Secondary Subject Heading:	Paediatrics
Keywords:	MENTAL HEALTH, Child & adolescent psychiatry < PSYCHIATRY, Developmental neurology & neurodisability < PAEDIATRICS, Depression & mood disorders < PSYCHIATRY, NEONATOLOGY

SCHOLARONE™
Manuscripts

I, the Submitting Author has the right to grant and does grant on behalf of all authors of the Work (as defined in the below author licence), an exclusive licence and/or a non-exclusive licence for contributions from authors who are: i) UK Crown employees; ii) where BMJ has agreed a CC-BY licence shall apply, and/or iii) in accordance with the terms applicable for US Federal Government officers or employees acting as part of their official duties; on a worldwide, perpetual, irrevocable, royalty-free basis to BMJ Publishing Group Ltd ("BMJ") its licensees and where the relevant Journal is co-owned by BMJ to the co-owners of the Journal, to publish the Work in this journal and any other BMJ products and to exploit all rights, as set out in our licence.

The Submitting Author accepts and understands that any supply made under these terms is made by BMJ to the Submitting Author unless you are acting as an employee on behalf of your employer or a postgraduate student of an affiliated institution which is paying any applicable article publishing charge ("APC") for Open Access articles. Where the Submitting Author wishes to make the Work available on an Open Access basis (and intends to pay the relevant APC), the terms of reuse of such Open Access shall be governed by a Creative Commons licence – details of these licences and which Creative Commons licence will apply to this Work are set out in our licence referred to above.

Other than as permitted in any relevant BMJ Author's Self Archiving Policies, I confirm this Work has not been accepted for publication elsewhere, is not being considered for publication elsewhere and does not duplicate material already published. I confirm all authors consent to publication of this Work and authorise the granting of this licence.

For peer review only

Postnatal maternal depressive symptoms and behavioural outcomes in term- and preterm-born toddlers: a longitudinal UK community cohort study.

Kleine, I[a] (MBBS); Vamvakas, G[b] (MSc); Lautarescu A[a,c] (MPhil); Falconer, S[a] (PhD); Chew A[a] (MD); Counsell SJ[a] (PhD); Pickles, A[b] (FMedSci); Edwards, AD[a] (FMedSci); Nosarti, C[a,d] (PhD).

[a] Centre for the Developing Brain, School of Bioengineering and Imaging Sciences, Faculty of Life Sciences & Medicine, King's College London, St Thomas' Hospital, London SE1 7EH, UK.

[b] Department of Biostatistics and Health Informatics, Institute of Psychiatry, Psychology and Neuroscience, King's College London, London SE5 8AF, UK.

[c] Forensic and Neurodevelopmental Sciences, Institute of Psychiatry, Psychology and Neuroscience, King's College London, London SE5 8AF, UK.

[d] Department of Child and Adolescent Psychiatry, Institute of Psychiatry, Psychology and Neuroscience, King's College London, London SE5 8AF, UK.

Post-publication corresponding author's contact information

Prof Chiara Nosarti

Centre for the Developing Brain, School of Bioengineering and Imaging Sciences

King's College London

St Thomas' Hospital

London

SE1 7EH

UK

chiara.nosarti@kcl.ac.uk

+44 (0)207 848 0133

For peer review only

Abstract

Objectives: To examine the association between maternal depressive symptoms in the immediate postnatal period and offspring's behavioural outcomes in a large cohort of term- and preterm-born toddlers.

Design and Participants: Data were drawn from the Developing Human Connectome Project. Maternal postnatal depressive symptoms were assessed at term-equivalent age, and children's outcomes were evaluated at a median corrected age of 18.4 months (range 17.3 – 24.3).

Exposure and outcomes: Preterm birth was defined as <37 weeks completed gestation. Maternal depressive symptoms were assessed with the Edinburgh Postnatal Depression Scale (EPDS). Toddlers' outcome measures were parent-rated Child Behaviour Checklist $1^{1/2}$-5 Total (CBCL) and Quantitative Checklist for Autism in Toddlers (Q-CHAT) scores. Toddlers' cognition was assessed with the Bayley Scales of Infant and Toddler Development – Third Edition (Bayley-III).

Results: Higher maternal EPDS scores were associated with toddlers' higher CBCL (B=0.93, 95% CI 0.43-1.44, p<0.001, f^2=0.05) and Q-CHAT scores (B=0.27, 95% CI 0.03-0.52, p=.031, f^2=0.01). Higher maternal EPDS scores were not associated with toddlers' cognitive outcomes. Maternal EPDS, toddlers' CBCL and Q-CHAT scores did not differ between preterm (n=97; 19.1% of the total sample) and term participants.

Conclusions: Our findings indicate that children whose mothers reported increased depressive symptoms in the early postnatal period, including subclinical symptoms, exhibit more parent-reported behavioural problems in toddlerhood. These associations were independent of gestational age. Further research is needed to confirm the clinical significance of these findings.

For peer review only - http://bmjopen.bmj.com/site/about/guidelines.xhtml

Strengths and limitations of this study

- Prospective study with a large sample, using multiple imputation to reduce non-response bias.
- Maternal depressive symptoms assessed as a continuous variable, providing more nuanced information about the significance of subclinical symptoms.
- Maternal depressive symptoms assessed earlier than in previous studies, enabling recognition of early screening opportunities for families.
- Potential common method variance bias through parent-completed child behavioural assessments.
- Unknown paternal and parental factors, such as comorbid psychiatric conditions, that may confound our findings.

Keywords

Postpartum Depression; Child Development; Autism; Mental Health; Child Behavior; Mood Disorder; Preterm Birth.

Declarations

Funding

The DHCP project was funded by the European Research Council under the European Union Seventh Framework Programme (FR/2007-2013)/ERC Grant Agreement no. 319456. The authors acknowledge infrastructure support from the National Institute for Health Research Mental Health Biomedical Research Centre at South London, Maudsley NHS Foundation Trust, King's College London, the National Institute for Health Research Mental Health Biomedical Research Centre at Guys, and St Thomas' Hospitals NHS Foundation Trust. The study was also supported in part by the Engineering and Physical Sciences Research Council / Wellcome Trust Centre for Medical Engineering at King's College London (grant WT 203148/Z/16/Z) and the Medical Research Council (UK) (grants MR/K006355/1 and MR/L011530/1) and the MRC Centre for Neurodevelopmental Disorders at King's College London. AP receives a NIHR SI award (NF-SI-0617-10120). AL is supported by the UK Medical Research Council (MR/N013700) and King's College London member of the MRC Doctoral Training Partnership in Biomedical Sciences. The views expressed are those of the authors and not necessarily those of the NHS, the NIHR, or the Department of Health and Social Care.

For peer review only - http://bmjopen.bmj.com/site/about/guidelines.xhtml

Conflict of interest / Competing interests

ADE received financial support from the EU-AIMS-Trials (European Research Council under the European Union Seventh Framework Programme) as co-Principal Investigator. ADE received consulting fees from Chiesi Farmaceutici (advice on neuroprotection in newborn infants) and Medtronix (unpaid participation in scientific advice committee). ADE has a patent on Xenon as an organ protectant (No P023708WO). ADE was Chair of the Data Monitoring and Ethics Committee for the Baby-Oscar Trial, and served on the Data Monitoring and Ethics Committee for the PAEN Trial.

There are no other relationships or activities that could appear to have influenced the submitted work.

Availability of data and material

Research data are available upon reasonable request.

Code availability

Not applicable.

Ethics approval

This study was approved by the UK National Research Ethics Authority (14/LO/1169) and conducted in accordance with the World Medical Association's Code of Ethics (Declaration of Helsinki).

Consent to participate

Written informed consent was given by children's carer(s) at recruitment into the study.

Consent for publication

Not applicable.

For peer review only - http://bmjopen.bmj.com/site/about/guidelines.xhtml

Introduction

Postnatal depression affects approximately 12% of mothers worldwide.[1] In contrast to 'baby blues', which is a state of emotional lability that affects between 13.7%-76.0% of women in the first few days after birth and typically resolves spontaneously within two weeks,[2] postnatal depression is more severe and starts in the first few months post-partum[1]. Stressful life events have been linked to a heightened risk of developing postnatal depression;[3] for example, mothers of preterm infants have a significantly higher risk of postpartum depression compared to mothers of term infants,[4] likely due to heightened stress associated with perinatal complications.[5]

Women with postnatal depression tend to be less responsive to their baby's needs and to display less affection.[6] Therefore, in the short-term postpartum depression may affect mother-infant interactions[7] and in the long-term it may lead to alterations in brain development,[8] emotional difficulties,[9] less secure attachment, cognitive and behavioural problems in childhood, and a possible increased risk of autism spectrum disorder (ASD).[10,11] Large cohort studies, such as the Avon Longitudinal Study of Parents and Children (ALSPAC), have shown that these associations are even evident when maternal depression is measured on a continuum of symptoms rather than a dichotomous diagnosis,[12–14] supporting the notion that elevated sub-diagnostic psychiatric symptoms can also negatively impact on children's development.[15]

Studies investigating the underlying causes that may link maternal postnatal depression to child outcomes have implicated several biological and environmental variables. For instance, genetic and epigenetic factors have been shown to both mediate and mitigate the intergenerational transmission of psychiatric disorders,[16] while lower quality parenting, interparental conflict, and socioeconomic deprivation have been shown to exacerbate children's developmental risk of emotional and behavioural problems.[11] In addition, being born preterm (i.e. <37 weeks' gestation) has been associated with alterations in early brain development,[17] as well as neurological, behavioural and cognitive problems in childhood and beyond.[18,19] Therefore, it is complex to disentangle the possible effects of postnatal maternal mental health and those of perinatal clinical factors on specific outcomes in preterm children, as these may involve both maternal psychosocial and biological variables, as well as child preterm-related neurodevelopmental morbidity.

For peer review only - http://bmjopen.bmj.com/site/about/guidelines.xhtml

Furthermore, a question that remains unanswered is whether preterm birth accentuates the association between maternal postnatal depression and child outcome. Two theoretical frameworks exist that hypothesise certain infants may be influenced differently by external stimuli: the diathesis stress model proposes that certain vulnerability factors make affected infants more prone to suboptimal environmental influences with subsequent poorer outcomes,[20,21] whereas the differential susceptibility model frames such factors as plasticity-mediating, thus leading to poorer outcomes in negative environments, as well as better outcomes in supportive environments.[21,22] Previous studies investigating differential susceptibility have shown mixed findings studying a range of environmental and clinical exposures,[24,27] with child outcomes including attachment, internalising and externalising behaviour, and academic competence.[27] Both low birthweight in term infants (small for gestational age) [23] and preterm birth [22,24,25] have been explored as distinct potential susceptibility factors, as the pathophysiological mechanisms underlying their respective differential susceptibility effects may differ.[26]

Given that mothers of preterm children experience elevated levels of distress,[28] are at high risk of developing postnatal depression,[29] and that preterm children themselves are vulnerable to psychiatric sequelae,[30] we aimed to investigate the association between very early symptoms of maternal postnatal depression and child behavioural and emotional outcomes, including ASD symptoms. No studies to date have investigated the interactive effect of preterm birth and maternal depression on outcomes, hence we also aimed to explore whether maternal depression had a differential effect on term and preterm children's behavioural outcomes. We specifically aimed to investigate the continuum of maternal depressive symptoms rather than solely focussing on clinically significant maternal depression, so as to provide more nuanced information about the importance of subclinical depressive symptoms on child outcomes. We hypothesised that early postnatal maternal depressive symptoms would be more elevated in mothers of preterm compared to term infants and that these would impact preterm children's behavioural and emotional outcomes to a greater degree than their term counterparts.

Methods

Sample

Participants were enrolled in the Developing Human Connectome Project (DHCP, http://www.developingconnectome.org/), a neuroimaging-focused project, with eligibility

For peer review only - http://bmjopen.bmj.com/site/about/guidelines.xhtml

criteria including pregnant women (aged ≥16 years) with a gestational age of 20–42 weeks, and newborn infants aged 24–44 weeks; infants enrolled in the DHCP had magnetic resonance imaging (MRI) at term-equivalent age. Exclusion criteria for the DHCP included: contraindications to MRI, babies being too unwell to tolerate a scan, and language difficulties preventing informed consent.[31] Toddlers were invited to the Centre for the Developing Brain, St Thomas' Hospital, London, for neurodevelopmental assessment at 18 months post-expected delivery date; appointments were made according to family availability as close as possible to this time-point. Inclusion criteria for our follow-up study were: mother and baby attendance for MRI at term-equivalent age; completed toddler neurodevelopmental assessment. These inclusion criteria were met by 509 toddlers by the date of closure for this analysis (26/02/2020). Of the 509 toddlers, 51 were one of a twin pregnancy, and three were one of a triplet pregnancy; the sample contained 22 sibling pairs and one set of triplets. This study was approved by the UK National Research Ethics Authority (14/LO/1169) and conducted in accordance with the World Medical Association's Code of Ethics (Declaration of Helsinki). Written informed consent was given by children's carer(s) at recruitment into the study.

Maternal variables

Maternal age, parity, Body Mass Index (BMI) and postcode were collected at enrolment into the DHCP study. Parity was coded as 0, 1, 2, or ≥3 previous children. Index of Multiple Deprivation (IMD) rank was computed from the current maternal postcode using the 2019 IMD classification; it combines locality-specific information about income, employment, education, health, crime, housing and living environment, thus providing a proxy for family socioeconomic status.[32] Lower IMD rank corresponds to greater social deprivation.

Maternal depressive symptoms were measured using the Edinburgh Postnatal Depression Scale (EPDS)[33] on the day of infant's MRI at term-equivalent age. Mothers of infants born at term were tested in the first few weeks postnatally, whereas mothers of preterm-born infants were tested once they reached term-corrected age. The EPDS is a 10-item screening questionnaire completed by mothers, with higher scores reflecting a higher likelihood of depressive disorders. A score of 13 can be used as a cut-off indicating high-level symptoms, although a cut-off of 11 maximises the sensitivity and specificity of the screening tool for depression.[34] Mothers completed the EPDS independently in a private room in our Centre, with no interaction with the researcher. Participants were informed that the results would be

For peer review only - http://bmjopen.bmj.com/site/about/guidelines.xhtml

discussed with them, and consented to information being shared with their General Practitioner in the case of high scores. The EPDS questionnaire was scored by a member of the DHCP team.[31]

Child variables

Infant *clinical characteristics* were gathered from clinical notes where available, or from maternal report, and included: sex, gestational age at birth, birth weight, and pregnancy size (singleton/twin/triplet).

Behavioural outcomes were assessed using the Child Behaviour Checklist/$1^{1/2}$-5 (CBCL), a parent-completed 100-item questionnaire, in which the parent rates the child's behaviour over the preceding two months using a 3-point Likert scale ("not true", "somewhat or sometimes true", and "very true or often true"). Responses are categorised into syndrome profiles, and these are subsequently grouped into internalising (emotional reactivity, anxiety/depression, somatic complaints, and withdrawal), externalising (attention problems, aggressive behaviour) and total (internalising, externalising, sleep and other) problem scales. Higher scores indicate increased emotional and behavioural problems. Total scores are classified into a normal range (<83rd centile, T <60), borderline range (83rd-90th centile, T 60-63), and clinical range (>90th centile, T ≥64).[35] The CBCL is known to have high reliability, validity and cross-informant agreement for measuring children's emotional and behavioural problems.[35]

ASD traits were assessed using the Quantitative Checklist for Autism in Toddlers (Q-CHAT). The Q-CHAT is a parent-completed 25-item questionnaire, in which the child's behaviour is scored on a 5-point (0-4) frequency scale. Higher total scores correspond to a higher frequency of autistic traits. The Q-CHAT shows good test-retest reliability, face validity and specificity, yet poor positive predictive value.[36,37]

Cognitive assessment was performed using the Bayley Scales of Infant and Toddler Development – Third Edition (Bayley-III). The Bayley-III provides scores for a child's overall cognitive, language and motor development. The cognitive standardised composite score was used in this study; scores between 70-84 indicate mild cognitive impairment, scores between 55-69 indicate moderate impairment, and scores lower than 55 indicate severe

For peer review only - http://bmjopen.bmj.com/site/about/guidelines.xhtml

impairment[38]. Reliability and validity of the Bayley-III have been shown to be robust,[39] although some studies report its underestimation of developmental problems.[40]

Assessments were carried out by staff experienced in the neurocognitive assessments of toddlers.

Analysis

Descriptive statistics and one-way ANOVA tests were performed in IBM SPSS Statistics for Windows v.25. All other analyses were carried out in Stata v.16.

Multiple imputation (MI) was carried out to account for missing data in CBCL (11/509, 2.24%), Q-CHAT (9/509, 1.8%), maternal EPDS (73/509, 14.3%), maternal BMI (27/509, 5.3%) and IMD rank (3/509, 0.6%). Variables were imputed simultaneously using the 'mi impute chained' procedure that performs imputation by chained equations. The imputation models had the same structural form as the analysis models, and included all variables that appear in the corresponding analysis models (maternal EPDS, maternal BMI, multiple pregnancy, parity, IMD rank, gestational age at birth, birthweight, sex, corrected age at assessment, and Bayley III Cognitive Composite score); in addition, maternal age was also included in the imputation model, as this predicted the incomplete variable and the probability of a value being observed.[41] To assess whether maternal age was predicting the probability of a value being observed, we firstly constructed binary indicators, one for each incomplete variable, that denoted whether the incomplete variable was missing their value (coded 0) or not (coded 1). These indicators then formed the dependent variable in logistic regression models that used maternal age as the independent variable. We used a 5% level to define a significant association.

Maternal EPDS and CBCL were imputed using Poisson regression; Q-CHAT, maternal BMI, and the IMD rank were imputed using linear regression. 40 MI datasets were created. To assess the stability of our MI parameters, we extracted the Monte Carlo error of each parameter estimate and examined whether the error for the coefficient was less than 10% of the parameter's standard error estimate. MI estimates were used for the primary analyses and compared to the estimates from complete-case (CC, individuals who had no missing data pre-imputation) analyses. Normal probability plots of residuals from the CC analyses were examined.

For peer review only - http://bmjopen.bmj.com/site/about/guidelines.xhtml

The analysis models were multiple linear regressions fitted using the 'mi estimate' procedure, which estimates effects after application of Rubin's rules.[42] To account for the small amount of clustering in our data (twin/triplet siblings), the models' standard errors were obtained using Stata's robust cluster estimator 'vce(cluster *idvar*)'. For continuous variables, Cohen's f-squared effect sizes were calculated using $f^2 = (R^2_{AB} - R^2_A)/(1 - R^2_{AB})$, where R^2_{AB} is the R-squared value from a regression model that includes the variable of interest as well as all the covariates used in the model, and R^2_A is the R-squared value from the regression model that includes only the covariates.[43,44] For binary variables, Cohen's f-squared effect sizes were produced after estimating first the Cohen's d using the formula: $f^2 = \frac{d}{2k}$, where k is the number of groups. As a measure of dispersion, Cohen's d used the average root mean-square error over the MI datasets. Adjusted R-squared values after MI were extracted after estimating the model with the user-written 'mibeta' command with the 'fisherz' option,[45] which calculates R-squared measures for linear regression with MI data. The significance of the joint effect of the categorical variable parity was assessed using `mi test' which performs Wald tests of composite linear hypotheses.

Primary outcome measures were children's total CBCL raw score and Q-CHAT score. S*econdary outcome measures* were CBCL internalising and externalising scores. The effect of maternal EPDS score was adjusted for IMD rank, maternal age, maternal BMI, maternal parity, pregnancy size, and the following child's variables: continuous gestational age, birth weight, Bayley-III cognitive composite score, and corrected age at assessment. The interaction between preterm birth and maternal depressive symptoms was explored using a complete case analysis in both CBCL and Q-CHAT models, using a dichotomised measure of gestational age. EPDS, CBCL and Q-CHAT scores were compared between term (≥37 weeks gestation) and preterm infants (<37 weeks gestation) using the complete case dataset.

In order to investigate the specificity of the association between maternal EPDS scores and child's behavioural outcomes (versus cognitive outcomes) we repeated the analyses to predict Bayley-III Cognitive Composite score, with the following variables: IMD rank, maternal age, maternal BMI, maternal parity, pregnancy size, and the following child's variables: gestational age, birth weight, corrected age at assessment, and Q-CHAT score. CBCL score

For peer review only - http://bmjopen.bmj.com/site/about/guidelines.xhtml

was not included in the model predicting cognitive outcome, because cognition was not a significant predictor of CBCL (see Results).

As all mothers had their EPDS score measured near term (or term-corrected in the case of mothers of preterm infants), we further investigated the association between time elapsing between baby's birth and mother's EPDS assessment and EPDS score, in order to avoid erroneously identifying 'baby blues' in mothers of term-born infants versus postnatal depression in mothers of preterm infants. This post-hoc analysis was performed using Poisson regression.

Patient and Public Involvement

The current study was developed in consultation with the Weston Programme for Family Centered Research, which involves parents to define what research is valuable to them, and to allow them to lead it with support from the scientists in the Centre for the Developing Brain.

Results

Descriptive statistics

Our sample of 509 toddlers were followed up at a median corrected age of 18.4 months (range 17.3 – 24.3 months). 51 (10.0%) of these were twins, and 3 (0.59%) were triplets. Of the 509, 21 (4.13%) mothers scored above a clinical cut-off (≥13) on the EPDS;(26) the distribution of maternal EPDS scores is shown graphically in Supplementary Figure 1. Demographic data are shown in Table 1. Complete data were available for 400 (78.6%) participants. Missing data were imputed and thus all 509 subjects were included in the primary and secondary analyses. One participant was excluded from the cognition analysis after examining the quintiles of the residuals against the theoretical quintiles of a normal distribution. The mean CBCL T score was 46.9 (SD 9.5) (Table 1); using CBCL-specified cut-offs,[35] 449 (90.2%) of participants had a CBCL score in the normal range, 30 (6.0%) were borderline, and 19 (3.8%) scored in the clinical range. The mean Q-CHAT score was 30.5 (SD 9.3) (Table 1). The mean Bayley III Cognitive Composite score in our sample was 100 (SD 11.4) (Table 1), which corresponds to the standardised test mean;[38] 480 (94.3%) of participants had a normal cognitive score, 24 (4.7%) had mild impairment, 5 (1%) had moderate impairment, and nil had severe impairment. This distribution is not dissimilar from that of the normative sample.[38]

For peer review only - http://bmjopen.bmj.com/site/about/guidelines.xhtml

Association between maternal EPDS score and toddler CBCL and Q-CHAT scores

Predictors of children's CBCL and Q-CHAT scores after multiple imputation are shown in Table 2. Higher maternal EPDS score was associated with children's higher CBCL Total score (B=0.93, 95% CI 0.43-1.44, $p<0.001$, $f^2=0.05$) and Q-CHAT score (B=0.27, 95% CI 0.03-0.52, p=.031, $f^2=0.01$) (Table 2). These associations are presented graphically in Figure 1 and Figure 2, respectively. Boys had higher CBCL and Q-CHAT scores than girls. Higher Q-CHAT scores were associated with lower IMD rank (i.e., greater socio-economic deprivation) and lower Bayley-III cognitive composite scores. Parity was not a significant predictor of outcome in any of the models (Table 2).

Association between maternal EPDS score and toddler CBCL internalising and externalising scores

Higher maternal EPDS score was associated with both internalising (B=0.22, 95% CI 0.08-0.36, $p<0.01$, $f^2=0.03$) and externalising (B=0.40, 95% CI 0.20-0.61, $p<0.001$, $f^2=0.05$) symptoms in children (Supplementary Table 1 and 2, respectively). Comparison of the imputed model analyses to the complete-case analyses showed that results were consistent for the CBCL model (Supplementary Table 3). Comparison for the Q-CHAT model showed that maternal EPDS was a significant predictor in the imputed model, but not in the complete-case analysis (Supplementary Table 3).

Interaction effect of preterm birth and maternal EPDS score on toddler CBCL and Q-CHAT scores

Maternal EPDS scores did not differ between preterm and term groups in the complete dataset ($t(434)=0.11$, p=0.92). CBCL scores ($t(496)=0.95$, p=0.34) and Q-CHAT scores ($t(122.6)=0.50$, p=0.62) did not differ between preterm and term groups in the complete dataset. Maternal EPDS score did not disproportionately affect preterm children with respect to CBCL or Q-CHAT scores (Table 3).

Association between maternal EPDS score and toddler cognitive outcomes

Predictors of children's cognitive score are shown in Table 4. Maternal EPDS score at term was not associated with toddlers' cognitive outcomes (B=-0.22, 95% CI -0.50-0.05, p=.108).

Effect of time-lag between baby's birth and mother's EPDS assessment and EPDS score

Mothers who gave birth prematurely (<37 weeks gestation) had their EPDS score assessed on average 7.7 weeks later post-delivery than mothers who gave birth at term (preterm

For peer review only - http://bmjopen.bmj.com/site/about/guidelines.xhtml

participants M=8.9 (SD 4.8), term participants M=1.2 (SD 1.3); $t(99.4)=15.5$, $p<.001$). The time-lag between birth and EPDS assessment did not predict maternal EPDS score, and there was no evidence of a significant interaction between gestation and birth-to-assessment time-lag (Supplementary Table 4 and 5, respectively).

Discussion

Principal findings

Our results showed that more maternal self-reported depressive symptoms shortly after birth were associated with greater parent-reported toddlers' behavioural problems – both internalising and externalising symptoms – and ASD traits, but not with cognitive outcomes. Given that fewer than 5% of the mothers in our cohort had EPDS scores above a clinical threshold,[33] our findings indicate that even subclinical depressive symptoms – i.e. not only diagnostic postnatal depression – adversely impact children's behavioural outcomes. In addition, our cohort was typically developing with few CBCL scores reaching a concerning threshold; our results could be interpreted within the conceptual framework of mental illness lying on a continuum with typical behavioural traits.[46] Our findings further showed that preterm birth did not influence the association between self-reported maternal depressive symptoms and parent-reported infants' behavioural outcomes in toddlerhood. This indicates that in this context preterm birth may not be regarded as a vulnerability or plasticity factor. Interestingly, mothers of preterm infants did not report more depressive symptoms compared to mothers of term infants in this study.

Comparison to prior literature

Our results with respect to internalising and externalising symptoms are in line with previous studies, including large population cohort studies, that showed an association between postnatal maternal depression and young children's emotional and behavioural problems.[11] Another previous study in 18-month old toddlers found that maternal depression was associated with internalising and dysregulated behaviour, but not externalising symptoms.[47] This difference between our and Conroy et al.'s findings may have arisen from their exclusion of infants born <36 weeks and their use of a clinical diagnosis of depression for mothers, rather than the continuous self-reported approach we employed. Interestingly, our finding that even subclinical depressive symptoms may adversely impact parent-reported child behavioural outcomes is in line with recent data showing that low- as well as high-level

depressive symptoms are associated with internalising and externalising symptoms in children aged 3 years.[48]

The results showing an association between maternal postnatal depressive symptoms and parental report of childhood ASD traits are less robust and need to be interpreted with caution. Although some prior studies have shown an association between antenatal maternal depression and offspring's ASD,[10,49] and postnatal depression has been suggested as a potential focus of cross-domain studies of ASD,[50] there is no clear aetiological role of maternal postnatal depression in the development of ASD *per se*. Also, given that mothers with ASD are more likely to suffer from perinatal depression than mothers without ASD,[51] and ASD is highly heritable,[52] maternal depression may actually be a confounding rather than causative factor in our observed results.

The finding that preterm infants were not disproportionately affected by maternal depressive symptoms supports Hadfield et al.'s findings that maternal distress at 9 months did not differentially impact very preterm (<34 weeks) or late preterm (34-36^{+6} weeks) infants with respect to socioemotional outcomes, although paternal distress did have an impact on very preterm infants' outcomes.[24] However, our results differ from Gueron-Sela et al.'s finding that very preterm (28-33 weeks) 12 month old infants' social outcomes were more influenced by maternal emotional distress at 6 months than term infants' outcomes.[22] The inconsistent findings may be due to methodological differences: for instance, our infant assessment being conducted at 18 months corrected age when social competency is more developed, our assessment of maternal depressive symptoms being in the very early postnatal period, or our use of a screening measure, the Q-CHAT, as a measure of ASD traits. Importantly, the lack of support for a diathesis-stress or differential susceptibility model of maternal mental state on preterm infants in our study must be viewed in the context of our results also showing no difference in CBCL and Q-CHAT scores between term and preterm infants. This is in contrast to the existing literature that preterm infants are more likely than term infants to develop behavioural problems, such as ADHD, in childhood and adolescence.[19,30] It is possible that the phenotypes of neurodevelopmental and neuropsychiatric disorders assessed with the chosen behavioural measures may not be sufficiently expressed at 18 months corrected age.[53] In addition, as briefly discussed above, much of the existing literature emphasises the risk of extreme (<28 weeks) or very preterm (28-33 weeks) birth on later

For peer review only - http://bmjopen.bmj.com/site/about/guidelines.xhtml

behavioural outcomes,[19,30] whereas only 3.5% and 5.5% of our participants fell within the extreme and very preterm group, respectively, and we thus may not have the power to show any subtle effects.

Strengths & limitations of the study

The strengths of this study lie primarily in its large sample and prospective data collection. Moreover, the use of multiple imputation methodology has facilitated retention of a complete dataset, thus minimising non-response bias and increasing parameter precision. A strength in comparison to prior population cohort studies is that we assessed very early maternal depressive symptoms, and our sample is perhaps more representative of today's society – with increasing maternal age – than large cohort studies conducted in the 1990s-2000s. Given the complex interplay of biological and environmental factors in the aetiology of behavioural disorders, the inclusion of a substantive proportion of preterm infants in our cohort also offers an important insight into the role of preterm birth in behavioural outcomes; moreover, our results represent the full gestational spectrum, rather than discrete gestational categories. In addition, using maternal depressive symptoms as a continuous, rather than dichotomous, variable allows a more nuanced understanding of the role maternal postnatal depressive symptoms may play in influencing children's outcomes.

There are several limitations to this study that necessitate our findings to be considered with caution. Firstly, differences in birth-to-EPDS-assessment time-lags are a potential confounder, given the time-sensitive nature of early-onset temporary baby blues and later-onset pathological postnatal depression. Mothers of infants born at term were assessed early post-delivery, within the period one would anticipate baby blues to present, whereas mothers of preterm participants were on average assessed later, when postnatal depression predominates.[1,54] Although our post-hoc analyses showed that the time elapsed from birth to EPDS assessment was not associated with maternal EPDS score, providing reassurance that our assessments of mothers of term-born infants were not inflated by the common temporary symptoms of baby blues, it is possible that we did not capture the full extent of later-onset depressive symptoms in mothers of term-born infants. This may explain why maternal EPDS scores did not differ between preterm and term groups in our complete dataset analysis, contrary to the current literature,[28] as well as why our rate of postpartum depression, using an EPDS cut-off of 13, was low (4.1%) compared to the previously documented UK community

For peer review only - http://bmjopen.bmj.com/site/about/guidelines.xhtml

prevalence rate of 8.9% at eight weeks postpartum.[55] Our results must therefore be interpreted with some caution.

Secondly, although statistical techniques were used to impute missing data and mitigate this problem, 14.3% of maternal EPDS scores were missing. This rate of missingness may relate to some mothers being reluctant to complete a questionnaire at the time their child is having an MRI, or due to simultaneous childcare duties. Thirdly, a number of important confounders that are likely to affect children's behavioural outcomes were not assessed in this study, including genetic risk for psychiatric disorders,[56] parental psychiatric co-morbidities,[47] chronicity of postnatal depressive symptoms,[48] antenatal maternal depression, paternal depression and subsequent parent-infant attachment, and inter-parental conflict.[11] Thus, we are unable to conclude whether our observed associations between early postnatal maternal depressive symptoms and children's behavioural outcomes are moderated or mediated by other parental and/or psychiatric factors.

Fourthly, whilst our study included a reasonable proportion of preterm infants (97/509, 19%), our sample was not random, as preterm children were selectively recruited for the DHCP; indeed, preterm infants are over-represented in our sample when compared to the UK population incidence (7.3%),[57] which may limit the study's generalisability to the general population. This over-representation of preterm infants may explain why our mean maternal age is higher than the national mean age of 30.7,[58] given that increasing maternal age is associated with increased risk of adverse pregnancy outcomes.[59] Furthermore, although a 19% prevalence of preterm birth is high for a community sample, the proportion of very and extreme preterm infants in our sample is small, and this may not have provided sufficient power to detect any differential susceptibility effect of preterm birth on outcomes.

Sixthly, the effect sizes of the association between maternal EPDS score and behavioural problems and ASD traits, respectively, were small; this raises questions regarding the clinical significance of our findings and potentially explains some of the inconsistency between this and previous studies. Even within our analyses, the association between maternal depressive symptoms and ASD traits was not observed in our complete case analysis, thus calling into question the validity of this result. It is also important to highlight the continuum of ASD traits that are conceptualised by the Q-CHAT,[36] as well as its poor positive predictive value;[37]

For peer review only - http://bmjopen.bmj.com/site/about/guidelines.xhtml

the presence of traits does not imply a diagnosis of ASD, and this distinction may also explain the contrast to previous studies.

Finally, it is well documented that maternal depression influences reporting of ASD traits,[60] Q-CHAT,[61] and CBCL scores.[62] Our study used maternal report of maternal depressive symptoms, and our outcome measures were parent-completed questionnaires; despite the CBCL showing good cross-informant agreement,[35] it is thus possible that reporting bias with common method variance could have skewed our results.

Implications of our findings

Of greatest importance to clinicians and policymakers is our finding that even *subclinical* self-reported maternal depressive symptoms are associated with parent-reported behavioural outcomes of offspring. This has significant implications for the risk-stratification of women and their babies in the postnatal period, during which contact with medical professionals is already established. Identifying high risk families and providing appropriate supportive measures at the early postnatal stage may help to prevent future psychiatric morbidity.

Future research

Further follow-up of large cohorts of preterm and term infants, to an age when behavioural phenotypes may become more pronounced, is needed to investigate whether the long-term developmental trajectories of term and ex-preterm infants are differentially susceptible to changes of postnatal maternal mental health. Future research should consider both maternal and paternal mental health, as well as socioeconomic and environmental factors on child outcomes. Such follow-up should use independent, objective assessments of child behavioural outcomes in order to avoid the common method variance inherent to parent-reported measures. In addition, further study is also needed to elucidate the role of maternal depression in the aetiology of ASD, controlling for both diagnostic and sub-clinical maternal ASD symptomatology. Finally, it is crucial for future research to elucidate the interplay of biochemical and neurodevelopmental changes that may mediate and confound the translation of environmental exposures into distal behavioural phenotypes.

Conclusion

This prospective longitudinal cohort study found no evidence to support the concept of preterm birth as a vulnerability or plasticity factor with respect to the effect of maternal

For peer review only - http://bmjopen.bmj.com/site/about/guidelines.xhtml

depressive symptoms on behavioural development. However, we showed that early subclinical maternal postnatal depressive symptoms were associated with behavioural problems in children on parent-reported measures. This adds to the increasing body of literature indicating the role of subclinical and early postnatal depressive symptoms in the aetiology of childhood behavioural disorders. These findings are of great relevance to child and public health, and further research may strengthen its implications for developing strategies to facilitate effective screening and support for women and children, enabling all to reach their full potential.

For peer review only

Acknowledgements & contributions

We thank all DHCP investigators for their contribution to the study. We thank Dr Oliver Gale-Grant MRes (Centre for the Developing Brain, King's College London; Department of Forensic & Neurodevelopmental Sciences, King's College London) for providing the IMD rank data. We are very grateful to the families who generously took part in this research.

Conceptualization: SC, ADE, CN; **Methodology:** IK, GV, SC, AP, ADE, CN; **Investigation:** SF, AC; **Data curation:** IK, AL; **Formal analysis:** IK, GV, AP, CN; **Writing – original draft preparation:** IK; **Writing – Review & Editing:** GV, AL, SF, AC, SC, AP, ADE, CN; **Visualisation:** IK, GV; **Funding acquisition:** SC, ADE; **Supervision:** AP, ADE, CN.

References

1. Woody CA, Ferrari AJ, Siskind DJ, Whiteford HA, Harris MG. A systematic review and meta-regression of the prevalence and incidence of perinatal depression. *J Affect Disord*. 2017;219:86-92. doi:10.1016/j.jad.2017.05.003

2. Rezaie-Keikhaie K, Arbabshastan ME, Rafiemanesh H, Amirshahi M, Ostadkelayeh SM, Arbabisarjou A. Systematic Review and Meta-Analysis of the Prevalence of the Maternity Blues in the Postpartum Period. *J Obstet Gynecol Neonatal Nurs*. 2020;49(2):127-136. doi:10.1016/j.jogn.2020.01.001

3. Beck CT. Predictors of postpartum depression: an update. *Nurs Res*. 2001;50(5):275-285.

4. de Paula Eduardo JAF, de Rezende MG, Menezes PR, Del-Ben CM. Preterm birth as a risk factor for postpartum depression: A systematic review and meta-analysis. *J Affect Disord*. 2019;259:392-403. doi:10.1016/j.jad.2019.08.069

For peer review only - http://bmjopen.bmj.com/site/about/guidelines.xhtml

5. Blom EA, Jansen PW, Verhulst FC, et al. Perinatal complications increase the risk of postpartum depression. The Generation R Study. *BJOG Int J Obstet Gynaecol*. 2010;117(11):1390-1398. doi:10.1111/j.1471-0528.2010.02660.x

6. Beck CT. Postpartum depression: a metasynthesis. *Qual Health Res*. 2002;12(4):453-472. doi:10.1177/104973202129120016

7. Grace SL, Evindar A, Stewart DE. The effect of postpartum depression on child cognitive development and behavior: a review and critical analysis of the literature. *Arch Womens Ment Health*. 2003;6(4):263-274. doi:10.1007/s00737-003-0024-6

8. Lebel C, Walton M, Letourneau N, Giesbrecht GF, Kaplan BJ, Dewey D. Prepartum and postpartum maternal depressive symptoms are related to children's brain structure in preschool. *Biol Psychiatry*. 2016;80(11):859-868. doi:10.1016/j.biopsych.2015.12.004

9. Goodman SH, Rouse MH, Connell AM, Broth MR, Hall CM, Heyward D. Maternal depression and child psychopathology: a meta-analytic review. *Clin Child Fam Psychol Rev*. 2011;14(1):1-27. doi:10.1007/s10567-010-0080-1

10. Hagberg KW, Robijn AL, Jick S. Maternal depression and antidepressant use during pregnancy and the risk of autism spectrum disorder in offspring. *Clin Epidemiol*. 2018;10:1599-1612. doi:10.2147/CLEP.S180618

11. Stein A, Pearson RM, Goodman SH, et al. Effects of perinatal mental disorders on the fetus and child. *The Lancet*. 2014;384(9956):1800-1819. doi:10.1016/S0140-6736(14)61277-0

12. Van Batenburg-Eddes T, Brion MJ, Henrichs J, et al. Parental depressive and anxiety symptoms during pregnancy and attention problems in children: a cross-cohort consistency study. *J Child Psychol Psychiatry*. 2013;54(5):591-600. doi:10.1111/jcpp.12023

13. Barker ED, Jaffee SR, Uher R, Maughan B. The contribution of prenatal and postnatal maternal anxiety and depression to child maladjustment. *Depress Anxiety*. 2011;28(8):696-702. doi:10.1002/da.20856

14. Pearson RM, Evans J, Kounali D, et al. Maternal depression during pregnancy and the postnatal period: risks and possible mechanisms for offspring depression at 18 years. *JAMA Psychiatry*. 2013;70(12):1312-1319. doi:10.1001/jamapsychiatry.2013.2163

15. Kleine I, Falconer S, Roth S, et al. Early postnatal maternal trait anxiety is associated with the behavioural outcomes of children born preterm <33 weeks. *J Psychiatr Res*. 2020;131:160-168. doi:10.1016/j.jpsychires.2020.09.010

16. Feder A, Nestler EJ, Charney DS. Psychobiology and molecular genetics of resilience. *Nat Rev Neurosci*. 2009;10(6):446-457. doi:10.1038/nrn2649

17. Dimitrova R, Pietsch M, Christiaens D, et al. Heterogeneity in brain microstructural development following preterm birth. *Cereb Cortex*. 2020;30(9):4800-4810. doi:10.1093/cercor/bhaa069

For peer review only

For peer review only - http://bmjopen.bmj.com/site/about/guidelines.xhtml

18. Fitzallen GC, Sagar YK, Taylor HG, Bora S. Anxiety and depressive disorders in children born preterm: a meta-analysis. *J Dev Behav Pediatr JDBP*. 2021;42(2):154-162. doi:10.1097/DBP.0000000000000898

19. Allotey J, Zamora J, Cheong-See F, et al. Cognitive, motor, behavioural and academic performances of children born preterm: a meta-analysis and systematic review involving 64 061 children. *BJOG Int J Obstet Gynaecol*. 2018;125(1):16-25. doi:10.1111/1471-0528.14832

20. Jaekel J, Pluess M, Belsky J, Wolke D. Effects of maternal sensitivity on low birth weight children's academic achievement: a test of differential susceptibility versus diathesis stress. *J Child Psychol Psychiatry*. 2015;56(6):693-701. doi:10.1111/jcpp.12331

21. Belsky J, Bakermans-Kranenburg MJ, van IJzendoorn MH. For better and for worse: differential susceptibility to environmental influences. *Curr Dir Psychol Sci*. 2007;16(6):300-304. doi:10.1111/j.1467-8721.2007.00525.x

22. Gueron-Sela N, Atzaba-Poria N, Meiri G, Marks K. The caregiving environment and developmental outcomes of preterm infants: diathesis stress or differential susceptibility effects? *Child Dev*. 2015;86(4):1014-1030. doi:https://doi.org/10.1111/cdev.12359

23. Hadfield K, O'Brien F, Gerow A. Is level of prematurity a risk/plasticity factor at three years of age? *Infant Behav Dev*. 2017;47:27-39. doi:10.1016/j.infbeh.2017.03.003

24. Belsky J, Pluess M. Beyond diathesis stress: differential susceptibility to environmental influences. *Psychol Bull*. 2009;135(6):885-908. doi:10.1037/a0017376

25. Pluess M, Belsky J. Prenatal programming of postnatal plasticity? *Dev Psychopathol*. 2011;23(1):29-38. doi:10.1017/S0954579410000623

26. DeMaster D, Bick J, Johnson U, Montroy JJ, Landry S, Duncan AF. Nurturing the preterm infant brain: leveraging neuroplasticity to improve neurobehavioral outcomes. *Pediatr Res*. 2019;85(2):166-175. doi:10.1038/s41390-018-0203-9

27. World Health Organization (WHO). Global nutrition targets 2025: low birth weight policy brief. Published online 2014.

28. Carson C, Redshaw M, Gray R, Quigley MA. Risk of psychological distress in parents of preterm children in the first year: evidence from the UK Millennium Cohort Study. *BMJ Open*. 2015;5(12):e007942. doi:10.1136/bmjopen-2015-007942

29. Vigod SN, Villegas L, Dennis CL, Ross LE. Prevalence and risk factors for postpartum depression among women with preterm and low-birth-weight infants: a systematic review. *BJOG Int J Obstet Gynaecol*. 2010;117(5):540-550. doi:10.1111/j.1471-0528.2009.02493.x

30. Johnson S, Wolke D. Behavioural outcomes and psychopathology during adolescence. *Early Hum Dev*. 2013;89(4):199-207. doi:10.1016/j.earlhumdev.2013.01.014

31. Lautarescu A, Victor S, Lau-Zhu A, Counsell SJ, Edwards AD, Craig MC. The factor structure of the Edinburgh Postnatal Depression Scale among perinatal high-risk and

For peer review only - http://bmjopen.bmj.com/site/about/guidelines.xhtml

community samples in London. *Arch Womens Ment Health*. 2022;25(1):157-169. doi:10.1007/s00737-021-01153-0

32. Ministry of Housing, Communities & Local Government. The English indices of deprivation 2019. Published online 2019. https://assets.publishing.service.gov.uk/government/uploads/system/uploads/attachment_data/file/835115/IoD2019_Statistical_Release.pdf

33. Cox JL, Holden JM, Sagovsky R. Detection of postnatal depression: development of the 10-item Edinburgh Postnatal Depression Scale. *Br J Psychiatry*. 1987;150(6):782-786. doi:10.1192/bjp.150.6.782

34. Levis B, Negeri Z, Sun Y, Benedetti A, Thombs BD. Accuracy of the Edinburgh Postnatal Depression Scale (EPDS) for screening to detect major depression among pregnant and postpartum women: systematic review and meta-analysis of individual participant data. *BMJ*. 2020;371. doi:10.1136/bmj.m4022

35. Achenbach TM, Rescorla LA. *Manual for the ASEBA preschool forms & profiles.*; 2000.

36. Allison C, Baron-Cohen S, Wheelwright S, et al. The Q-CHAT (Quantitative Checklist for Autism in Toddlers): a normally distributed quantitative measure of autistic traits at 18-24 months of age: preliminary report. *J Autism Dev Disord*. 2008;38(8):1414-1425. doi:10.1007/s10803-007-0509-7

37. Allison C, Matthews FE, Ruta L, et al. Quantitative Checklist for Autism in Toddlers (Q-CHAT). A population screening study with follow-up: the case for multiple time-point screening for autism. *BMJ Paediatr Open*. 2021;5(1):e000700. doi:10.1136/bmjpo-2020-000700

38. Bayley N. *Bayley scales of infant and toddler development - third edition*. 3rd ed. Harcourt Assessment; 2006.

39. Albers CA, Grieve AJ. Review of Bayley scales of infant and toddler development-third edition. *J Psychoeduc Assess*. 2007;25(2):180-190. doi:10.1177/0734282906297199

40. Anderson PJ, De Luca CR, Hutchinson E, Roberts G, Doyle LW, the Victorian Infant Collaborative Group. Underestimation of developmental delay by the new Bayley-III scale. *Arch Pediatr Adolesc Med*. 2010;164(4):352-356. doi:10.1001/archpediatrics.2010.20

41. White IR, Royston P, Wood AM. Multiple imputation using chained equations: Issues and guidance for practice. *Stat Med*. 2011;30(4):377-399. doi:https://doi.org/10.1002/sim.4067

42. Rubin DB. Inference and missing data. *Biometrika*. 1976;63(3):581-592. doi:10.2307/2335739

43. Cohen J. *Statistical power analysis for the behavioral sciences*. 2nd ed. L. Erlbaum Associates; 1988.

For peer review only

44. Selya AS, Rose JS, Dierker LC, Hedeker D, Mermelstein RJ. A practical guide to calculating Cohen's f2, a measure of local effect size, from PROC MIXED. *Front Psychol*. 2012;3. doi:10.3389/fpsyg.2012.00111

45. Harel O. The estimation of R 2 and adjusted R 2 in incomplete data sets using multiple imputation. *J Appl Stat*. 2009;36(10):1109-1118. doi:10.1080/02664760802553000

46. Plomin R, Haworth CMA, Davis OSP. Common disorders are quantitative traits. *Nat Rev Genet*. 2009;10(12):872-878. doi:10.1038/nrg2670

47. Conroy S, Pariante CM, Marks MN, et al. Maternal psychopathology and infant development at 18 months: the impact of maternal personality disorder and depression. *J Am Acad Child Adolesc Psychiatry*. 2012;51(1):51-61. doi:10.1016/j.jaac.2011.10.007

48. Kingston D, Kehler H, Austin MP, et al. Trajectories of maternal depressive symptoms during pregnancy and the first 12 months postpartum and child externalizing and internalizing behavior at three years. *PloS One*. 2018;13(4):e0195365. doi:10.1371/journal.pone.0195365

49. Wiggins LD, Rubenstein E, Daniels J, et al. A phenotype of childhood autism is associated with preexisting maternal anxiety and depression. *J Abnorm Child Psychol*. 2019;47(4):731-740. doi:10.1007/s10802-018-0469-8

50. Babb JA, Deligiannidis KM, Murgatroyd CA, Nephew BC. Peripartum depression and anxiety as an integrative cross-domain target for psychiatric preventative measures. *Behav Brain Res*. 2015;0:32-44. doi:10.1016/j.bbr.2014.03.039

51. Pohl AL, Crockford SK, Blakemore M, Allison C, Baron-Cohen S. A comparative study of autistic and non-autistic women's experience of motherhood. *Mol Autism*. 2020;11(1):3. doi:10.1186/s13229-019-0304-2

52. Colvert E, Tick B, McEwen F, et al. Heritability of autism spectrum disorder in a UK population-based twin sample. *JAMA Psychiatry*. 2015;72(5):415-423. doi:10.1001/jamapsychiatry.2014.3028

53. Egger HL, Emde RN. Developmentally sensitive diagnostic criteria for mental health disorders in early childhood: the diagnostic and statistical manual of mental disorders-IV, the research diagnostic criteria-preschool age, and the diagnostic classification of mental health and developmental disorders of infancy and early childhood-revised. *Am Psychol*. 2011;66(2):95-106. doi:10.1037/a0021026

54. Henshaw C. Mood disturbance in the early puerperium: a review. *Arch Women's Ment Health*. 2003;6(2):s33-s42. doi:10.1007/s00737-003-0004-x

55. Heron J, O'Connor TG, Evans J, Golding J, Glover V. The course of anxiety and depression through pregnancy and the postpartum in a community sample. *J Affect Disord*. 2004;80(1):65-73. doi:10.1016/j.jad.2003.08.004

56. Biel MG. Parental psychiatric symptoms and children's outcomes: toward understanding and responding to intergenerational risk in child psychiatry. *J Am Acad Child Adolesc Psychiatry*. 2018;57(9):632-633. doi:10.1016/j.jaac.2018.06.010

For peer review only - http://bmjopen.bmj.com/site/about/guidelines.xhtml

57. National Institute for Health and Care Excellence. Preterm labour and birth (NICE guideline 25). Published online November 2015. Accessed January 27, 2019. https://www.nice.org.uk/guidance/ng25

58. Office for National Statistics. Birth characteristics in England and Wales: 2020. Published January 13, 2022. Accessed February 5, 2022. https://www.ons.gov.uk/peoplepopulationandcommunity/birthsdeathsandmarriages/livebirths/bulletins/birthcharacteristicsinenglandandwales/2020#birth-characteristics

59. Londero AP, Rossetti E, Pittini C, Cagnacci A, Driul L. Maternal age and the risk of adverse pregnancy outcomes: a retrospective cohort study. *BMC Pregnancy Childbirth*. 2019;19(1):261. doi:10.1186/s12884-019-2400-x

60. Bennett T, Boyle M, Georgiades K, et al. Influence of reporting effects on the association between maternal depression and child autism spectrum disorder behaviors. *J Child Psychol Psychiatry*. 2012;53(1):89-96. doi:10.1111/j.1469-7610.2011.02451.x

61. Goh DA, Gan D, Kung J, et al. Child, maternal and demographic factors influencing caregiver-reported autistic trait symptomatology in toddlers. *J Autism Dev Disord*. 2018;48(4):1325-1337. doi:10.1007/s10803-018-3471-7

62. Friedlander S, Weiss DS, Traylor J. Assessing the influence of maternal depression on the validity of the Child Behavior Checklist. *J Abnorm Child Psychol*. 1986;14(1):123-133. doi:10.1007/bf00917228

For peer review only

For peer review only - http://bmjopen.bmj.com/site/about/guidelines.xhtml

Table 1: Socio-demographic, maternal and clinical characteristics (n=509)

Variable	Number (%)*
Sex: Male	274 (53.8)
Index of Multiple Deprivation (IMD) quintiles	
1 (least deprived) [a]	65 (12.8)
2	87 (17.2)
3	108 (21.3)
4	173 (34.2)
5 (most deprived)	73 (14.4)
Gestational age at birth (weeks), median [range]	39.7 [20 – 43]
Gestational category	
Extremely preterm (<28 weeks)	18 (3.5)
Very preterm (28-32 weeks)	28 (5.5)
Late preterm (32-37 weeks)	51 (10.0)
Term (≥37 weeks)	412 (80.9)
Birthweight (g), median [range]	3290 [450 – 4750]
Multiple pregnancy	54 (10.6)
Maternal parity	
0	332 (65.2)
1	124 (24.4)
2	32 (6.3)
3+	21 (4.2)
Maternal BMI (kg/m^2), median [range]	23.2 [15.3 – 43.6]
Maternal age at infant's birth (years), mean (SD) [range]	34.2 (4.8) [17 – 52]
Maternal ethnicity	
White	272 (53.4)
Black/Black British	56 (11.0)
Asian/Asian British	28 (5.5)
Chinese	18 (3.5)
Mixed – White & Asian	4 (0.8)
Mixed – White & Black	4 (0.8)
Any other	30 (5.9)
Do not wish to answer	9 (1.8)
No data	88 (17.3)
Bayley III cognitive composite score, mean (SD) [range]	100 (11.4) [55 – 125]
CBCL total T score, mean (SD) [range]	46.9 (9.5) [28 – 69]
Q-CHAT total score, mean (SD) [range]	30.5 (9.3) [8 – 70]
EPDS score, median [range]	4 [0 – 28]
EPDS score, n (%)	
<13	415 (8.2)
≥13	21 (4.1)
No data	73 (14.3)

[a] Quintile 1 corresponds to the highest, least deprived, IMD rankings.

* unless otherwise specified

For peer review only - http://bmjopen.bmj.com/site/about/guidelines.xhtml

Table 2: CBCL and Q-CHAT model predictors using multiple imputation without interaction. (Cf. Supplementary Table 3 for complete case analysis)

	CBCL			Q-CHAT		
	**B [95%CI]**	**p**	**f^2**	**B [95%CI]**	**p**	**f^2**
**Maternal EPDS**	0.93 [0.43, 1.44]	<.001 ***	0.05	0.27 [0.03, 0.52]	.031 *	0.01
**Maternal BMI**	-0.09 [-0.44, 0.26]	.621	-	0.06 [-0.13, 0.24]	.538	-
**Multiple pregnancy**	3.15 [-3.07, 9.37]	.320	-	1.33 [-2.62, 5.28]	.509	-
**Parity**						
**1**	-2.52 [-5.96, 0.93]	.151	-	-2.14 [-4.02, -0.27]	.025 [a]	-
**2**	-3.23 [-9.16, 2.70]	.285	-	0.88 [-1.99, 3.75]	.548	-
**3+**	-1.37 [-8.36, 5.61]	.699	-	-0.49 [-4.57, 3.60]	.815	-
**IMD rank**	-1.48 [-3.33, 0.37]	.117	-	-1.50 [-2.60, -0.40]	.008 **	0.02
**Gestational age at birth (weeks)**	0.10 [-0.65, 0.85]	.786	-	0.26 [-0.17, 0.70]	.233	-
**Birthweight (kg)**	0.56 [-2.65, 3.78]	.731	-	-1.24 [-2.93, 0.46]	.151	-
**Sex:female**	-4.14 [-6.96, -1.31]	.004 **	0.06	-1.95 [-3.42, -0.48]	.009 **	0.05
**Corrected age at assessment (months)**	-0.90 [-2.17, 0.37]	.166	-	-0.16 [-0.91, 0.59]	.677	-
**Cognition**	-0.05 [-0.20, 0.09]	.467	-	-0.27 [-0.35, -0.20]	<.001 ***	0.12

$p<0.05$ *; $p<0.01$ **; $p<0.001$ ***
CBCL model adjusted $R^2 = 0.0676$. Q-CHAT model adjusted $R^2 = 0.193$.
B = unstandardised coefficient.
CBCL = Child Behaviour Checklist score at 18 months. Q-CHAT = Quantitative Checklist for Autism in Toddlers score at 18 months. Maternal EPDS = maternal Edinburgh Postnatal Depression Scale score at term-equivalent age. Multiple pregnancy = dummy variable of twin/triplet pregnancy. Parity = dummy variable, one/two/three+ previous child(ren). Corrected age at assessment (months) = age at behavioural assessment, corrected for gestational age. Cognition = infant Bayley III score at 18 months.
[a] Wald test of whole parity variable in Q-CHAT model: $F(3, 476.9) = 1.88$, $p = .133$
Effect size (Cohen's f^2, calculated from squared part correlations for predictors significant to 0.05): 0.02 = small, 0.15 = medium and 0.35 = large.[43]
- indicates data not given, as predictor not significant to 0.05.

For peer review only - http://bmjopen.bmj.com/site/about/guidelines.xhtml

Table 3: CBCL and Q-CHAT model predictors using complete case analysis with interaction of 'EPDS x term'.

	CBCL		Q-CHAT	
	B [95%CI]	p	B [95%CI]	p
**Maternal EPDS**	0.89 [-0.24, 2.02]	.121	0.24 [-0.28, 0.75]	.365
**Maternal BMI**	-0.01 [-0.39, 0.37]	.955	0.00 [-0.16, 0.17]	.982
**Multiple pregnancy**	1.76 [-6.65, 10.17]	.681	0.97 [-2.07, 4.01]	.532
**Parity**				
**1**	-2.75 [-6.49, 0.99]	.149	-1.42 [-3.30, 0.46]	.139
**2**	-3.49 [-10.36, 3.37]	.317	0.16 [-2.84, 3.16]	.917
**3+**	-1.17 [-9.69, 7.35]	.788	-1.13 [-4.24, 1.98]	.476
**IMD rank**	-1.41 [-3.54, 0.73]	.195	-1.68 [-2.64, -0.72]	.001 **
**Gestation: term**	1.25 [-8.34, 10.85]	.797	2.64 [-1.74, 7.02]	.236
**Birthweight (kg)**	-1.01 [-4.08, 2.05]	.516	-2.25 [-3.73, -0.78]	.003 **
**Sex: female**	-4.64 [-7.83, -1.44]	.005 **	-2.22 [-3.72, -0.71]	.004 **
**Corrected age at assessment (months)**	-0.83 [-2.27, 0.62]	.261	-0.39 [-1.18, 0.04]	.335
**Cognition**	-0.03 [-0.20, 0.14]	.720	-0.22 [-0.29, -.0.15]	<.001 ***
**EPDS x gestation:term**	-0.01 [-1.30, 1.28]	.991	-0.02 [-0.60, 0.56]	.950

$p<0.05$ *; $p<0.01$ **; $p<0.001$ ***
CBCL model adjusted $R^2 = 0.0865$. Q-CHAT model adjusted $R^2 = 0.215$.
B = unstandardised coefficient.
CBCL = Child Behaviour Checklist score at 18 months. Q-CHAT = Quantitative Checklist for Autism in Toddlers score at 18 months. Maternal EPDS = maternal Edinburgh Postnatal Depression Scale score at term-equivalent age. Multiple pregnancy = dummy variable of twin/triplet pregnancy. Parity = dummy variable, one/two/three+ previous child(ren). Gestation: term = dummy variable, term (≥37 weeks) vs preterm (<37 weeks) gestation at birth. Corrected age at assessment (months) = age at behavioural assessment, corrected for gestational age. Cognition = infant Bayley III score at 18 months. EPDS x gestation:term = interaction term between maternal EPDS score and term gestation at birth.

For peer review only

Table 4: Cognition model predictors using multiple imputation.

	B [95%CI]	p
**Maternal EPDS**	-0.22 [-0.50, 0.05]	.108
**Maternal BMI**	-0.32 [-0.52, -0.13]	.001 **
**Multiple pregnancy**	1.65 [-2.49, 5.79]	.433
**Parity**		
**1**	-0.46 [-2.67, 1.76]	.686
**2**	-3.47 [-6.69, -0.25]	.035 [a]
**3+**	-4.57 [-9.53, 0.40]	.072
**IMD rank**	1.43 [0.37, 2.50]	.009 **
**Gestational age at birth (weeks)**	0.45 [-0.07, 0.96]	.091
**Birthweight (kg)**	0.81 [-1.24, 2.87]	.436
**Sex: female**	1.99 [0.24, 3.74]	.026 *
**Corrected age at assessment (months)**	-0.75 [-1.59, 0.08]	.075
**Q-CHAT score**	-0.39 [-0.50, -0.28]	<.001 ***

$p<0.05$ *; $p<0.01$ **; $p<0.001$ ***
Adjusted $R^2 = 0.231$
B = unstandardised coefficient.
Maternal EPDS = maternal Edinburgh Postnatal Depression Scale score at term-equivalent age. Multiple pregnancy = dummy variable of twin/triplet pregnancy. Parity = dummy variable, one/two/three+ previous child(ren). Corrected age at assessment (months) = age at behavioural assessment, corrected for gestational age. Cognition = infant Bayley III score at 18 months. Q-CHAT score = infant's Q-CHAT score at 18 month assessment.
[a] Wald test of whole parity variable in cognition model: $F(3, 482.9)=2.41$, $p=0.067$

Fig.1 Children's predicted CBCL scores at 18 months are positively correlated to the maternal EPDS score at term-equivalent age.

Fig.2 Children's predicted Q-CHAT scores at 18 months are positively correlated to the maternal EPDS score at term-equivalent age.

For peer review only

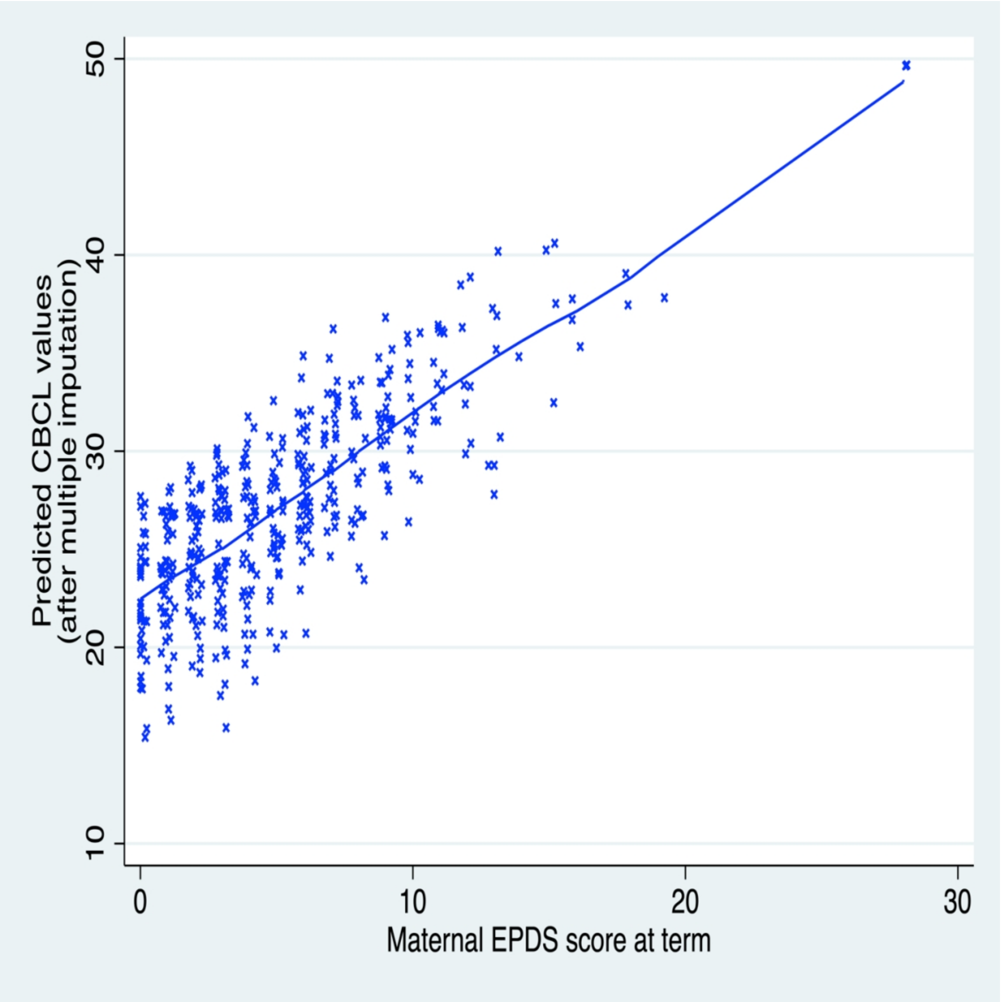

Fig.1 Children's predicted CBCL scores at 18 months are positively correlated to the maternal EPDS score at term-equivalent age

158x158mm (220 x 220 DPI)

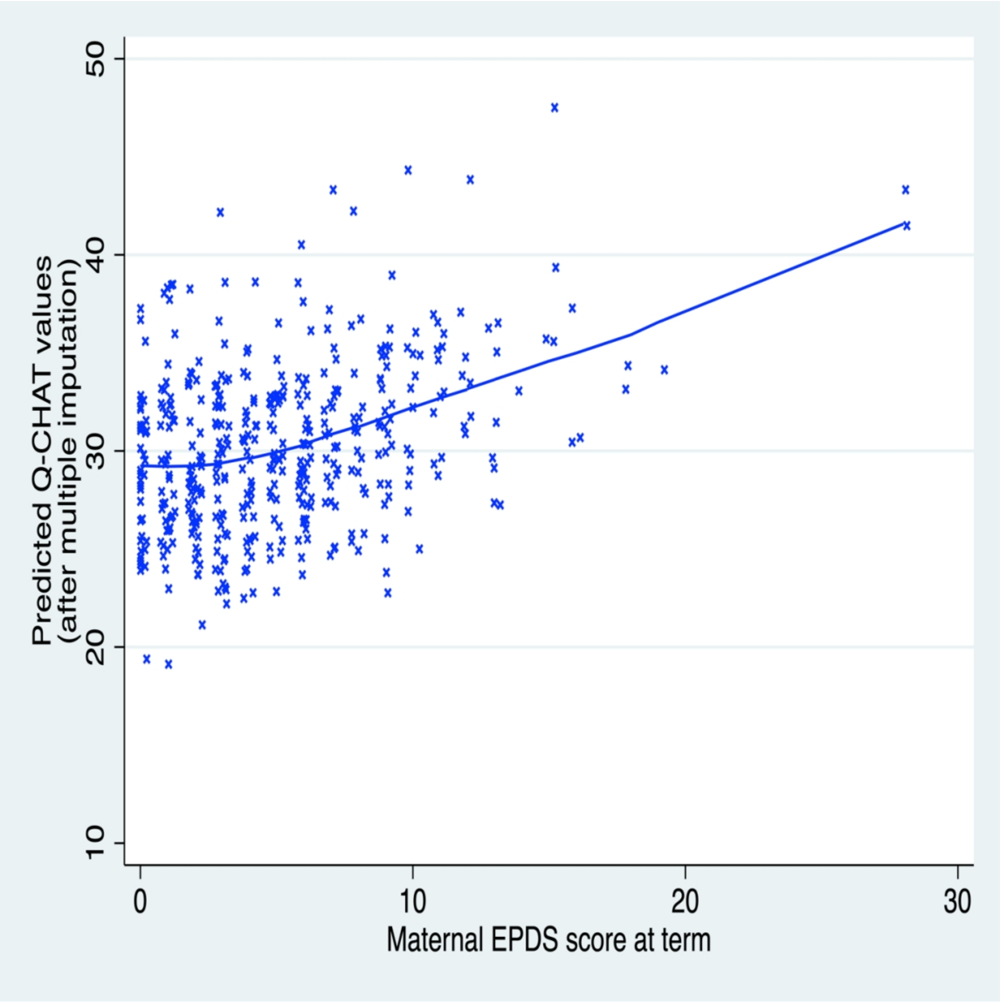

Fig.2 Children's predicted Q-CHAT scores at 18 months are positively correlated to the maternal EPDS score at term-equivalent age.

158x158mm (220 x 220 DPI)

Supplemental material

Supplementary Table 1: CBCL internalising symptom model predictors using multiple imputation.

Supplementary Table 2: CBCL externalising symptom model predictors using multiple imputation.

Supplementary Table 3: CBCL and Q-CHAT model predictors using complete case analysis without interaction.

Supplementary Table 4: EPDS score predictors.

Supplementary Table 5: EPDS score predictors including interaction ‘term x time-lag’.

Supplementary Figure 1: Histogram showing the distribution of maternal EPDS scores at term-equivalent age.

For peer review only

Supplementary Table 1: CBCL internalising symptom model predictors using multiple imputation**.**

	**B [95%CI]**	**p**	**f^2**
**Maternal EPDS**	0.22 [0.08, 0.36]	.003 **	0.03
**Maternal BMI**	-0.04 [-0.13, 0.06]	.436	-
**Multiple pregnancy**	0.58 [-1.10, 2.27]	.497	-
**Parity**			
**1**	-0.37 [-1.41, 0.67]	.487	-
**2**	-1.33 [-3.09, 0.42]	.136	-
**3+**	0.64 [-1.34, 2.62]	.524	-
**IMD rank**	-0.41 [-0.91, 0.10]	.115	-
**Gestational age at birth (weeks)**	0.22 [-0.00, 0.44]	.053	-
**Birthweight (kg)**	-0.97 [-1.90, -0.05]	.038 *	0.005
**Sex: female**	-0.51 [-1.35, 0.33]	.232	-
**Corrected age at assessment (months)**	0.02 [-0.41, 0.45]	.923	-
**Cognition**	-0.05 [-0.10, -0.01]	.016 *	0.01

p<0.05 *; p<0.01 **; p<0.001 ***
Adjusted R^2 = 0.0566.
B = unstandardised coefficient.
Outcome variable = Child Behaviour Checklist internalising sub-score at 18 months. Maternal EPDS = maternal Edinburgh Postnatal Depression Scale score at term-equivalent age. Multiple pregnancy = dummy variable of twin/triplet pregnancy. Parity = dummy variable, one/two/three+ previous child(ren). Corrected age at assessment (months) = age at behavioural assessment, corrected for gestational age. Cognition = infant Bayley III score at 18 months.
Effect size (Cohen's f^2, calculated from squared part correlations for predictors significant to 0.05): 0.02 = small, 0.15 = medium and 0.35 = large.[1]
- indicates data not given, as predictor not significant to 0.05.

For peer review only

Supplementary Table 2: CBCL externalising symptom model predictors using multiple imputation.

	B [95%CI]	p	f^2
**Maternal EPDS**	0.40 [0.20, 0.61]	<.001 ***	0.05
**Maternal BMI**	0.01 [-0.17, 0.15]	.933	-
**Multiple pregnancy**	2.51 [-0.29, 5.31]	.079	-
**Parity**			
**1**	-1.06 [-2.53, 0.42]	.160	-
**2**	-0.61 [-3.55, 2.33]	.682	-
**3+**	-0.96 [-4.47, 2.56]	.593	-
**IMD rank**	-0.24 [-1.11, 0.63]	.585	-
**Gestational age at birth (weeks)**	-0.07 [-0.40, 0.27]	.701	-
**Birthweight (kg)**	1.03 [-0.38, 2.44]	.153	-
**Sex: female**	-1.80 [-3.07, -0.53]	.006 **	0.06
**Corrected age at assessment (months)**	-0.40 [-0.95, 0.16]	.161	-
**Cognition**	0.03 [-0.03, 0.10]	.322	-

p<0.05 *; p<0.01 **; p<0.001 ***
Adjusted R^2 = 0.0612.
B = unstandardised coefficient.
Outcome variable = Child Behaviour Checklist externalising sub-score at 18 months. Maternal EPDS = maternal Edinburgh Postnatal Depression Scale score at term-equivalent age. Multiple pregnancy = dummy variable of twin/triplet pregnancy. Parity = dummy variable, one/two/three+ previous child(ren). Corrected age at assessment (months) = age at behavioural assessment, corrected for gestational age. Cognition = infant Bayley III score at 18 months.
Effect size (Cohen's f^2, calculated from squared part correlations for predictors significant to 0.05): 0.02 = small, 0.15 = medium and 0.35 = large.[1]
- indicates data not given, as predictor not significant to 0.05.

For peer review only

Supplementary Table 3: CBCL and Q-CHAT model predictors using complete case analysis without interaction.

	CBCL		Q-CHAT	
	**B [95%CI]**	**p**	**B [95%CI]**	**p**
**Maternal EPDS**	0.88 [0.35, 1.41]	.001 **	0.21 [-0.02, 0.44]	.069
**Maternal BMI**	-0.01 [-0.38, 0.37]	.963	0.01 [-0.16, 0.17]	.930
**Multiple pregnancy**	1.50 [-6.87, 9.87]	.724	0.43 [-2.50, 3.37]	.772
**Parity**				
**1**	-2.83 [-6.60, 0.94]	.141	-1.54 [-3.41, 0.34]	.108
**2**	-3.49 [-10.3, 3.35]	.316	0.13 [-2.85, 3.10]	.933
**3+**	-1.38 [-10.0, 7.30]	.755	-1.43 [-4.50, 1.64]	.360
**IMD rank**	-1.44 [-3.56, 0.67]	.181	-1.75 [-2.70, -0.79]	<.001 ***
**Gestational age at birth (weeks)**	0.01 [-0.90, 0.91]	.987	0.10 [-0.33, 0.54]	.639
**Birthweight (kg)**	-0.65 [-4.38, 3.08]	.733	-1.81 [-3.63, 0.00]	.050
**Sex: female**	-4.57 [-7.82, -1.31]	.006 **	-2.12 [-3.60, -0.64]	.005 **
**Corrected age at assessment (months)**	-0.84 [-2.26, 0.59]	.247	-0.41 [-1.18, 0.35]	.290
**Cognition**	-0.03 [-0.20, 0.13]	.689	-0.23 [-0.30, -0.15]	<.001 ***

p<0.05 *; p<0.01 **; p<0.001 ***
CBCL adjusted $R^2 = 0.0862$. Q-CHAT adjusted $R^2 = 0.2103$.
B = unstandardised coefficient.
CBCL = Child Behaviour Checklist externalising sub-score at 18 months. Q-CHAT = Quantitative Checklist for Autism in Toddlers score at 18 months. Maternal EPDS = maternal Edinburgh Postnatal Depression Scale score at term-equivalent age. Multiple pregnancy = dummy variable of twin/triplet pregnancy. Parity = dummy variable, one/two/three+ previous child(ren). Gestation (weeks) = dummy variable: 34-36+6 weeks and ≥37 weeks gestation at birth. Corrected age at assessment (months) = age at behavioural assessment, corrected for gestational age. Cognition = infant Bayley III score at 18 months.

For peer review only

Supplementary Table 4: EPDS score predictors.

	IRR [95%CI]	p
**Time-lag (weeks)**	1.01 [0.97, 1.05]	.647
**Gestation:term**	0.91 [0.64, 1.31]	.627
**IMD rank**	1.00 [1.00, 1.00]	.103
**Multiple pregnancy**	0.66 [0.46, 0.96]	.031 *
**Parity**		
**1**	0.79 [0.66, 0.95]	.011 *
**2**	0.87 [0.60, 1.28]	.491
**3+**	0.84 [0.54, 1.31]	.445
**Birthweight (kg)**	0.98 [0.83, 1.17]	.847
**Sex:female**	1.13 [0.98, 1.31]	.098

$p<0.05$ *; $p<0.01$ **; $p<0.001$ ***

Pseudo $R^2 = 0.0228$

IRR = incidence rate ratio

Outcome variable = maternal Edinburgh Postnatal Depression Scale (EPDS) score at term-equivalent age. Time-lag (weeks) = time in weeks between birth and EPDS assessment. Gestation:term = dummy variable, term (≥37 weeks) vs preterm (<37 weeks) gestation at birth. Multiple pregnancy = dummy variable of twin/triplet pregnancy. Parity = dummy variable, one/two/three+ previous child(ren).

For peer review only

Supplementary Table 5: EPDS score predictors including interaction 'term x time-lag'.

	IRR [95%CI]	p
**Time-lag (weeks)**	1.00 [0.97, 1.04]	.823
**Gestation: term**	0.88 [0.58, 1.34]	.553
**IMD rank**	1.00 [1.00, 1.00]	.104
**Multiple pregnancy**	0.66 [0.46, 0.96]	.029 *
**Parity**		
**1**	0.79 [0.66, 0.95]	.010 *
**2**	0.87 [0.60, 1.28]	.480
**3+**	0.85 [0.55, 1.31]	.458
**Birthweight (kg)**	0.97 [0.83, 1.15]	.756
**Sex:female**	1.13 [0.98, 1.30]	.100
**Term x time-lag (weeks)**	1.01 [0.94, 1.10]	.735

$p<0.05$ *; $p<0.01$ **; $p<0.001$ ***

Pseudo $R^2 = 0.0230$

IRR = incidence rate ratio

Outcome variable = maternal Edinburgh Postnatal Depression Scale (EPDS) score at term-equivalent age. Time-lag (weeks) = time in weeks between birth and EPDS assessment. Gestation: term = dummy variable, term (≥37 weeks) vs preterm (<37 weeks) gestation at birth. Multiple pregnancy = dummy variable of twin/triplet pregnancy. Parity = dummy variable, one/two/three+ previous child(ren). Term x time-lag (weeks): interaction term between term gestation at birth and time-lag between birth and maternal EPDS assessment.

For peer review only

Supplementary Figure 1: Histogram showing the distribution of maternal EPDS scores at term-equivalent age.

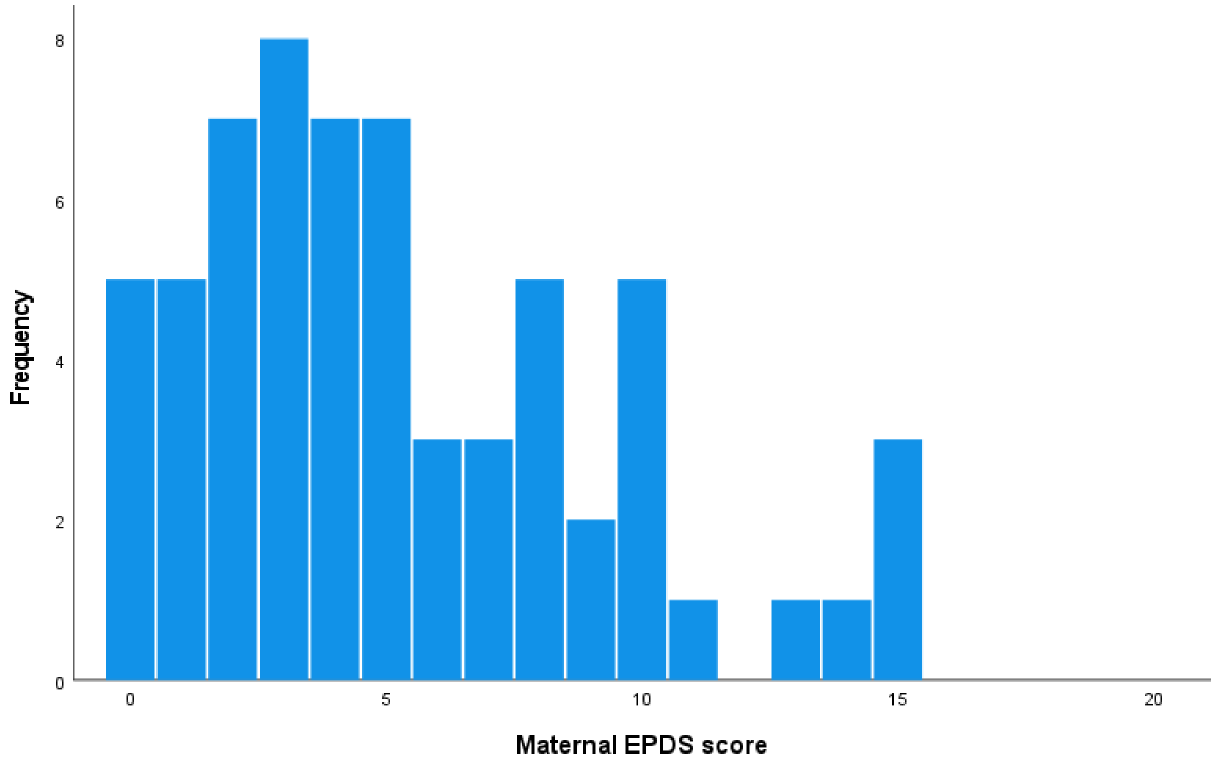

Supplemental reference list

1. Cohen J. *Statistical Power Analysis for the Behavioral Sciences*. 2nd ed. L. Erlbaum Associates; 1988.

For peer review only

STROBE Statement—Checklist of items that should be included in reports of ***cohort studies***

	Item No	Recommendation	Page No
**Title and abstract**	1	(*a*) Indicate the study's design with a commonly used term in the title or the abstract<br>(*b*) Provide in the abstract an informative and balanced summary of what was done and what was found	1, 2
**Introduction**			
Background/rationale	2	Explain the scientific background and rationale for the investigation being reported	5, 6
Objectives	3	State specific objectives, including any prespecified hypotheses	6
**Methods**			
Study design	4	Present key elements of study design early in the paper	6, 7
Setting	5	Describe the setting, locations, and relevant dates, including periods of recruitment, exposure, follow-up, and data collection	7
Participants	6	(*a*) Give the eligibility criteria, and the sources and methods of selection of participants. Describe methods of follow-up<br>(*b*) For matched studies, give matching criteria and number of exposed and unexposed	7
Variables	7	Clearly define all outcomes, exposures, predictors, potential confounders, and effect modifiers. Give diagnostic criteria, if applicable	7, 8
Data sources/ measurement	8*	For each variable of interest, give sources of data and details of methods of assessment (measurement). Describe comparability of assessment methods if there is more than one group	7, 8
Bias	9	Describe any efforts to address potential sources of bias	9
Study size	10	Explain how the study size was arrived at	7
Quantitative variables	11	Explain how quantitative variables were handled in the analyses. If applicable, describe which groupings were chosen and why	9, 10
Statistical methods	12	(*a*) Describe all statistical methods, including those used to control for confounding<br>(*b*) Describe any methods used to examine subgroups and interactions<br>(*c*) Explain how missing data were addressed<br>(*d*) If applicable, explain how loss to follow-up was addressed<br>(*e*) Describe any sensitivity analyses	9, 10
**Results**			
Participants	13*	(a) Report numbers of individuals at each stage of study—eg numbers potentially eligible, examined for eligibility, confirmed eligible, included in the study, completing follow-up, and analysed<br>(b) Give reasons for non-participation at each stage<br>(c) Consider use of a flow diagram	11
Descriptive data	14*	(a) Give characteristics of study participants (eg demographic, clinical, social) and information on exposures and potential confounders<br>(b) Indicate number of participants with missing data for each variable of interest<br>(c) Summarise follow-up time (eg, average and total amount)	11
Outcome data	15*	Report numbers of outcome events or summary measures over time	11, 12

For peer review only - http://bmjopen.bmj.com/site/about/guidelines.xhtml

Main results	16	(*a*) Give unadjusted estimates and, if applicable, confounder-adjusted estimates and their precision (eg, 95% confidence interval). Make clear which confounders were adjusted for and why they were included<br>(*b*) Report category boundaries when continuous variables were categorized<br>(*c*) If relevant, consider translating estimates of relative risk into absolute risk for a meaningful time period	11, 12, 13
Other analyses	17	Report other analyses done—eg analyses of subgroups and interactions, and sensitivity analyses	11, 12, 13
**Discussion**			
Key results	18	Summarise key results with reference to study objectives	13
Limitations	19	Discuss limitations of the study, taking into account sources of potential bias or imprecision. Discuss both direction and magnitude of any potential bias	15-17
Interpretation	20	Give a cautious overall interpretation of results considering objectives, limitations, multiplicity of analyses, results from similar studies, and other relevant evidence	17
Generalisability	21	Discuss the generalisability (external validity) of the study results	15-17
**Other information**			
Funding	22	Give the source of funding and the role of the funders for the present study and, if applicable, for the original study on which the present article is based	3

*Give information separately for exposed and unexposed groups.

Note: An Explanation and Elaboration article discusses each checklist item and gives methodological background and published examples of transparent reporting. The STROBE checklist is best used in conjunction with this article (freely available on the Web sites of PLoS Medicine at http://www.plosmedicine.org/, Annals of Internal Medicine at http://www.annals.org/, and Epidemiology at http://www.epidem.com/). Information on the STROBE Initiative is available at http://www.strobe-statement.org.

For peer review only

For peer review only - http://bmjopen.bmj.com/site/about/guidelines.xhtml

BMJ Open

Postnatal maternal depressive symptoms and behavioural outcomes in term- and preterm-born toddlers: a longitudinal UK community cohort study.

Journal:	*BMJ Open*
Manuscript ID	bmjopen-2021-058540.R2
Article Type:	Original research
Date Submitted by the Author:	26-May-2022
Complete List of Authors:	Kleine, Ira; King's College London, Centre for the Developing Brain, School of Bioengineering and Imaging Sciences, Faculty of Life Sciences & Medicine<br>Vamvakas, George; King's College London, Department of Biostatistics and Health Informatics, Institute of Psychiatry, Psychology and Neuroscience<br>Lautarescu, Alexandra; King's College London, Centre for the Developing Brain, School of Bioengineering and Imaging Sciences, Faculty of Life Sciences & Medicine; King's College London, Forensic and Neurodevelopmental Sciences, Institute of Psychiatry, Psychology and Neuroscience<br>Falconer, Shona; King's College London, Centre for the Developing Brain, School of Bioengineering and Imaging Sciences, Faculty of Life Sciences & Medicine<br>Chew, Andrew; King's College London, Centre for the Developing Brain, School of Bioengineering and Imaging Sciences, Faculty of Life Sciences & Medicine<br>Counsell, Serena ; King's College London, Centre for the Developing Brain, School of Bioengineering and Imaging Sciences, Faculty of Life Sciences & Medicine<br>Pickles, Andrew; King's College London, Department of Biostatistics and Health Informatics, Institute of Psychiatry, Psychology and Neuroscience<br>Edwards, David; King's College London, Centre for the Developing Brain, School of Bioengineering and Imaging Sciences, Faculty of Life Sciences & Medicine<br>Nosarti, Chiara; King's College London, Centre for the Developing Brain, School of Bioengineering and Imaging Sciences, Faculty of Life Sciences & Medicine; King's College London, Department of Child and Adolescent Psychiatry, Institute of Psychiatry, Psychology and Neuroscience
**Primary Subject Heading**:	Mental health
Secondary Subject Heading:	Paediatrics
Keywords:	MENTAL HEALTH, Child & adolescent psychiatry < PSYCHIATRY, Developmental neurology & neurodisability < PAEDIATRICS, Depression & mood disorders < PSYCHIATRY, NEONATOLOGY

SCHOLARONE™
Manuscripts

I, the Submitting Author has the right to grant and does grant on behalf of all authors of the Work (as defined in the below author licence), an exclusive licence and/or a non-exclusive licence for contributions from authors who are: i) UK Crown employees; ii) where BMJ has agreed a CC-BY licence shall apply, and/or iii) in accordance with the terms applicable for US Federal Government officers or employees acting as part of their official duties; on a worldwide, perpetual, irrevocable, royalty-free basis to BMJ Publishing Group Ltd ("BMJ") its licensees and where the relevant Journal is co-owned by BMJ to the co-owners of the Journal, to publish the Work in this journal and any other BMJ products and to exploit all rights, as set out in our licence.

The Submitting Author accepts and understands that any supply made under these terms is made by BMJ to the Submitting Author unless you are acting as an employee on behalf of your employer or a postgraduate student of an affiliated institution which is paying any applicable article publishing charge ("APC") for Open Access articles. Where the Submitting Author wishes to make the Work available on an Open Access basis (and intends to pay the relevant APC), the terms of reuse of such Open Access shall be governed by a Creative Commons licence – details of these licences and which Creative Commons licence will apply to this Work are set out in our licence referred to above.

Other than as permitted in any relevant BMJ Author's Self Archiving Policies, I confirm this Work has not been accepted for publication elsewhere, is not being considered for publication elsewhere and does not duplicate material already published. I confirm all authors consent to publication of this Work and authorise the granting of this licence.

For peer review only

Postnatal maternal depressive symptoms and behavioural outcomes in term- and preterm-born toddlers: a longitudinal UK community cohort study.

Kleine, I[a] (MBBS); Vamvakas, G[b] (MSc); Lautarescu A[a,c] (MPhil); Falconer, S[a] (PhD); Chew A[a] (MD); Counsell SJ[a] (PhD); Pickles, A[b] (FMedSci); Edwards, AD[a] (FMedSci); Nosarti, C[a,d] (PhD).

[a] Centre for the Developing Brain, School of Bioengineering and Imaging Sciences, Faculty of Life Sciences & Medicine, King's College London, St Thomas' Hospital, London SE1 7EH, UK.

[b] Department of Biostatistics and Health Informatics, Institute of Psychiatry, Psychology and Neuroscience, King's College London, London SE5 8AF, UK.

[c] Forensic and Neurodevelopmental Sciences, Institute of Psychiatry, Psychology and Neuroscience, King's College London, London SE5 8AF, UK.

[d] Department of Child and Adolescent Psychiatry, Institute of Psychiatry, Psychology and Neuroscience, King's College London, London SE5 8AF, UK.

Post-publication corresponding author's contact information

Prof Chiara Nosarti

Centre for the Developing Brain, School of Bioengineering and Imaging Sciences

King's College London

St Thomas' Hospital

London

SE1 7EH

UK

chiara.nosarti@kcl.ac.uk

+44 (0)207 848 0133

For peer review only - http://bmjopen.bmj.com/site/about/guidelines.xhtml

Abstract

Objectives: To examine the association between maternal depressive symptoms in the immediate postnatal period and offspring's behavioural outcomes in a large cohort of term- and preterm-born toddlers.

Design and Participants: Data were drawn from the Developing Human Connectome Project. Maternal postnatal depressive symptoms were assessed at term-equivalent age, and children's outcomes were evaluated at a median corrected age of 18.4 months (range 17.3 – 24.3).

Exposure and outcomes: Preterm birth was defined as <37 weeks completed gestation. Maternal depressive symptoms were assessed with the Edinburgh Postnatal Depression Scale (EPDS). Toddlers' outcome measures were parent-rated Child Behaviour Checklist $1^{1/2}$-5 Total (CBCL) and Quantitative Checklist for Autism in Toddlers (Q-CHAT) scores. Toddlers' cognition was assessed with the Bayley Scales of Infant and Toddler Development – Third Edition (Bayley-III).

Results: Higher maternal EPDS scores were associated with toddlers' higher CBCL (B=0.93, 95% CI 0.43-1.44, $p<0.001$, $f^2=0.05$) and Q-CHAT scores (B=0.27, 95% CI 0.03-0.52, p=.031, $f^2=0.01$). Maternal EPDS, toddlers' CBCL and Q-CHAT scores did not differ between preterm (n=97; 19.1% of the total sample) and term participants. Maternal EPDS score did not disproportionately affect preterm children with respect to CBCL or Q-CHAT scores.

Conclusions: Our findings indicate that children whose mothers reported increased depressive symptoms in the early postnatal period, including subclinical symptoms, exhibit more parent-reported behavioural problems in toddlerhood. These associations were independent of gestational age. Further research is needed to confirm the clinical significance of these findings.

For peer review only - http://bmjopen.bmj.com/site/about/guidelines.xhtml

Strengths and limitations of this study

- Prospective study with a large sample, using multiple imputation to reduce non-response bias.
- Maternal depressive symptoms assessed as a continuous variable, providing more nuanced information about the significance of subclinical symptoms.
- Maternal depressive symptoms assessed earlier than in previous studies, enabling recognition of early screening opportunities for families.
- Potential common method variance bias through parent-completed child behavioural assessments.
- Unknown paternal and parental factors, such as comorbid psychiatric conditions, that may confound our findings.

Keywords

Postpartum Depression; Child Development; Autism; Mental Health; Child Behavior; Mood Disorder; Preterm Birth.

Declarations

Funding

The DHCP project was funded by the European Research Council under the European Union Seventh Framework Programme (FR/2007-2013)/ERC Grant Agreement no. 319456. The authors acknowledge infrastructure support from the National Institute for Health Research Mental Health Biomedical Research Centre at South London, Maudsley NHS Foundation Trust, King's College London, the National Institute for Health Research Mental Health Biomedical Research Centre at Guys, and St Thomas' Hospitals NHS Foundation Trust. The study was also supported in part by the Engineering and Physical Sciences Research Council / Wellcome Trust Centre for Medical Engineering at King's College London (grant WT 203148/Z/16/Z) and the Medical Research Council (UK) (grants MR/K006355/1 and MR/L011530/1) and the MRC Centre for Neurodevelopmental Disorders at King's College London. AP receives a NIHR SI award (NF-SI-0617-10120). AL is supported by the UK Medical Research Council (MR/N013700) and King's College London member of the MRC Doctoral Training Partnership in Biomedical Sciences. The views expressed are those of the authors and not necessarily those of the NHS, the NIHR, or the Department of Health and Social Care.

For peer review only - http://bmjopen.bmj.com/site/about/guidelines.xhtml

Conflict of interest / Competing interests

ADE received financial support from the EU-AIMS-Trials (European Research Council under the European Union Seventh Framework Programme) as co-Principal Investigator. ADE received consulting fees from Chiesi Farmaceutici (advice on neuroprotection in newborn infants) and Medtronix (unpaid participation in scientific advice committee). ADE has a patent on Xenon as an organ protectant (No P023708WO). ADE was Chair of the Data Monitoring and Ethics Committee for the Baby-Oscar Trial, and served on the Data Monitoring and Ethics Committee for the PAEN Trial.

There are no other relationships or activities that could appear to have influenced the submitted work.

Availability of data and material

Research data are available upon reasonable request.

Code availability

Not applicable.

Ethics approval

This study was approved by the UK National Research Ethics Authority (14/LO/1169) and conducted in accordance with the World Medical Association's Code of Ethics (Declaration of Helsinki).

Consent to participate

Written informed consent was given by children's carer(s) at recruitment into the study.

Consent for publication

Not applicable.

For peer review only - http://bmjopen.bmj.com/site/about/guidelines.xhtml

Introduction

Postnatal depression affects approximately 12% of mothers worldwide.[1] In contrast to 'baby blues', which is a state of emotional lability that affects between 13.7%-76.0% of women in the first few days after birth and typically resolves spontaneously within two weeks,[2] postnatal depression is more severe and starts in the first few months post-partum[1]. Stressful life events have been linked to a heightened risk of developing postnatal depression;[3] for example, mothers of preterm infants have a significantly higher risk of postpartum depression compared to mothers of term infants,[4] likely due to heightened stress associated with perinatal complications.[5]

Women with postnatal depression tend to be less responsive to their baby's needs and to display less affection.[6] Therefore, in the short-term postpartum depression may affect mother-infant interactions[7] and in the long-term it may lead to alterations in brain development,[8] emotional difficulties,[9] less secure attachment, cognitive and behavioural problems in childhood, and a possible increased risk of autism spectrum disorder (ASD).[10,11] Large cohort studies, such as the Avon Longitudinal Study of Parents and Children (ALSPAC), have shown that these associations are even evident when maternal depression is measured on a continuum of symptoms rather than a dichotomous diagnosis,[12–14] supporting the notion that elevated sub-diagnostic psychiatric symptoms can also negatively impact on children's development.[15]

Studies investigating the underlying causes that may link maternal postnatal depression to child outcomes have implicated several biological and environmental variables. For instance, genetic and epigenetic factors have been shown to both mediate and mitigate the intergenerational transmission of psychiatric disorders,[16] while lower quality parenting, interparental conflict, and socioeconomic deprivation have been shown to exacerbate children's developmental risk of emotional and behavioural problems.[11] In addition, being born preterm (i.e. <37 weeks' gestation, as per the World Health Organization definition [17]) has been associated with alterations in early brain development,[18] as well as neurological, behavioural and cognitive problems in childhood and beyond.[19,20] Therefore, it is complex to disentangle the possible effects of postnatal maternal mental health and those of perinatal clinical factors on specific outcomes in preterm children, as these may involve both maternal psychosocial and biological variables, as well as child preterm-related neurodevelopmental morbidity.

For peer review only - http://bmjopen.bmj.com/site/about/guidelines.xhtml

Furthermore, a question that remains unanswered is whether preterm birth accentuates the association between maternal postnatal depression and child outcome. Two theoretical frameworks exist that hypothesise certain infants may be influenced differently by external stimuli: the diathesis stress model proposes that certain vulnerability factors make affected infants more prone to suboptimal environmental influences with subsequent poorer outcomes,[21,22] whereas the differential susceptibility model frames such factors as plasticity-mediating, thus leading to poorer outcomes in negative environments, as well as better outcomes in supportive environments.[22,23] Previous studies investigating differential susceptibility have shown mixed findings studying a range of environmental and clinical exposures,[24,25] with child outcomes including attachment, internalising and externalising behaviour, and academic competence.[25] Both low birthweight in term infants (small for gestational age, SGA) [26] and preterm birth (PTB) [23,24,27] have been explored as distinct potential susceptibility factors. This distinction is based on the different pathophysiological processes underlying the respective conditions of SGA and PTB, both, or a combination, of which can cause low birthweight.[28] For example, SGA is a marker of intra-uterine growth restriction related to placental dysfunction,[29] whereas PTB can be caused by a multitude of factors, including infection and inflammation.[30]

Given that mothers of preterm children experience elevated levels of distress,[31] are at high risk of developing postnatal depression,[32] and that preterm children themselves are vulnerable to psychiatric sequelae,[33] in addition to investigating the association between very early maternal postnatal depressive symptoms and child behavioural and emotional outcomes, we further aimed to investigate the interaction between preterm birth and maternal depressive symptoms on child outcomes. Previous work focusing on the differential susceptibility of preterm born children to various environmental stimuli, as described above, had not yet studied maternal depressive symptoms as a proposed exposure. We specifically aimed to investigate the continuum of maternal depressive symptoms rather than solely focussing on clinically significant maternal depression, so as to provide more nuanced information about the importance of subclinical depressive symptoms on child outcomes. We hypothesised that early postnatal maternal depressive symptoms would be more elevated in mothers of preterm compared to term infants and that these would impact preterm children's behavioural and emotional outcomes to a greater degree than their term counterparts.

For peer review only - http://bmjopen.bmj.com/site/about/guidelines.xhtml

Methods

Sample

Participants were enrolled in the Developing Human Connectome Project (DHCP, http://www.developingconnectome.org/), a neuroimaging-focused project, with eligibility criteria including pregnant women (aged ≥16 years) with a gestational age of 20–42 weeks, and newborn infants aged 24–44 weeks; infants enrolled in the DHCP had magnetic resonance imaging (MRI) at term-equivalent age. Exclusion criteria for the DHCP included: contraindications to MRI, babies being too unwell to tolerate a scan, and language difficulties preventing informed consent.[34] Toddlers were invited to the Centre for the Developing Brain, St Thomas' Hospital, London, for neurodevelopmental assessment at 18 months post-expected delivery date; appointments were made according to family availability as close as possible to this time-point. Inclusion criteria for our follow-up study were: mother and baby attendance for MRI at term-equivalent age; completed toddler neurodevelopmental assessment. These inclusion criteria were met by 509 toddlers by the date of closure for this analysis (26/02/2020). Of the 509 toddlers, 51 were one of a twin pregnancy, and three were one of a triplet pregnancy; the sample contained 22 sibling pairs and one set of triplets. This study was approved by the UK National Research Ethics Authority (14/LO/1169) and conducted in accordance with the World Medical Association's Code of Ethics (Declaration of Helsinki). Written informed consent was given by children's carer(s) at recruitment into the study.

Maternal variables

Maternal age, parity, Body Mass Index (BMI), ethnicity and postcode were collected at enrolment into the DHCP study. Our sample was ethnically representative of the surrounding geographical area. Parity was coded as 0, 1, 2, or ≥3 previous children. Index of Multiple Deprivation (IMD) rank was computed from the current maternal postcode using the 2019 IMD classification; it combines locality-specific information about income, employment, education, health, crime, housing and living environment, thus providing a proxy for family socioeconomic status.[35] Lower IMD rank corresponds to greater social deprivation. Our sample was generally less deprived than the surrounding geographical areas, as well as the UK as a whole, reflecting trends observed in other UK longitudinal studies.[36]

Maternal depressive symptoms were measured using the Edinburgh Postnatal Depression Scale (EPDS)[37] on the day of infant's MRI at term-equivalent age. Mothers of infants born at

For peer review only - http://bmjopen.bmj.com/site/about/guidelines.xhtml

term were tested in the first few weeks postnatally, whereas mothers of preterm-born infants were tested once they reached term-corrected age. The EPDS is a 10-item screening questionnaire completed by mothers, with higher scores reflecting a higher likelihood of depressive disorders. A score of 13 can be used as a cut-off indicating high-level symptoms, although a cut-off of 11 maximises the sensitivity and specificity of the screening tool for depression.[38] Mothers completed the EPDS independently in a private room in our Centre, with no interaction with the researcher. Participants were informed that the results would be discussed with them, and consented to information being shared with their General Practitioner in the case of high scores. The EPDS questionnaire was scored by a member of the DHCP team.[34]

Child variables

Infant *clinical characteristics* were gathered from clinical notes where available, or from maternal report, and included: sex, gestational age at birth, birth weight, and pregnancy size (singleton/twin/triplet).

Behavioural outcomes were assessed using the Child Behaviour Checklist/$1^{1/2}$-5 (CBCL), a parent-completed 100-item questionnaire, in which the parent rates the child's behaviour over the preceding two months using a 3-point Likert scale ("not true", "somewhat or sometimes true", and "very true or often true"). Responses are categorised into syndrome profiles, and these are subsequently grouped into internalising (emotional reactivity, anxiety/depression, somatic complaints, and withdrawal), externalising (attention problems, aggressive behaviour) and total (internalising, externalising, sleep and other) problem scales. Higher scores indicate increased emotional and behavioural problems. Total scores are classified into a normal range (<83rd centile, T <60), borderline range (83rd-90th centile, T 60-63), and clinical range (>90th centile, T ≥64).[39] The CBCL is known to have high reliability, validity and cross-informant agreement for measuring children's emotional and behavioural problems.[39]

We used the Quantitative Checklist for Autism in Toddlers (Q-CHAT) as an additional behavioural screening tool to broaden the exploration of mental health outcomes in toddlers. The Q-CHAT is a parent-completed 25-item questionnaire, in which the child's behaviour is scored on a 5-point (0-4) frequency scale. Higher total scores correspond to a higher frequency of behaviours also observed in autism spectrum conditions. The Q-CHAT shows

For peer review only - http://bmjopen.bmj.com/site/about/guidelines.xhtml

good test-retest reliability, face validity and specificity, yet poor positive predictive value for autism,[40,41] highlighting that higher Q-CHAT scores may reflect developmental immaturity rather than autism.[41]

Cognitive assessment was performed using the Bayley Scales of Infant and Toddler Development – Third Edition (Bayley-III). The Bayley-III provides scores for a child's overall cognitive, language and motor development. The cognitive standardised composite score was used in this study; scores between 70-84 indicate mild cognitive impairment, scores between 55-69 indicate moderate impairment, and scores lower than 55 indicate severe impairment[42]. Reliability and validity of the Bayley-III have been shown to be robust,[43] although some studies report its underestimation of developmental problems.[44]

Assessments were carried out by staff experienced in the neurocognitive assessments of toddlers.

Analysis

Descriptive statistics and one-way ANOVA tests were performed in IBM SPSS Statistics for Windows v.25. All other analyses were carried out in Stata v.16.

Multiple imputation (MI) was carried out to account for missing data in CBCL (11/509, 2.24%), Q-CHAT (9/509, 1.8%), maternal EPDS (73/509, 14.3%), maternal BMI (27/509, 5.3%) and IMD rank (3/509, 0.6%). Variables were imputed simultaneously using the 'mi impute chained' procedure that performs imputation by chained equations. The imputation models had the same structural form as the analysis models, and included all variables that appear in the corresponding analysis models (maternal EPDS, maternal BMI, multiple pregnancy, parity, IMD rank, gestational age at birth, birthweight, sex, corrected age at assessment, and Bayley III Cognitive Composite score); in addition, maternal age was also included in the imputation model because it was found to be a significant predictor of both the total CBCL raw score and the Q-CHAT score when it was included as the sole independent variable in linear regression models.

Maternal EPDS and CBCL were imputed using Poisson regression; Q-CHAT, maternal BMI, and the IMD rank were imputed using linear regression. 40 MI datasets were created. To assess the stability of our MI parameters, we extracted the Monte Carlo error of each

For peer review only - http://bmjopen.bmj.com/site/about/guidelines.xhtml

parameter estimate and examined whether the error for the coefficient was less than 10% of the parameter's standard error estimate. MI estimates were used for the primary analyses and compared to the estimates from complete-case (CC, individuals who had no missing data pre-imputation) analyses. Normal probability plots of residuals from the CC analyses were examined.

The analysis models were multiple linear regressions fitted using the 'mi estimate' procedure, which estimates effects after application of Rubin's rules.[45] To account for the small amount of clustering in our data (twin/triplet siblings), the models' standard errors were obtained using Stata's robust cluster estimator 'vce(cluster *idvar*)'. For continuous variables, Cohen's f-squared effect sizes were calculated using $f^2 = (R^2_{AB} - R^2_A)/(1 - R^2_{AB})$, where R^2_{AB} is the R-squared value from a regression model that includes the variable of interest as well as all the covariates used in the model, and R^2_A is the R-squared value from the regression model that includes only the covariates.[46,47] For binary variables, Cohen's f-squared effect sizes were produced after estimating first the Cohen's d using the formula: $f^2 = \frac{d}{2k}$, where k is the number of groups. As a measure of dispersion, Cohen's d used the average root mean-square error over the MI datasets. Adjusted R-squared values after MI were extracted after estimating the model with the user-written 'mibeta' command with the 'fisherz' option,[48] which calculates R-squared measures for linear regression with MI data. The significance of the joint effect of the categorical variable parity was assessed using `mi test' which performs Wald tests of composite linear hypotheses.

Primary outcome measures were children's total CBCL raw score and Q-CHAT score. *Secondary outcome measures* were CBCL internalising and externalising scores. The effect of maternal EPDS score was adjusted for IMD rank, maternal age, maternal BMI, maternal parity, pregnancy size, and the following child's variables: continuous gestational age, birth weight, Bayley-III cognitive composite score, and corrected age at assessment. The interaction between preterm birth and maternal depressive symptoms was explored using a complete case analysis in both CBCL and Q-CHAT models, using a dichotomised measure of gestational age. EPDS, CBCL and Q-CHAT scores were compared between term (≥37 weeks gestation) and preterm infants (<37 weeks gestation) using the complete case dataset. Our regressions were thus run twice: with and without the interaction term.

For peer review only - http://bmjopen.bmj.com/site/about/guidelines.xhtml

As all mothers had their EPDS score measured near term (or term-corrected in the case of mothers of preterm infants), we further investigated the association between time elapsing between baby's birth and mother's EPDS assessment and EPDS score, in order to avoid erroneously identifying 'baby blues' in mothers of term-born infants versus postnatal depression in mothers of preterm infants. This post-hoc analysis was performed using Poisson regression.

Patient and Public Involvement

The current study was developed in consultation with the Weston Programme for Family Centered Research, which involves parents to define what research is valuable to them, and to allow them to lead it with support from the scientists in the Centre for the Developing Brain.

Results

Descriptive statistics

Our sample of 509 toddlers were followed up at a median corrected age of 18.4 months (range 17.3 – 24.3 months). 51 (10.0%) of these were twins, and 3 (0.59%) were triplets. Of the 509, 21 (4.13%) mothers scored above a clinical cut-off (≥13) on the EPDS;(26) the distribution of maternal EPDS scores is shown graphically in Supplementary Figure 1. Demographic data are shown in Table 1. Complete data were available for 400 (78.6%) participants. Missing data were imputed and thus all 509 subjects were included in the primary and secondary analyses. One participant was excluded from the cognition analysis after examining the quintiles of the residuals against the theoretical quintiles of a normal distribution. The mean CBCL T score was 46.9 (SD 9.5) (Table 1); using CBCL-specified cut-offs,[39] 449 (90.2%) of participants had a CBCL score in the normal range, 30 (6.0%) were borderline, and 19 (3.8%) scored in the clinical range. The mean Q-CHAT score was 30.5 (SD 9.3) (Table 1). The mean Bayley III Cognitive Composite score in our sample was 100 (SD 11.4) (Table 1), which corresponds to the standardised test mean;[42] 480 (94.3%) of participants had a normal cognitive score, 24 (4.7%) had mild impairment, 5 (1%) had moderate impairment, and nil had severe impairment. This distribution is not dissimilar from that of the normative sample.[42]

Association between maternal EPDS score and toddler CBCL and Q-CHAT scores

For peer review only - http://bmjopen.bmj.com/site/about/guidelines.xhtml

Predictors of children's CBCL and Q-CHAT scores after multiple imputation are shown in Table 2. Higher maternal EPDS score was associated with children's higher CBCL Total score (B=0.93, 95% CI 0.43-1.44, p<0.001, f^2=0.05) and Q-CHAT score (B=0.27, 95% CI 0.03-0.52, p=.031, f^2=0.01) (Table 2). These associations are presented graphically in Figure 1 and Figure 2, respectively. Boys had higher CBCL and Q-CHAT scores than girls. Higher Q-CHAT scores were associated with lower IMD rank (i.e., greater socio-economic deprivation) and lower Bayley-III cognitive composite scores. Parity was not a significant predictor of outcome in any of the models (Table 2).

Maternal EPDS score did not disproportionately affect preterm children with respect to CBCL or Q-CHAT scores (Table 3).

Association between maternal EPDS score and toddler CBCL internalising and externalising scores

Higher maternal EPDS score was associated with both internalising (B=0.22, 95% CI 0.08-0.36, p<0.01, f^2=0.03) and externalising (B=0.40, 95% CI 0.20-0.61, p<0.001, f^2=0.05) symptoms in children (Supplementary Table 1 and 2, respectively). Comparison of the imputed model analyses to the complete-case analyses showed that results were consistent for the CBCL model (Supplementary Table 3). Comparison for the Q-CHAT model showed that maternal EPDS was a significant predictor in the imputed model, but not in the complete-case analysis (Supplementary Table 3).

Effect of time-lag between baby's birth and mother's EPDS assessment and EPDS score

Mothers who gave birth prematurely (<37 weeks gestation) had their EPDS score assessed on average 7.7 weeks later post-delivery than mothers who gave birth at term (preterm participants M=8.9 (SD 4.8), term participants M=1.2 (SD 1.3); t(99.4)=15.5, p<.001). The time-lag between birth and EPDS assessment did not predict maternal EPDS score, and there was no evidence of a significant interaction between gestation and birth-to-assessment time-lag (Supplementary Table 4 and 5, respectively).

Discussion

Principal findings

Our results showed that more maternal self-reported depressive symptoms shortly after birth were associated with greater parent-reported toddlers' behavioural problems. Given that

For peer review only - http://bmjopen.bmj.com/site/about/guidelines.xhtml

fewer than 5% of the mothers in our cohort had EPDS scores above a clinical threshold,[37] our findings indicate that even subclinical depressive symptoms – i.e. not only diagnostic postnatal depression – adversely impact children's behavioural outcomes. In addition, our cohort was typically developing with few CBCL scores reaching a concerning threshold; our results could be interpreted within the conceptual framework of mental illness lying on a continuum with typical behavioural traits.[49] Our findings further showed that preterm birth did not influence the association between self-reported maternal depressive symptoms and parent-reported infants' behavioural outcomes in toddlerhood. This indicates that in this context preterm birth may not be regarded as a vulnerability or plasticity factor. Interestingly, mothers of preterm infants did not report more depressive symptoms compared to mothers of term infants in this study.

Comparison to prior literature

Our results with respect to internalising and externalising symptoms are in line with previous studies, including large population cohort studies, that showed an association between postnatal maternal depression and young children's emotional and behavioural problems.[11] Another previous study in 18-month old toddlers found that maternal depression was associated with internalising and dysregulated behaviour, but not externalising symptoms.[50] This difference between our and Conroy et al.'s findings may have arisen from their exclusion of infants born <36 weeks and their use of a clinical diagnosis of depression for mothers, rather than the continuous self-reported approach we employed. Interestingly, our finding that even subclinical depressive symptoms may adversely impact parent-reported child behavioural outcomes is in line with recent data showing that low- as well as high-level depressive symptoms are associated with internalising and externalising symptoms in children aged 3 years.[51]

The results showing an association between maternal postnatal depressive symptoms and the Q-CHAT are less robust and need to be interpreted with caution. Firstly, these results must be viewed in the context of the Q-CHAT having a low positive predictive value for autism, with the measure perhaps being more reflective of developmental immaturity. [41] Although some prior studies have shown an association between antenatal maternal depression and offspring's ASD,[10,52] and postnatal depression has been suggested as a potential focus of cross-domain studies of ASD,[53] there is no clear aetiological role of maternal postnatal depression in the development of ASD *per se*. Also, given that mothers with ASD are more

For peer review only - http://bmjopen.bmj.com/site/about/guidelines.xhtml

likely to suffer from perinatal depression than mothers without ASD,[54] and ASD is highly heritable,[55] maternal depression may actually be a confounding rather than causative factor in our observed results. Overall, therefore, our findings with respect to the Q-CHAT do not provide support for a role of maternal depression in the aetiology of autism traits, but rather suggest that maternal depression can influence toddler behaviour.

The finding that preterm infants were not disproportionately affected by maternal depressive symptoms supports Hadfield et al.'s findings that maternal distress at 9 months did not differentially impact very preterm (<34 weeks) or late preterm (34-36^{+6} weeks) infants with respect to socioemotional outcomes, although paternal distress did have an impact on very preterm infants' outcomes.[24] However, our results differ from Gueron-Sela et al.'s finding that very preterm (28-33 weeks) 12 month old infants' social outcomes were more influenced by maternal emotional distress at 6 months than term infants' outcomes.[23] The inconsistent findings may be due to methodological differences: for instance, our infant assessment being conducted at 18 months corrected age when social competency is more developed, our assessment of maternal depressive symptoms being in the very early postnatal period, or our use of the CBCL and Q-CHAT tools as markers of toddler behaviour. Importantly, the lack of support for a diathesis-stress or differential susceptibility model of maternal mental state on preterm infants in our study must be viewed in the context of our results also showing no difference in CBCL and Q-CHAT scores between term and preterm infants. This is in contrast to the existing literature that preterm infants are more likely than term infants to develop behavioural problems, such as ADHD, in childhood and adolescence.[20,33] It is possible that the phenotypes of neurodevelopmental and neuropsychiatric disorders assessed with the chosen behavioural measures may not be sufficiently expressed at 18 months corrected age.[56] In addition, as briefly discussed above, much of the existing literature emphasises the risk of extreme (<28 weeks) or very preterm (28-33 weeks) birth on later behavioural outcomes,[20,33] whereas only 3.5% and 5.5% of our participants fell within the extreme and very preterm group, respectively, and we thus may not have the power to show any subtle effects.

Strengths & limitations of the study

The strengths of this study lie primarily in its large sample and prospective data collection. Moreover, the use of multiple imputation methodology has facilitated retention of a complete

For peer review only - http://bmjopen.bmj.com/site/about/guidelines.xhtml

dataset, thus minimising non-response bias and increasing parameter precision. A strength in comparison to prior population cohort studies is that we assessed very early maternal depressive symptoms, and our sample is perhaps more representative of today's society – with increasing maternal age – than large cohort studies conducted in the 1990s-2000s. Given the complex interplay of biological and environmental factors in the aetiology of behavioural disorders, the inclusion of a substantive proportion of preterm infants in our cohort also offers an important insight into the role of preterm birth in behavioural outcomes; moreover, our results represent the full gestational spectrum, rather than discrete gestational categories. In addition, using maternal depressive symptoms as a continuous, rather than dichotomous, variable allows a more nuanced understanding of the role maternal postnatal depressive symptoms may play in influencing children's outcomes.

There are several limitations to this study that necessitate our findings to be considered with caution. Firstly, differences in birth-to-EPDS-assessment time-lags are a potential confounder, given the time-sensitive nature of early-onset temporary baby blues and later-onset pathological postnatal depression. Mothers of infants born at term were assessed early post-delivery, within the period one would anticipate baby blues to present, whereas mothers of preterm participants were on average assessed later, when postnatal depression predominates.[1,57] Although our post-hoc analyses showed that the time elapsed from birth to EPDS assessment was not associated with maternal EPDS score, providing reassurance that our assessments of mothers of term-born infants were not inflated by the common temporary symptoms of baby blues, it is possible that we did not capture the full extent of later-onset depressive symptoms in mothers of term-born infants. This may explain why maternal EPDS scores did not differ between preterm and term groups in our complete dataset analysis, contrary to the current literature,[31] as well as why our rate of postpartum depression, using an EPDS cut-off of 13, was low (4.1%) compared to the previously documented UK community prevalence rate of 8.9% at eight weeks postpartum.[58] Our results must therefore be interpreted with some caution.

Secondly, although statistical techniques were used to impute missing data and mitigate this problem, 14.3% of maternal EPDS scores were missing. This rate of missingness may relate to some mothers being reluctant to complete a questionnaire at the time their child is having an MRI, or due to simultaneous childcare duties. Thirdly, a number of important confounders that are likely to affect children's behavioural outcomes were not assessed in this study,

For peer review only - http://bmjopen.bmj.com/site/about/guidelines.xhtml

including genetic risk for psychiatric disorders,[59] parental psychiatric co-morbidities,[50] chronicity of postnatal depressive symptoms,[51] antenatal maternal depression, paternal depression and subsequent parent-infant attachment, and inter-parental conflict.[11] Thus, we are unable to conclude whether our observed associations between early postnatal maternal depressive symptoms and children's behavioural outcomes are moderated or mediated by other parental and/or psychiatric factors.

Fourthly, whilst our study included a reasonable proportion of preterm infants (97/509, 19%), our sample was not random, as preterm children were selectively recruited for the DHCP; indeed, preterm infants are over-represented in our sample when compared to the UK population incidence (7.3%),[60] which may limit the study's generalisability to the general population. This over-representation of preterm infants may explain why our mean maternal age is higher than the national mean age of 30.7,[61] given that increasing maternal age is associated with increased risk of adverse pregnancy outcomes.[62] Our observed large maternal age range in itself also poses a limitation on the generalisability of our findings to the general population, and further research would be necessary to identify a possible moderation effect of high maternal age on both EPDS scores and child behavioural outcomes. Furthermore, although a 19% prevalence of preterm birth is high for a community sample, the proportion of very and extreme preterm infants in our sample is small, and this may not have provided sufficient power to detect any differential susceptibility effect of preterm birth on outcomes.

Sixthly, the effect sizes of the association between maternal EPDS score and behavioural problems were small; this raises questions regarding the clinical significance of our findings and potentially explains some of the inconsistency between this and previous studies. Even within our analyses, the association between maternal depressive symptoms and Q-CHAT scores was not observed in our complete case analysis, thus calling into question the validity of this result. It is also important to highlight again the poor positive predictive value of the Q-CHAT for autism;[41] higher Q-CHAT scores do not imply a diagnosis of ASD, and this distinction may also explain the contrast to previous studies.

Finally, it is well documented that maternal depression influences reporting of Q-CHAT [63] and CBCL scores.[64] Our study used maternal report of maternal depressive symptoms, and our outcome measures were parent-completed questionnaires; despite the CBCL showing

For peer review only - http://bmjopen.bmj.com/site/about/guidelines.xhtml

good cross-informant agreement,[39] it is thus possible that reporting bias with common method variance could have skewed our results.

Implications of our findings

Of greatest importance to clinicians and policymakers is our finding that even *subclinical* self-reported maternal depressive symptoms are associated with parent-reported behavioural outcomes of offspring. This has significant implications for the risk-stratification of women and their babies in the postnatal period, during which contact with medical professionals is already established. Identifying high risk families and providing appropriate supportive measures at the early postnatal stage may help to prevent future psychiatric morbidity.

Future research

Further follow-up of large cohorts of preterm and term infants, to an age when behavioural phenotypes may become more pronounced, is needed to investigate whether the long-term developmental trajectories of term and ex-preterm infants are differentially susceptible to changes of postnatal maternal mental health. Future research should consider both maternal and paternal mental health, as well as socioeconomic and environmental factors on child outcomes. Such follow-up should use independent, objective assessments of child behavioural outcomes in order to avoid the common method variance inherent to parent-reported measures. Finally, it is crucial for future research to elucidate the interplay of biochemical and neurodevelopmental changes that may mediate and confound the translation of environmental exposures into distal behavioural phenotypes.

Conclusion

This prospective longitudinal cohort study found no evidence to support the concept of preterm birth as a vulnerability or plasticity factor with respect to the effect of maternal depressive symptoms on behavioural development. However, we showed that early subclinical maternal postnatal depressive symptoms were associated with behavioural problems in children on parent-reported measures. This adds to the increasing body of literature indicating the role of subclinical and early postnatal depressive symptoms in the aetiology of childhood behavioural disorders. These findings are of great relevance to child and public health, and further research may strengthen its implications for developing strategies to facilitate effective screening and support for women and children, enabling all to reach their full potential.

For peer review only - http://bmjopen.bmj.com/site/about/guidelines.xhtml

Acknowledgements & contributions

We thank all DHCP investigators for their contribution to the study. We thank Dr Oliver Gale-Grant MRes (Centre for the Developing Brain, King's College London; Department of Forensic & Neurodevelopmental Sciences, King's College London) for providing the IMD rank data. We are very grateful to the families who generously took part in this research.

Conceptualization: SC, ADE, CN; **Methodology:** IK, GV, SC, AP, ADE, CN; **Investigation:** SF, AC; **Data curation:** IK, AL; **Formal analysis:** IK, GV, AP, CN; **Writing – original draft preparation:** IK; **Writing – Review & Editing:** GV, AL, SF, AC, SC, AP, ADE, CN; **Visualisation:** IK, GV; **Funding acquisition:** SC, ADE; **Supervision:** AP, ADE, CN.

References

1. Woody CA, Ferrari AJ, Siskind DJ, Whiteford HA, Harris MG. A systematic review and meta-regression of the prevalence and incidence of perinatal depression. *J Affect Disord*. 2017;219:86-92. doi:10.1016/j.jad.2017.05.003

2. Rezaie-Keikhaie K, Arbabshastan ME, Rafiemanesh H, Amirshahi M, Ostadkelayeh SM, Arbabisarjou A. Systematic Review and Meta-Analysis of the Prevalence of the Maternity Blues in the Postpartum Period. *J Obstet Gynecol Neonatal Nurs*. 2020;49(2):127-136. doi:10.1016/j.jogn.2020.01.001

3. Beck CT. Predictors of postpartum depression: an update. *Nurs Res*. 2001;50(5):275-285.

4. de Paula Eduardo JAF, de Rezende MG, Menezes PR, Del-Ben CM. Preterm birth as a risk factor for postpartum depression: A systematic review and meta-analysis. *J Affect Disord*. 2019;259:392-403. doi:10.1016/j.jad.2019.08.069

5. Blom EA, Jansen PW, Verhulst FC, et al. Perinatal complications increase the risk of postpartum depression. The Generation R Study. *BJOG Int J Obstet Gynaecol*. 2010;117(11):1390-1398. doi:10.1111/j.1471-0528.2010.02660.x

6. Beck CT. Postpartum depression: a metasynthesis. *Qual Health Res*. 2002;12(4):453-472. doi:10.1177/104973202129120016

7. Grace SL, Evindar A, Stewart DE. The effect of postpartum depression on child cognitive development and behavior: a review and critical analysis of the literature. *Arch Womens Ment Health*. 2003;6(4):263-274. doi:10.1007/s00737-003-0024-6

8. Lebel C, Walton M, Letourneau N, Giesbrecht GF, Kaplan BJ, Dewey D. Prepartum and postpartum maternal depressive symptoms are related to children's brain structure in preschool. *Biol Psychiatry*. 2016;80(11):859-868. doi:10.1016/j.biopsych.2015.12.004

For peer review only - http://bmjopen.bmj.com/site/about/guidelines.xhtml

9. Goodman SH, Rouse MH, Connell AM, Broth MR, Hall CM, Heyward D. Maternal depression and child psychopathology: a meta-analytic review. *Clin Child Fam Psychol Rev*. 2011;14(1):1-27. doi:10.1007/s10567-010-0080-1

10. Hagberg KW, Robijn AL, Jick S. Maternal depression and antidepressant use during pregnancy and the risk of autism spectrum disorder in offspring. *Clin Epidemiol*. 2018;10:1599-1612. doi:10.2147/CLEP.S180618

11. Stein A, Pearson RM, Goodman SH, et al. Effects of perinatal mental disorders on the fetus and child. *The Lancet*. 2014;384(9956):1800-1819. doi:10.1016/S0140-6736(14)61277-0

12. Van Batenburg-Eddes T, Brion MJ, Henrichs J, et al. Parental depressive and anxiety symptoms during pregnancy and attention problems in children: a cross-cohort consistency study. *J Child Psychol Psychiatry*. 2013;54(5):591-600. doi:10.1111/jcpp.12023

13. Barker ED, Jaffee SR, Uher R, Maughan B. The contribution of prenatal and postnatal maternal anxiety and depression to child maladjustment. *Depress Anxiety*. 2011;28(8):696-702. doi:10.1002/da.20856

14. Pearson RM, Evans J, Kounali D, et al. Maternal depression during pregnancy and the postnatal period: risks and possible mechanisms for offspring depression at 18 years. *JAMA Psychiatry*. 2013;70(12):1312-1319. doi:10.1001/jamapsychiatry.2013.2163

15. Kleine I, Falconer S, Roth S, et al. Early postnatal maternal trait anxiety is associated with the behavioural outcomes of children born preterm <33 weeks. *J Psychiatr Res*. 2020;131:160-168. doi:10.1016/j.jpsychires.2020.09.010

16. Feder A, Nestler EJ, Charney DS. Psychobiology and molecular genetics of resilience. *Nat Rev Neurosci*. 2009;10(6):446-457. doi:10.1038/nrn2649

17. World Health Organization. Preterm birth. Published February 19, 2018. Accessed May 18, 2022. https://www.who.int/news-room/fact-sheets/detail/preterm-birth

18. Dimitrova R, Pietsch M, Christiaens D, et al. Heterogeneity in brain microstructural development following preterm birth. *Cereb Cortex*. 2020;30(9):4800-4810. doi:10.1093/cercor/bhaa069

19. Fitzallen GC, Sagar YK, Taylor HG, Bora S. Anxiety and depressive disorders in children born preterm: a meta-analysis. *J Dev Behav Pediatr JDBP*. 2021;42(2):154-162. doi:10.1097/DBP.0000000000000898

20. Allotey J, Zamora J, Cheong-See F, et al. Cognitive, motor, behavioural and academic performances of children born preterm: a meta-analysis and systematic review involving 64 061 children. *BJOG Int J Obstet Gynaecol*. 2018;125(1):16-25. doi:10.1111/1471-0528.14832

21. Jaekel J, Pluess M, Belsky J, Wolke D. Effects of maternal sensitivity on low birth weight children's academic achievement: a test of differential susceptibility versus diathesis stress. *J Child Psychol Psychiatry*. 2015;56(6):693-701. doi:10.1111/jcpp.12331

22. Belsky J, Bakermans-Kranenburg MJ, van IJzendoorn MH. For better and for worse: differential susceptibility to environmental influences. *Curr Dir Psychol Sci*. 2007;16(6):300-304. doi:10.1111/j.1467-8721.2007.00525.x

For peer review only

23. Gueron-Sela N, Atzaba-Poria N, Meiri G, Marks K. The caregiving environment and developmental outcomes of preterm infants: diathesis stress or differential susceptibility effects? *Child Dev*. 2015;86(4):1014-1030. doi:https://doi.org/10.1111/cdev.12359

24. Hadfield K, O'Brien F, Gerow A. Is level of prematurity a risk/plasticity factor at three years of age? *Infant Behav Dev*. 2017;47:27-39. doi:10.1016/j.infbeh.2017.03.003

25. Belsky J, Pluess M. Beyond diathesis stress: differential susceptibility to environmental influences. *Psychol Bull*. 2009;135(6):885-908. doi:10.1037/a0017376

26. Pluess M, Belsky J. Prenatal programming of postnatal plasticity? *Dev Psychopathol*. 2011;23(1):29-38. doi:10.1017/S0954579410000623

27. DeMaster D, Bick J, Johnson U, Montroy JJ, Landry S, Duncan AF. Nurturing the preterm infant brain: leveraging neuroplasticity to improve neurobehavioral outcomes. *Pediatr Res*. 2019;85(2):166-175. doi:10.1038/s41390-018-0203-9

28. World Health Organization (WHO). Global nutrition targets 2025: low birth weight policy brief. Published online 2014.

29. Kramer MS. Determinants of low birth weight: methodological assessment and meta-analysis. *Bull World Health Organ*. 1987;65(5):663-737.

30. Goldenberg RL, Culhane JF, Iams JD, Romero R. Epidemiology and causes of preterm birth. *Lancet Lond Engl*. 2008;371(9606):75-84. doi:10.1016/S0140-6736(08)60074-4

31. Carson C, Redshaw M, Gray R, Quigley MA. Risk of psychological distress in parents of preterm children in the first year: evidence from the UK Millennium Cohort Study. *BMJ Open*. 2015;5(12):e007942. doi:10.1136/bmjopen-2015-007942

32. Vigod SN, Villegas L, Dennis CL, Ross LE. Prevalence and risk factors for postpartum depression among women with preterm and low-birth-weight infants: a systematic review. *BJOG Int J Obstet Gynaecol*. 2010;117(5):540-550. doi:10.1111/j.1471-0528.2009.02493.x

33. Johnson S, Wolke D. Behavioural outcomes and psychopathology during adolescence. *Early Hum Dev*. 2013;89(4):199-207. doi:10.1016/j.earlhumdev.2013.01.014

34. Lautarescu A, Victor S, Lau-Zhu A, Counsell SJ, Edwards AD, Craig MC. The factor structure of the Edinburgh Postnatal Depression Scale among perinatal high-risk and community samples in London. *Arch Womens Ment Health*. 2022;25(1):157-169. doi:10.1007/s00737-021-01153-0

35. Ministry of Housing, Communities & Local Government. The English indices of deprivation 2019. Published online 2019. https://assets.publishing.service.gov.uk/government/uploads/system/uploads/attachment_data/file/835115/IoD2019_Statistical_Release.pdf

36. Boyd A, Golding J, Macleod J, et al. Cohort Profile: The 'Children of the 90s'—the index offspring of the Avon Longitudinal Study of Parents and Children. *Int J Epidemiol*. 2013;42(1):111-127. doi:10.1093/ije/dys064

37. Cox JL, Holden JM, Sagovsky R. Detection of postnatal depression: development of the 10-item Edinburgh Postnatal Depression Scale. *Br J Psychiatry*. 1987;150(6):782-786. doi:10.1192/bjp.150.6.782

For peer review only - http://bmjopen.bmj.com/site/about/guidelines.xhtml

38. Levis B, Negeri Z, Sun Y, Benedetti A, Thombs BD. Accuracy of the Edinburgh Postnatal Depression Scale (EPDS) for screening to detect major depression among pregnant and postpartum women: systematic review and meta-analysis of individual participant data. *BMJ.* 2020;371. doi:10.1136/bmj.m4022

39. Achenbach TM, Rescorla LA. *Manual for the ASEBA Preschool Forms & Profiles*.; 2000.

40. Allison C, Baron-Cohen S, Wheelwright S, et al. The Q-CHAT (Quantitative Checklist for Autism in Toddlers): a normally distributed quantitative measure of autistic traits at 18-24 months of age: preliminary report. *J Autism Dev Disord*. 2008;38(8):1414-1425. doi:10.1007/s10803-007-0509-7

41. Allison C, Matthews FE, Ruta L, et al. Quantitative Checklist for Autism in Toddlers (Q-CHAT). A population screening study with follow-up: the case for multiple time-point screening for autism. *BMJ Paediatr Open*. 2021;5(1):e000700. doi:10.1136/bmjpo-2020-000700

42. Bayley N. *Bayley Scales of Infant and Toddler Development - Third Edition*. 3rd ed. Harcourt Assessment; 2006.

43. Albers CA, Grieve AJ. Review of Bayley scales of infant and toddler development-third edition. *J Psychoeduc Assess*. 2007;25(2):180-190. doi:10.1177/0734282906297199

44. Anderson PJ, De Luca CR, Hutchinson E, Roberts G, Doyle LW, the Victorian Infant Collaborative Group. Underestimation of developmental delay by the new Bayley-III scale. *Arch Pediatr Adolesc Med*. 2010;164(4):352-356. doi:10.1001/archpediatrics.2010.20

45. Rubin DB. Inference and missing data. *Biometrika*. 1976;63(3):581-592. doi:10.2307/2335739

46. Cohen J. *Statistical Power Analysis for the Behavioral Sciences*. 2nd ed. L. Erlbaum Associates; 1988.

47. Selya AS, Rose JS, Dierker LC, Hedeker D, Mermelstein RJ. A practical guide to calculating Cohen's f2, a measure of local effect size, from PROC MIXED. *Front Psychol*. 2012;3. doi:10.3389/fpsyg.2012.00111

48. Harel O. The estimation of R 2 and adjusted R 2 in incomplete data sets using multiple imputation. *J Appl Stat*. 2009;36(10):1109-1118. doi:10.1080/02664760802553000

49. Plomin R, Haworth CMA, Davis OSP. Common disorders are quantitative traits. *Nat Rev Genet*. 2009;10(12):872-878. doi:10.1038/nrg2670

50. Conroy S, Pariante CM, Marks MN, et al. Maternal psychopathology and infant development at 18 months: the impact of maternal personality disorder and depression. *J Am Acad Child Adolesc Psychiatry*. 2012;51(1):51-61. doi:10.1016/j.jaac.2011.10.007

51. Kingston D, Kehler H, Austin MP, et al. Trajectories of maternal depressive symptoms during pregnancy and the first 12 months postpartum and child externalizing and internalizing behavior at three years. *PloS One*. 2018;13(4):e0195365. doi:10.1371/journal.pone.0195365

52. Wiggins LD, Rubenstein E, Daniels J, et al. A phenotype of childhood autism is associated with preexisting maternal anxiety and depression. *J Abnorm Child Psychol*. 2019;47(4):731-740. doi:10.1007/s10802-018-0469-8

For peer review only - http://bmjopen.bmj.com/site/about/guidelines.xhtml

53. Babb JA, Deligiannidis KM, Murgatroyd CA, Nephew BC. Peripartum depression and anxiety as an integrative cross-domain target for psychiatric preventative measures. *Behav Brain Res*. 2015;0:32-44. doi:10.1016/j.bbr.2014.03.039

54. Pohl AL, Crockford SK, Blakemore M, Allison C, Baron-Cohen S. A comparative study of autistic and non-autistic women's experience of motherhood. *Mol Autism*. 2020;11(1):3. doi:10.1186/s13229-019-0304-2

55. Colvert E, Tick B, McEwen F, et al. Heritability of autism spectrum disorder in a UK population-based twin sample. *JAMA Psychiatry*. 2015;72(5):415-423. doi:10.1001/jamapsychiatry.2014.3028

56. Egger HL, Emde RN. Developmentally sensitive diagnostic criteria for mental health disorders in early childhood: the diagnostic and statistical manual of mental disorders-IV, the research diagnostic criteria-preschool age, and the diagnostic classification of mental health and developmental disorders of infancy and early childhood-revised. *Am Psychol*. 2011;66(2):95-106. doi:10.1037/a0021026

57. Henshaw C. Mood disturbance in the early puerperium: a review. *Arch Women's Ment Health*. 2003;6(2):s33-s42. doi:10.1007/s00737-003-0004-x

58. Heron J, O'Connor TG, Evans J, Golding J, Glover V. The course of anxiety and depression through pregnancy and the postpartum in a community sample. *J Affect Disord*. 2004;80(1):65-73. doi:10.1016/j.jad.2003.08.004

59. Biel MG. Parental psychiatric symptoms and children's outcomes: toward understanding and responding to intergenerational risk in child psychiatry. *J Am Acad Child Adolesc Psychiatry*. 2018;57(9):632-633. doi:10.1016/j.jaac.2018.06.010

60. National Institute for Health and Care Excellence. Preterm labour and birth (NICE guideline 25). Published online November 2015. Accessed January 27, 2019. https://www.nice.org.uk/guidance/ng25

61. Office for National Statistics. Birth characteristics in England and Wales: 2020. Published January 13, 2022. Accessed February 5, 2022. https://www.ons.gov.uk/peoplepopulationandcommunity/birthsdeathsandmarriages/livebirths/bulletins/birthcharacteristicsinenglandandwales/2020#birth-characteristics

62. Londero AP, Rossetti E, Pittini C, Cagnacci A, Driul L. Maternal age and the risk of adverse pregnancy outcomes: a retrospective cohort study. *BMC Pregnancy Childbirth*. 2019;19(1):261. doi:10.1186/s12884-019-2400-x

63. Goh DA, Gan D, Kung J, et al. Child, maternal and demographic factors influencing caregiver-reported autistic trait symptomatology in toddlers. *J Autism Dev Disord*. 2018;48(4):1325-1337. doi:10.1007/s10803-018-3471-7

64. Friedlander S, Weiss DS, Traylor J. Assessing the influence of maternal depression on the validity of the Child Behavior Checklist. *J Abnorm Child Psychol*. 1986;14(1):123-133. doi:10.1007/bf00917228

For peer review only

Table 1: Socio-demographic, maternal and clinical characteristics (n=509)

Variable	Number (%)*
Sex: Male	274 (53.8)
Index of Multiple Deprivation (IMD) quintiles	
1 (least deprived) [a]	65 (12.8)
2	87 (17.2)
3	108 (21.3)
4	173 (34.2)
5 (most deprived)	73 (14.4)
Gestational age at birth (weeks), median [range]	39.7 [20 – 43]
Gestational category	
Extremely preterm (<28 weeks)	18 (3.5)
Very preterm (28-32 weeks)	28 (5.5)
Late preterm (32-37 weeks)	51 (10.0)
Term (≥37 weeks)	412 (80.9)
Birthweight (g), median [range]	3290 [450 – 4750]
Multiple pregnancy	54 (10.6)
Maternal parity	
0	332 (65.2)
1	124 (24.4)
2	32 (6.3)
3+	21 (4.2)
Maternal BMI (kg/m^2), median [range]	23.2 [15.3 – 43.6]
Maternal age at infant's birth (years), mean (SD) [range]	34.2 (4.8)<br>[17 – 52]
Maternal ethnicity	
White	272 (53.4)
Black/Black British	56 (11.0)
Asian/Asian British	28 (5.5)
Chinese	18 (3.5)
Mixed – White & Asian	4 (0.8)
Mixed – White & Black	4 (0.8)
Any other	30 (5.9)
Do not wish to answer	9 (1.8)
No data	88 (17.3)
Bayley III cognitive composite score, mean (SD) [range]	100 (11.4)<br>[55 – 125]
CBCL total T score, mean (SD) [range]	46.9 (9.5)<br>[28 – 69]
Q-CHAT total score, mean (SD) [range]	30.5 (9.3)<br>[8 – 70]
EPDS score, median [range]	4 [0 – 28]
EPDS score, n (%)	
<13	415 (8.2)
≥13	21 (4.1)
No data	73 (14.3)

[a] Quintile 1 corresponds to the highest, least deprived, IMD rankings.

* unless otherwise specified

For peer review only

Table 2: CBCL and Q-CHAT model predictors using multiple imputation without interaction. (Cf. Supplementary Table 3 for complete case analysis)

	CBCL			Q-CHAT		
	**B [95%CI]**	**p**	**f^2**	**B [95%CI]**	**p**	**f^2**
**Maternal EPDS**	0.93 [0.43, 1.44]	<.001 ***	0.05	0.27 [0.03, 0.52]	.031 *	0.01
**Maternal BMI**	-0.09 [-0.44, 0.26]	.621	-	0.06 [-0.13, 0.24]	.538	-
**Multiple pregnancy**	3.15 [-3.07, 9.37]	.320	-	1.33 [-2.62, 5.28]	.509	-
**Parity**						
**1**	-2.52 [-5.96, 0.93]	.151	-	-2.14 [-4.02, -0.27]	.025 [a]	-
**2**	-3.23 [-9.16, 2.70]	.285	-	0.88 [-1.99, 3.75]	.548	-
**3+**	-1.37 [-8.36, 5.61]	.699	-	-0.49 [-4.57, 3.60]	.815	-
**IMD rank**	-1.48 [-3.33, 0.37]	.117	-	-1.50 [-2.60, -0.40]	.008 **	0.02
**Gestational age at birth (weeks)**	0.10 [-0.65, 0.85]	.786	-	0.26 [-0.17, 0.70]	.233	-
**Birthweight (kg)**	0.56 [-2.65, 3.78]	.731	-	-1.24 [-2.93, 0.46]	.151	-
**Sex:female**	-4.14 [-6.96, -1.31]	.004 **	0.06	-1.95 [-3.42, -0.48]	.009 **	0.05
**Corrected age at assessment (months)**	-0.90 [-2.17, 0.37]	.166	-	-0.16 [-0.91, 0.59]	.677	-
**Cognition**	-0.05 [-0.20, 0.09]	.467	-	-0.27 [-0.35, -0.20]	<.001 ***	0.12

p<0.05 *; p<0.01 **; p<0.001 ***
CBCL model adjusted R^2 = 0.0676. Q-CHAT model adjusted R^2 = 0.193.
B = unstandardised coefficient.
CBCL = Child Behaviour Checklist score at 18 months. Q-CHAT = Quantitative Checklist for Autism in Toddlers score at 18 months. Maternal EPDS = maternal Edinburgh Postnatal Depression Scale score at term-equivalent age. Multiple pregnancy = dummy variable of twin/triplet pregnancy. Parity = dummy variable, one/two/three+ previous child(ren). Corrected age at assessment (months) = age at behavioural assessment, corrected for gestational age. Cognition = infant Bayley III score at 18 months.
[a] Wald test of whole parity variable in Q-CHAT model: F(3, 476.9) = 1.88, p = .133
Effect size (Cohen's f^2, calculated from squared part correlations for predictors significant to 0.05): 0.02 = small, 0.15 = medium and 0.35 = large.[46]
- indicates data not given, as predictor not significant to 0.05.

For peer review only - http://bmjopen.bmj.com/site/about/guidelines.xhtml

Table 3: CBCL and Q-CHAT model predictors using complete case analysis with interaction of 'EPDS x term'.

	CBCL		Q-CHAT	
	**B [95%CI]**	**p**	**B [95%CI]**	**p**
**Maternal EPDS**	0.89 [-0.24, 2.02]	.121	0.24 [-0.28, 0.75]	.365
**Maternal BMI**	-0.01 [-0.39, 0.37]	.955	0.00 [-0.16, 0.17]	.982
**Multiple pregnancy**	1.76 [-6.65, 10.17]	.681	0.97 [-2.07, 4.01]	.532
**Parity**				
**1**	-2.75 [-6.49, 0.99]	.149	-1.42 [-3.30, 0.46]	.139
**2**	-3.49 [-10.36, 3.37]	.317	0.16 [-2.84, 3.16]	.917
**3+**	-1.17 [-9.69, 7.35]	.788	-1.13 [-4.24, 1.98]	.476
**IMD rank**	-1.41 [-3.54, 0.73]	.195	-1.68 [-2.64, -0.72]	.001 **
**Gestation: term**	1.25 [-8.34, 10.85]	.797	2.64 [-1.74, 7.02]	.236
**Birthweight (kg)**	-1.01 [-4.08, 2.05]	.516	-2.25 [-3.73, -0.78]	.003 **
**Sex: female**	-4.64 [-7.83, -1.44]	.005 **	-2.22 [-3.72, -0.71]	.004 **
**Corrected age at assessment (months)**	-0.83 [-2.27, 0.62]	.261	-0.39 [-1.18, 0.04]	.335
**Cognition**	-0.03 [-0.20, 0.14]	.720	-0.22 [-0.29, -.0.15]	<.001 ***
**EPDS x gestation:term**	-0.01 [-1.30, 1.28]	.991	-0.02 [-0.60, 0.56]	.950

p<0.05 *; p<0.01 **; p<0.001 ***
CBCL model adjusted $R^2 = 0.0865$. Q-CHAT model adjusted $R^2 = 0.215$.
B = unstandardised coefficient.
CBCL = Child Behaviour Checklist score at 18 months. Q-CHAT = Quantitative Checklist for Autism in Toddlers score at 18 months. Maternal EPDS = maternal Edinburgh Postnatal Depression Scale score at term-equivalent age. Multiple pregnancy = dummy variable of twin/triplet pregnancy. Parity = dummy variable, one/two/three+ previous child(ren). Gestation: term = dummy variable, term (≥37 weeks) vs preterm (<37 weeks) gestation at birth. Corrected age at assessment (months) = age at behavioural assessment, corrected for gestational age. Cognition = infant Bayley III score at 18 months. EPDS x gestation:term = interaction term between maternal EPDS score and term gestation at birth.

For peer review only

Fig.1 Children's predicted CBCL scores at 18 months are positively correlated to the maternal EPDS score at term-equivalent age.

Fig.2 Children's predicted Q-CHAT scores at 18 months are positively correlated to the maternal EPDS score at term-equivalent age.

For peer review only

For peer review only - http://bmjopen.bmj.com/site/about/guidelines.xhtml

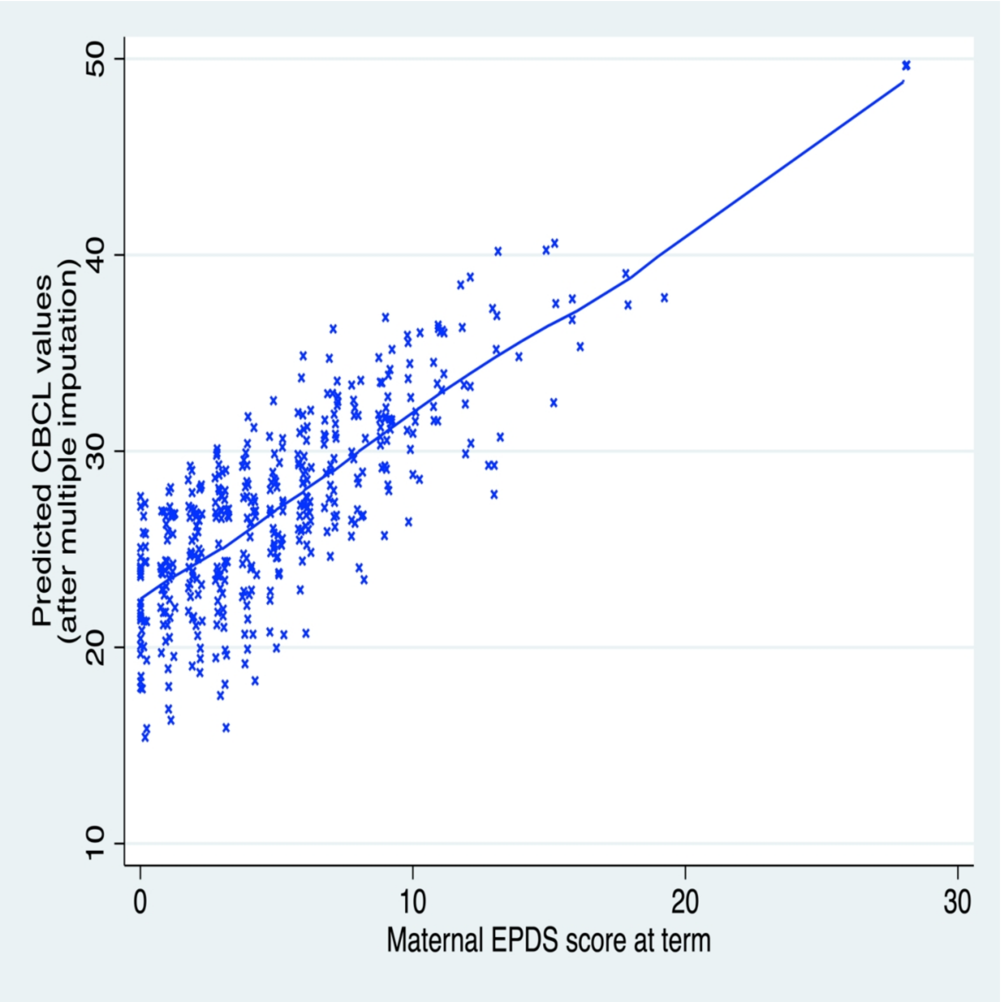

Fig.1 Children's predicted CBCL scores at 18 months are positively correlated to the maternal EPDS score at term-equivalent age

158x158mm (220 x 220 DPI)

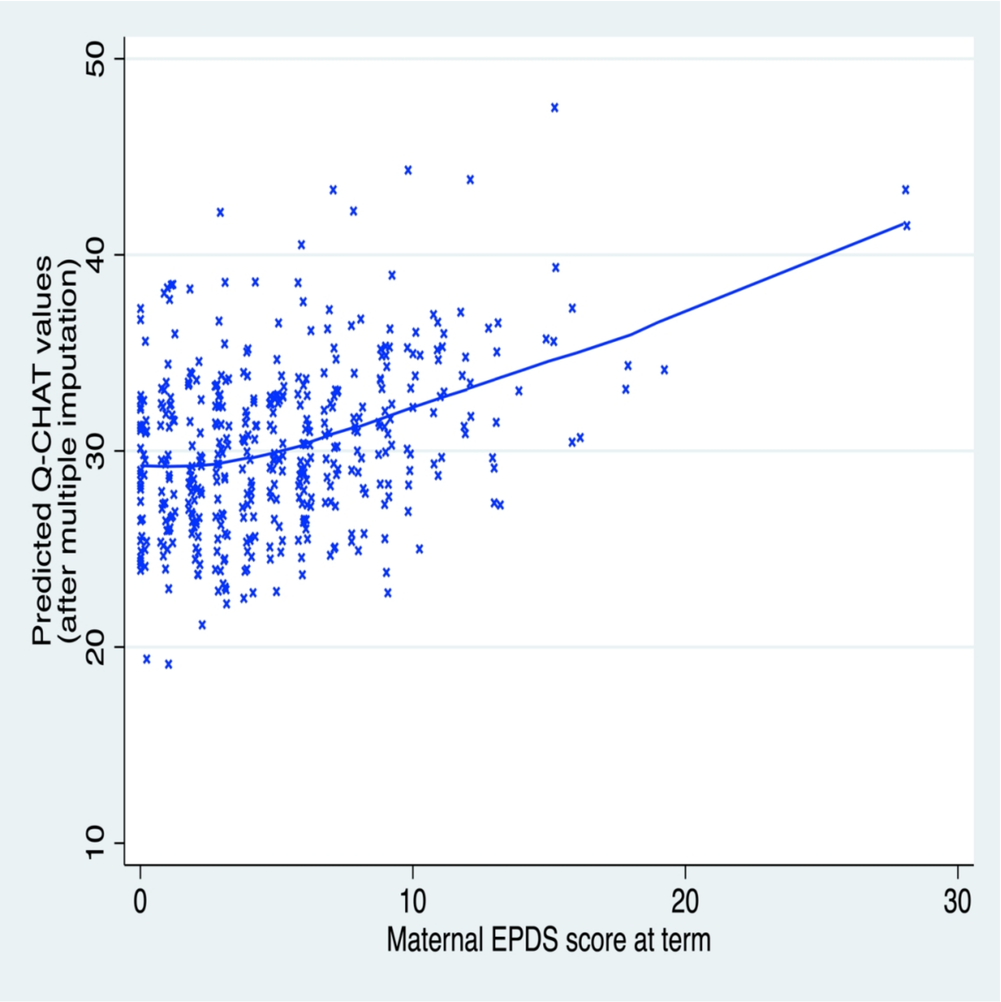

Fig.2 Children's predicted Q-CHAT scores at 18 months are positively correlated to the maternal EPDS score at term-equivalent age.

158x158mm (220 x 220 DPI)

Supplemental material

Supplementary Table 1: CBCL internalising symptom model predictors using multiple imputation.

Supplementary Table 2: CBCL externalising symptom model predictors using multiple imputation.

Supplementary Table 3: CBCL and Q-CHAT model predictors using complete case analysis without interaction.

Supplementary Table 4: EPDS score predictors.

Supplementary Table 5: EPDS score predictors including interaction 'term x time-lag'.

Supplementary Figure 1: Histogram showing the distribution of maternal EPDS scores at term-equivalent age.

For peer review only

Supplementary Table 1: CBCL internalising symptom model predictors using multiple imputation**.**

	B [95%CI]	p	f^2
**Maternal EPDS**	0.22 [0.08, 0.36]	.003 **	0.03
**Maternal BMI**	-0.04 [-0.13, 0.06]	.436	-
**Multiple pregnancy**	0.58 [-1.10, 2.27]	.497	-
**Parity**			
**1**	-0.37 [-1.41, 0.67]	.487	-
**2**	-1.33 [-3.09, 0.42]	.136	-
**3+**	0.64 [-1.34, 2.62]	.524	-
**IMD rank**	-0.41 [-0.91, 0.10]	.115	-
**Gestational age at birth (weeks)**	0.22 [-0.00, 0.44]	.053	-
**Birthweight (kg)**	-0.97 [-1.90, -0.05]	.038 *	0.005
**Sex: female**	-0.51 [-1.35, 0.33]	.232	-
**Corrected age at assessment (months)**	0.02 [-0.41, 0.45]	.923	-
**Cognition**	-0.05 [-0.10, -0.01]	.016 *	0.01

p<0.05 *; p<0.01 **; p<0.001 ***
Adjusted R^2 = 0.0566.
B = unstandardised coefficient.
Outcome variable = Child Behaviour Checklist internalising sub-score at 18 months. Maternal EPDS = maternal Edinburgh Postnatal Depression Scale score at term-equivalent age. Multiple pregnancy = dummy variable of twin/triplet pregnancy. Parity = dummy variable, one/two/three+ previous child(ren). Corrected age at assessment (months) = age at behavioural assessment, corrected for gestational age. Cognition = infant Bayley III score at 18 months.
Effect size (Cohen's f^2, calculated from squared part correlations for predictors significant to 0.05): 0.02 = small, 0.15 = medium and 0.35 = large.[1]
- indicates data not given, as predictor not significant to 0.05.

For peer review only

Supplementary Table 2: CBCL externalising symptom model predictors using multiple imputation.

	B [95%CI]	p	f^2
**Maternal EPDS**	0.40 [0.20, 0.61]	<.001 ***	0.05
**Maternal BMI**	0.01 [-0.17, 0.15]	.933	-
**Multiple pregnancy**	2.51 [-0.29, 5.31]	.079	-
**Parity**			
**1**	-1.06 [-2.53, 0.42]	.160	-
**2**	-0.61 [-3.55, 2.33]	.682	-
**3+**	-0.96 [-4.47, 2.56]	.593	-
**IMD rank**	-0.24 [-1.11, 0.63]	.585	-
**Gestational age at birth (weeks)**	-0.07 [-0.40, 0.27]	.701	-
**Birthweight (kg)**	1.03 [-0.38, 2.44]	.153	-
**Sex: female**	-1.80 [-3.07, -0.53]	.006 **	0.06
**Corrected age at assessment (months)**	-0.40 [-0.95, 0.16]	.161	-
**Cognition**	0.03 [-0.03, 0.10]	.322	-

p<0.05 *; p<0.01 **; p<0.001 ***
Adjusted R^2 = 0.0612.
B = unstandardised coefficient.
Outcome variable = Child Behaviour Checklist externalising sub-score at 18 months. Maternal EPDS = maternal Edinburgh Postnatal Depression Scale score at term-equivalent age. Multiple pregnancy = dummy variable of twin/triplet pregnancy. Parity = dummy variable, one/two/three+ previous child(ren). Corrected age at assessment (months) = age at behavioural assessment, corrected for gestational age. Cognition = infant Bayley III score at 18 months.
Effect size (Cohen's f^2, calculated from squared part correlations for predictors significant to 0.05): 0.02 = small, 0.15 = medium and 0.35 = large.[1]
- indicates data not given, as predictor not significant to 0.05.

For peer review only

Supplementary Table 3: CBCL and Q-CHAT model predictors using complete case analysis without interaction.

	CBCL		Q-CHAT	
	**B [95%CI]**	**p**	**B [95%CI]**	**p**
**Maternal EPDS**	0.88 [0.35, 1.41]	.001 **	0.21 [-0.02, 0.44]	.069
**Maternal BMI**	-0.01 [-0.38, 0.37]	.963	0.01 [-0.16, 0.17]	.930
**Multiple pregnancy**	1.50 [-6.87, 9.87]	.724	0.43 [-2.50, 3.37]	.772
**Parity**				
**1**	-2.83 [-6.60, 0.94]	.141	-1.54 [-3.41, 0.34]	.108
**2**	-3.49 [-10.3, 3.35]	.316	0.13 [-2.85, 3.10]	.933
**3+**	-1.38 [-10.0, 7.30]	.755	-1.43 [-4.50, 1.64]	.360
**IMD rank**	-1.44 [-3.56, 0.67]	.181	-1.75 [-2.70, -0.79]	<.001 ***
**Gestational age at birth (weeks)**	0.01 [-0.90, 0.91]	.987	0.10 [-0.33, 0.54]	.639
**Birthweight (kg)**	-0.65 [-4.38, 3.08]	.733	-1.81 [-3.63, 0.00]	.050
**Sex: female**	-4.57 [-7.82, -1.31]	.006 **	-2.12 [-3.60, -0.64]	.005 **
**Corrected age at assessment (months)**	-0.84 [-2.26, 0.59]	.247	-0.41 [-1.18, 0.35]	.290
**Cognition**	-0.03 [-0.20, 0.13]	.689	-0.23 [-0.30, -0.15]	<.001 ***

$p<0.05$ *; $p<0.01$ **; $p<0.001$ ***
CBCL adjusted $R^2 = 0.0862$. Q-CHAT adjusted $R^2 = 0.2103$.
B = unstandardised coefficient.
CBCL = Child Behaviour Checklist externalising sub-score at 18 months. Q-CHAT = Quantitative Checklist for Autism in Toddlers score at 18 months. Maternal EPDS = maternal Edinburgh Postnatal Depression Scale score at term-equivalent age. Multiple pregnancy = dummy variable of twin/triplet pregnancy. Parity = dummy variable, one/two/three+ previous child(ren). Gestation (weeks) = dummy variable: 34-36+6 weeks and ≥37 weeks gestation at birth. Corrected age at assessment (months) = age at behavioural assessment, corrected for gestational age. Cognition = infant Bayley III score at 18 months.

For peer review only

Supplementary Table 4: EPDS score predictors.

	IRR [95%CI]	p
**Time-lag (weeks)**	1.01 [0.97, 1.05]	.647
**Gestation:term**	0.91 [0.64, 1.31]	.627
**IMD rank**	1.00 [1.00, 1.00]	.103
**Multiple pregnancy**	0.66 [0.46, 0.96]	.031 *
**Parity**		
**1**	0.79 [0.66, 0.95]	.011 *
**2**	0.87 [0.60, 1.28]	.491
**3+**	0.84 [0.54, 1.31]	.445
**Birthweight (kg)**	0.98 [0.83, 1.17]	.847
**Sex:female**	1.13 [0.98, 1.31]	.098

$p<0.05$ *; $p<0.01$ **; $p<0.001$ ***

Pseudo $R^2 = 0.0228$

IRR = incidence rate ratio

Outcome variable = maternal Edinburgh Postnatal Depression Scale (EPDS) score at term-equivalent age. Time-lag (weeks) = time in weeks between birth and EPDS assessment. Gestation:term = dummy variable, term (≥37 weeks) vs preterm (<37 weeks) gestation at birth. Multiple pregnancy = dummy variable of twin/triplet pregnancy. Parity = dummy variable, one/two/three+ previous child(ren).

For peer review only

Supplementary Table 5: EPDS score predictors including interaction ‘term x time-lag’.

	IRR [95%CI]	p
**Time-lag (weeks)**	1.00 [0.97, 1.04]	.823
**Gestation: term**	0.88 [0.58, 1.34]	.553
**IMD rank**	1.00 [1.00, 1.00]	.104
**Multiple pregnancy**	0.66 [0.46, 0.96]	.029 *
**Parity**		
**1**	0.79 [0.66, 0.95]	.010 *
**2**	0.87 [0.60, 1.28]	.480
**3+**	0.85 [0.55, 1.31]	.458
**Birthweight (kg)**	0.97 [0.83, 1.15]	.756
**Sex:female**	1.13 [0.98, 1.30]	.100
**Term x time-lag (weeks)**	1.01 [0.94, 1.10]	.735

$p<0.05$ *; $p<0.01$ **; $p<0.001$ ***

Pseudo $R^2 = 0.0230$

IRR = incidence rate ratio

Outcome variable = maternal Edinburgh Postnatal Depression Scale (EPDS) score at term-equivalent age. Time-lag (weeks) = time in weeks between birth and EPDS assessment. Gestation: term = dummy variable, term (≥37 weeks) vs preterm (<37 weeks) gestation at birth. Multiple pregnancy = dummy variable of twin/triplet pregnancy. Parity = dummy variable, one/two/three+ previous child(ren). Term x time-lag (weeks): interaction term between term gestation at birth and time-lag between birth and maternal EPDS assessment.

For peer review only

Supplementary Figure 1: Histogram showing the distribution of maternal EPDS scores at term-equivalent age.

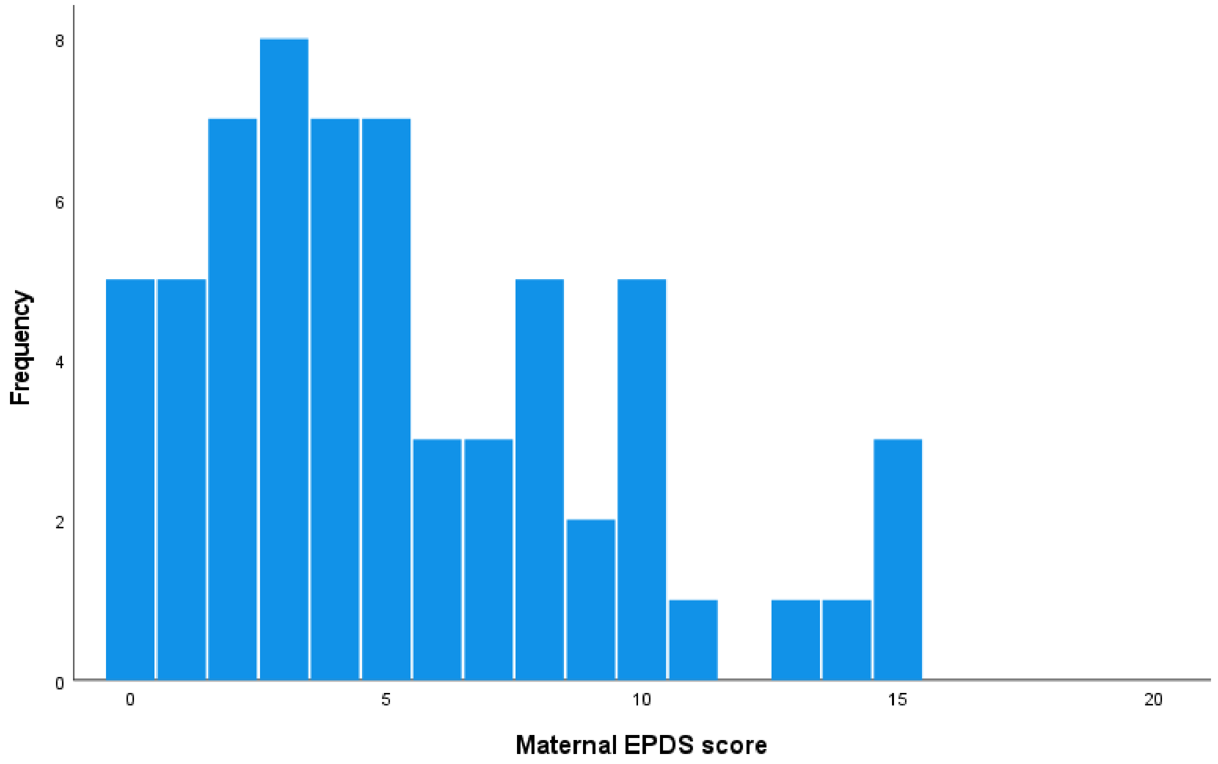

For peer review only - http://bmjopen.bmj.com/site/about/guidelines.xhtml

Supplemental reference list

1. Cohen J. *Statistical Power Analysis for the Behavioral Sciences*. 2nd ed. L. Erlbaum Associates; 1988.

For peer review only

STROBE Statement—Checklist of items that should be included in reports of ***cohort studies***

	Item No	Recommendation	Page No
**Title and abstract**	1	(*a*) Indicate the study's design with a commonly used term in the title or the abstract<br>(*b*) Provide in the abstract an informative and balanced summary of what was done and what was found	1, 2
**Introduction**			
Background/rationale	2	Explain the scientific background and rationale for the investigation being reported	5, 6
Objectives	3	State specific objectives, including any prespecified hypotheses	6
**Methods**			
Study design	4	Present key elements of study design early in the paper	6, 7
Setting	5	Describe the setting, locations, and relevant dates, including periods of recruitment, exposure, follow-up, and data collection	7
Participants	6	(*a*) Give the eligibility criteria, and the sources and methods of selection of participants. Describe methods of follow-up<br>(*b*) For matched studies, give matching criteria and number of exposed and unexposed	7
Variables	7	Clearly define all outcomes, exposures, predictors, potential confounders, and effect modifiers. Give diagnostic criteria, if applicable	7, 8
Data sources/ measurement	8*	For each variable of interest, give sources of data and details of methods of assessment (measurement). Describe comparability of assessment methods if there is more than one group	7, 8
Bias	9	Describe any efforts to address potential sources of bias	9
Study size	10	Explain how the study size was arrived at	7
Quantitative variables	11	Explain how quantitative variables were handled in the analyses. If applicable, describe which groupings were chosen and why	9, 10
Statistical methods	12	(*a*) Describe all statistical methods, including those used to control for confounding<br>(*b*) Describe any methods used to examine subgroups and interactions<br>(*c*) Explain how missing data were addressed<br>(*d*) If applicable, explain how loss to follow-up was addressed<br>(*e*) Describe any sensitivity analyses	9, 10, 11
**Results**			
Participants	13*	(a) Report numbers of individuals at each stage of study—eg numbers potentially eligible, examined for eligibility, confirmed eligible, included in the study, completing follow-up, and analysed<br>(b) Give reasons for non-participation at each stage<br>(c) Consider use of a flow diagram	7, 11
Descriptive data	14*	(a) Give characteristics of study participants (eg demographic, clinical, social) and information on exposures and potential confounders<br>(b) Indicate number of participants with missing data for each variable of interest<br>(c) Summarise follow-up time (eg, average and total amount)	9, 11
Outcome data	15*	Report numbers of outcome events or summary measures over time	11

For peer review only - http://bmjopen.bmj.com/site/about/guidelines.xhtml

Main results	16	(*a*) Give unadjusted estimates and, if applicable, confounder-adjusted estimates and their precision (eg, 95% confidence interval). Make clear which confounders were adjusted for and why they were included<br>(*b*) Report category boundaries when continuous variables were categorized<br>(*c*) If relevant, consider translating estimates of relative risk into absolute risk for a meaningful time period	11, 12
Other analyses	17	Report other analyses done—eg analyses of subgroups and interactions, and sensitivity analyses	11, 12
**Discussion**			
Key results	18	Summarise key results with reference to study objectives	12
Limitations	19	Discuss limitations of the study, taking into account sources of potential bias or imprecision. Discuss both direction and magnitude of any potential bias	15, 16, 17
Interpretation	20	Give a cautious overall interpretation of results considering objectives, limitations, multiplicity of analyses, results from similar studies, and other relevant evidence	13, 14, 17
Generalisability	21	Discuss the generalisability (external validity) of the study results	13, 14
**Other information**			
Funding	22	Give the source of funding and the role of the funders for the present study and, if applicable, for the original study on which the present article is based	3

*Give information separately for exposed and unexposed groups.

Note: An Explanation and Elaboration article discusses each checklist item and gives methodological background and published examples of transparent reporting. The STROBE checklist is best used in conjunction with this article (freely available on the Web sites of PLoS Medicine at http://www.plosmedicine.org/, Annals of Internal Medicine at http://www.annals.org/, and Epidemiology at http://www.epidem.com/). Information on the STROBE Initiative is available at http://www.strobe-statement.org.

For peer review only

BMJ Open

Postnatal maternal depressive symptoms and behavioural outcomes in term- and preterm-born toddlers: a longitudinal UK community cohort study.

Journal:	*BMJ Open*
Manuscript ID	bmjopen-2021-058540.R3
Article Type:	Original research
Date Submitted by the Author:	05-Aug-2022
Complete List of Authors:	Kleine, Ira; King's College London, Centre for the Developing Brain, School of Bioengineering and Imaging Sciences, Faculty of Life Sciences & Medicine<br>Vamvakas, George; King's College London, Department of Biostatistics and Health Informatics, Institute of Psychiatry, Psychology and Neuroscience<br>Lautarescu, Alexandra; King's College London, Centre for the Developing Brain, School of Bioengineering and Imaging Sciences, Faculty of Life Sciences & Medicine; King's College London, Forensic and Neurodevelopmental Sciences, Institute of Psychiatry, Psychology and Neuroscience<br>Falconer, Shona; King's College London, Centre for the Developing Brain, School of Bioengineering and Imaging Sciences, Faculty of Life Sciences & Medicine<br>Chew, Andrew; King's College London, Centre for the Developing Brain, School of Bioengineering and Imaging Sciences, Faculty of Life Sciences & Medicine<br>Counsell, Serena ; King's College London, Centre for the Developing Brain, School of Bioengineering and Imaging Sciences, Faculty of Life Sciences & Medicine<br>Pickles, Andrew; King's College London, Department of Biostatistics and Health Informatics, Institute of Psychiatry, Psychology and Neuroscience<br>Edwards, David; King's College London, Centre for the Developing Brain, School of Bioengineering and Imaging Sciences, Faculty of Life Sciences & Medicine<br>Nosarti, Chiara; King's College London, Centre for the Developing Brain, School of Bioengineering and Imaging Sciences, Faculty of Life Sciences & Medicine; King's College London, Department of Child and Adolescent Psychiatry, Institute of Psychiatry, Psychology and Neuroscience
**Primary Subject Heading**:	Mental health
Secondary Subject Heading:	Paediatrics
Keywords:	MENTAL HEALTH, Child & adolescent psychiatry < PSYCHIATRY, Developmental neurology & neurodisability < PAEDIATRICS, Depression & mood disorders < PSYCHIATRY, NEONATOLOGY

I, the Submitting Author has the right to grant and does grant on behalf of all authors of the Work (as defined in the below author licence), an exclusive licence and/or a non-exclusive licence for contributions from authors who are: i) UK Crown employees; ii) where BMJ has agreed a CC-BY licence shall apply, and/or iii) in accordance with the terms applicable for US Federal Government officers or employees acting as part of their official duties; on a worldwide, perpetual, irrevocable, royalty-free basis to BMJ Publishing Group Ltd ("BMJ") its licensees and where the relevant Journal is co-owned by BMJ to the co-owners of the Journal, to publish the Work in this journal and any other BMJ products and to exploit all rights, as set out in our licence.

The Submitting Author accepts and understands that any supply made under these terms is made by BMJ to the Submitting Author unless you are acting as an employee on behalf of your employer or a postgraduate student of an affiliated institution which is paying any applicable article publishing charge ("APC") for Open Access articles. Where the Submitting Author wishes to make the Work available on an Open Access basis (and intends to pay the relevant APC), the terms of reuse of such Open Access shall be governed by a Creative Commons licence – details of these licences and which Creative Commons licence will apply to this Work are set out in our licence referred to above.

Other than as permitted in any relevant BMJ Author's Self Archiving Policies, I confirm this Work has not been accepted for publication elsewhere, is not being considered for publication elsewhere and does not duplicate material already published. I confirm all authors consent to publication of this Work and authorise the granting of this licence.

For peer review only

Postnatal maternal depressive symptoms and behavioural outcomes in term- and preterm-born toddlers: a longitudinal UK community cohort study.

Kleine, I[a] (MBBS); Vamvakas, G[b] (MSc); Lautarescu A[a,c] (MPhil); Falconer, S[a] (PhD); Chew A[a] (MD); Counsell SJ[a] (PhD); Pickles, A[b] (FMedSci); Edwards, AD[a] (FMedSci); Nosarti, C[a,d] (PhD).

[a] Centre for the Developing Brain, School of Bioengineering and Imaging Sciences, Faculty of Life Sciences & Medicine, King's College London, St Thomas' Hospital, London SE1 7EH, UK.

[b] Department of Biostatistics and Health Informatics, Institute of Psychiatry, Psychology and Neuroscience, King's College London, London SE5 8AF, UK.

[c] Forensic and Neurodevelopmental Sciences, Institute of Psychiatry, Psychology and Neuroscience, King's College London, London SE5 8AF, UK.

[d] Department of Child and Adolescent Psychiatry, Institute of Psychiatry, Psychology and Neuroscience, King's College London, London SE5 8AF, UK.

Post-publication corresponding author's contact information

Prof Chiara Nosarti

Centre for the Developing Brain, School of Bioengineering and Imaging Sciences

King's College London

St Thomas' Hospital

London

SE1 7EH

UK

chiara.nosarti@kcl.ac.uk

+44 (0)207 848 0133

For peer review only - http://bmjopen.bmj.com/site/about/guidelines.xhtml

Abstract

Objectives: To examine the association between maternal depressive symptoms in the immediate postnatal period and offspring's behavioural outcomes in a large cohort of term- and preterm-born toddlers.

Design and Participants: Data were drawn from the Developing Human Connectome Project. Maternal postnatal depressive symptoms were assessed at term-equivalent age, and children's outcomes were evaluated at a median corrected age of 18.4 months (range 17.3 – 24.3).

Exposure and outcomes: Preterm birth was defined as <37 weeks completed gestation. Maternal depressive symptoms were assessed with the Edinburgh Postnatal Depression Scale (EPDS). Toddlers' outcome measures were parent-rated Child Behaviour Checklist $1^{1/2}$-5 Total (CBCL) and Quantitative Checklist for Autism in Toddlers (Q-CHAT) scores. Toddlers' cognition was assessed with the Bayley Scales of Infant and Toddler Development – Third Edition (Bayley-III).

Results: Higher maternal EPDS scores were associated with toddlers' higher CBCL (B=0.93, 95% CI 0.43-1.44, p<0.001, f^2=0.05) and Q-CHAT scores (B=0.27, 95% CI 0.03-0.52, p=.031, f^2=0.01). Maternal EPDS, toddlers' CBCL and Q-CHAT scores did not differ between preterm (n=97; 19.1% of the total sample) and term participants. Maternal EPDS score did not disproportionately affect preterm children with respect to CBCL or Q-CHAT scores.

Conclusions: Our findings indicate that children whose mothers reported increased depressive symptoms in the early postnatal period, including subclinical symptoms, exhibit more parent-reported behavioural problems in toddlerhood. These associations were independent of gestational age. Further research is needed to confirm the clinical significance of these findings.

For peer review only - http://bmjopen.bmj.com/site/about/guidelines.xhtml

Strengths and limitations of this study

- Prospective study with a large sample, using multiple imputation to reduce non-response bias.
- Maternal depressive symptoms assessed as a continuous variable, providing more nuanced information about the significance of subclinical symptoms.
- Maternal depressive symptoms assessed earlier than in previous studies, enabling recognition of early screening opportunities for families.
- Potential common method variance bias through parent-completed child behavioural assessments.
- Unknown paternal and parental factors, such as comorbid psychiatric conditions, that may confound our findings.

Keywords

Postpartum Depression; Child Development; Autism; Mental Health; Child Behavior; Mood Disorder; Preterm Birth.

For peer review only

Introduction

Postnatal depression affects approximately 12% of mothers worldwide.[1] In contrast to 'baby blues', which is a state of emotional lability that affects between 13.7%-76.0% of women in the first few days after birth and typically resolves spontaneously within two weeks,[2] postnatal depression is more severe and starts in the first few months post-partum[1]. Stressful life events have been linked to a heightened risk of developing postnatal depression;[3] for example, mothers of preterm infants have a significantly higher risk of postpartum depression compared to mothers of term infants,[4] likely due to heightened stress associated with perinatal complications.[5]

Women with postnatal depression tend to be less responsive to their baby's needs and to display less affection.[6] Therefore, in the short-term postpartum depression may affect mother-infant interactions[7] and in the long-term it may lead to alterations in brain development,[8] emotional difficulties,[9] less secure attachment, cognitive and behavioural problems in childhood, and a possible increased risk of autism spectrum disorder (ASD).[10,11] Large cohort studies, such as the Avon Longitudinal Study of Parents and Children (ALSPAC), have shown that these associations are even evident when maternal depression is measured on a continuum of symptoms rather than a dichotomous diagnosis,[12–14] supporting the notion that elevated sub-diagnostic psychiatric symptoms can also negatively impact on children's development.[15]

Studies investigating the underlying causes that may link maternal postnatal depression to child outcomes have implicated several biological and environmental variables. For instance, genetic and epigenetic factors have been shown to both mediate and mitigate the intergenerational transmission of psychiatric disorders,[16] while lower quality parenting, interparental conflict, and socioeconomic deprivation have been shown to exacerbate children's developmental risk of emotional and behavioural problems.[11] In addition, being born preterm (i.e. <37 weeks' gestation, as per the World Health Organization definition [17]) has been associated with alterations in early brain development,[18] as well as neurological, behavioural and cognitive problems in childhood and beyond.[19,20] Therefore, it is complex to disentangle the possible effects of postnatal maternal mental health and those of perinatal clinical factors on specific outcomes in preterm children, as these may involve both maternal psychosocial and biological variables, as well as child preterm-related neurodevelopmental morbidity.

For peer review only - http://bmjopen.bmj.com/site/about/guidelines.xhtml

Furthermore, a question that remains unanswered is whether preterm birth accentuates the association between maternal postnatal depression and child outcome. Two theoretical frameworks exist that hypothesise certain infants may be influenced differently by external stimuli: the diathesis stress model proposes that certain vulnerability factors make affected infants more prone to suboptimal environmental influences with subsequent poorer outcomes,[21,22] whereas the differential susceptibility model frames such factors as plasticity-mediating, thus leading to poorer outcomes in negative environments, as well as better outcomes in supportive environments.[22,23] Previous studies investigating differential susceptibility have shown mixed findings studying a range of environmental and clinical exposures,[24,25] with child outcomes including attachment, internalising and externalising behaviour, and academic competence.[25] Both low birthweight in term infants (small for gestational age, SGA) [26] and preterm birth (PTB) [23,24,27] have been explored as distinct potential susceptibility factors. This distinction is based on the different pathophysiological processes underlying the respective conditions of SGA and PTB, both, or a combination, of which can cause low birthweight.[28] For example, SGA is a marker of intra-uterine growth restriction related to placental dysfunction,[29] whereas PTB can be caused by a multitude of factors, including infection and inflammation.[30]

Given that mothers of preterm children experience elevated levels of distress,[31] are at high risk of developing postnatal depression,[32] and that preterm children themselves are vulnerable to psychiatric sequelae,[33] in addition to investigating the association between very early maternal postnatal depressive symptoms and child behavioural and emotional outcomes, we further aimed to investigate the interaction between preterm birth and maternal depressive symptoms on child outcomes. Previous work focusing on the differential susceptibility of preterm born children to various environmental stimuli, as described above, had not yet studied maternal depressive symptoms as a proposed exposure. We specifically aimed to investigate the continuum of maternal depressive symptoms rather than solely focussing on clinically significant maternal depression, so as to provide more nuanced information about the importance of subclinical depressive symptoms on child outcomes. We hypothesised that early postnatal maternal depressive symptoms would be more elevated in mothers of preterm compared to term infants and that these would impact preterm children's behavioural and emotional outcomes to a greater degree than their term counterparts.

For peer review only - http://bmjopen.bmj.com/site/about/guidelines.xhtml

Methods

Sample

Participants were enrolled in the Developing Human Connectome Project (DHCP, http://www.developingconnectome.org/), a neuroimaging-focused project, with eligibility criteria including pregnant women (aged ≥16 years) with a gestational age of 20–42 weeks, and newborn infants aged 24–44 weeks; infants enrolled in the DHCP had magnetic resonance imaging (MRI) at term-equivalent age. Exclusion criteria for the DHCP included: contraindications to MRI, babies being too unwell to tolerate a scan, and language difficulties preventing informed consent.[34] Toddlers were invited to the Centre for the Developing Brain, St Thomas' Hospital, London, for neurodevelopmental assessment at 18 months post-expected delivery date; appointments were made according to family availability as close as possible to this time-point. Inclusion criteria for our follow-up study were: mother and baby attendance for MRI at term-equivalent age; completed toddler neurodevelopmental assessment. These inclusion criteria were met by 509 toddlers by the date of closure for this analysis (26/02/2020). Of the 509 toddlers, 51 were one of a twin pregnancy, and three were one of a triplet pregnancy; the sample contained 22 sibling pairs and one set of triplets. This study was approved by the UK National Research Ethics Authority (14/LO/1169) and conducted in accordance with the World Medical Association's Code of Ethics (Declaration of Helsinki). Written informed consent was given by children's carer(s) at recruitment into the study.

Maternal variables

Maternal age, parity, Body Mass Index (BMI), ethnicity and postcode were collected at enrolment into the DHCP study. Our sample was ethnically representative of the surrounding geographical area. Parity was coded as 0, 1, 2, or ≥3 previous children. Index of Multiple Deprivation (IMD) rank was computed from the current maternal postcode using the 2019 IMD classification; it combines locality-specific information about income, employment, education, health, crime, housing and living environment, thus providing a proxy for family socioeconomic status.[35] Lower IMD rank corresponds to greater social deprivation. Our sample was generally less deprived than the surrounding geographical areas, as well as the UK as a whole, reflecting trends observed in other UK longitudinal studies.[36]

Maternal depressive symptoms were measured using the Edinburgh Postnatal Depression Scale (EPDS)[37] on the day of infant's MRI at term-equivalent age. Mothers of infants born at

For peer review only - http://bmjopen.bmj.com/site/about/guidelines.xhtml

term were tested in the first few weeks postnatally, whereas mothers of preterm-born infants were tested once they reached term-corrected age. The EPDS is a 10-item screening questionnaire completed by mothers, with higher scores reflecting a higher likelihood of depressive disorders. A score of 13 can be used as a cut-off indicating high-level symptoms, although a cut-off of 11 maximises the sensitivity and specificity of the screening tool for depression.[38] Mothers completed the EPDS independently in a private room in our Centre, with no interaction with the researcher. Participants were informed that the results would be discussed with them, and consented to information being shared with their General Practitioner in the case of high scores. The EPDS questionnaire was scored by a member of the DHCP team.[34]

Child variables

Infant *clinical characteristics* were gathered from clinical notes where available, or from maternal report, and included: sex, gestational age at birth, birth weight, and pregnancy size (singleton/twin/triplet).

Behavioural outcomes were assessed using the Child Behaviour Checklist/$1^{1/2}$-5 (CBCL), a parent-completed 100-item questionnaire, in which the parent rates the child's behaviour over the preceding two months using a 3-point Likert scale ("not true", "somewhat or sometimes true", and "very true or often true"). Responses are categorised into syndrome profiles, and these are subsequently grouped into internalising (emotional reactivity, anxiety/depression, somatic complaints, and withdrawal), externalising (attention problems, aggressive behaviour) and total (internalising, externalising, sleep and other) problem scales. Higher scores indicate increased emotional and behavioural problems. Total scores are classified into a normal range (<83rd centile, T <60), borderline range (83rd-90th centile, T 60-63), and clinical range (>90th centile, T ≥64).[39] The CBCL is known to have high reliability, validity and cross-informant agreement for measuring children's emotional and behavioural problems.[39]

We used the Quantitative Checklist for Autism in Toddlers (Q-CHAT) as an additional behavioural screening tool to broaden the exploration of mental health outcomes in toddlers. The Q-CHAT is a parent-completed 25-item questionnaire, in which the child's behaviour is scored on a 5-point (0-4) frequency scale. Higher total scores correspond to a higher frequency of behaviours also observed in autism spectrum conditions. The Q-CHAT shows

For peer review only

For peer review only - http://bmjopen.bmj.com/site/about/guidelines.xhtml

good test-retest reliability, face validity and specificity, yet poor positive predictive value for autism,[40,41] highlighting that higher Q-CHAT scores may reflect developmental immaturity rather than autism.[41]

Cognitive assessment was performed using the Bayley Scales of Infant and Toddler Development – Third Edition (Bayley-III). The Bayley-III provides scores for a child's overall cognitive, language and motor development. The cognitive standardised composite score was used in this study; scores between 70-84 indicate mild cognitive impairment, scores between 55-69 indicate moderate impairment, and scores lower than 55 indicate severe impairment[42]. Reliability and validity of the Bayley-III have been shown to be robust,[43] although some studies report its underestimation of developmental problems.[44]

Assessments were carried out by staff experienced in the neurocognitive assessments of toddlers.

Analysis

Descriptive statistics and one-way ANOVA tests were performed in IBM SPSS Statistics for Windows v.25. All other analyses were carried out in Stata v.16.

Multiple imputation (MI) was carried out to account for missing data in CBCL (11/509, 2.24%), Q-CHAT (9/509, 1.8%), maternal EPDS (73/509, 14.3%), maternal BMI (27/509, 5.3%) and IMD rank (3/509, 0.6%). Variables were imputed simultaneously using the 'mi impute chained' procedure that performs imputation by chained equations. The imputation models had the same structural form as the analysis models, and included all variables that appear in the corresponding analysis models (maternal EPDS, maternal BMI, multiple pregnancy, parity, IMD rank, gestational age at birth, birthweight, sex, corrected age at assessment, and Bayley III Cognitive Composite score). In the imputation models we also included variables that were associated with the incomplete variables at the 20% level. As such, maternal age was included in the imputation model because it was found to be a significant predictor of the total CBCL raw score (p=0.001), the Q-CHAT score (p=0.021) and EPDS score (p=0.122) when it was included as an independent variable in regression models.

For peer review only - http://bmjopen.bmj.com/site/about/guidelines.xhtml

Maternal EPDS and CBCL were imputed using Poisson regression; Q-CHAT, maternal BMI, and the IMD rank were imputed using linear regression. 40 MI datasets were created. To assess the stability of our MI parameters, we extracted the Monte Carlo error of each parameter estimate and examined whether the error for the coefficient was less than 10% of the parameter's standard error estimate. MI estimates were used for the primary analyses and compared to the estimates from complete-case (CC, individuals who had no missing data pre-imputation) analyses. Conditional normality was inspected in the complete-case analyses using QQ plots of the residuals of the models. Sensitivity analyses with and without extreme values were conducted. Initially, we fit the model using all available data, constructed the residuals and examined the QQ plot. Extreme values were then removed, models re-fitted without these values, and new QQ plots of residuals constructed again to check for any new extreme values. This process was repeated as many times as needed to remove all extreme values. During this process, the resulting estimates from the models were being examined as to whether they had substantially changed. We found that the removal of extreme values did not make any difference to the estimated parameters, and hence present the results from the full sample.

The analysis models were multiple linear regressions fitted using the 'mi estimate' procedure, which estimates effects after application of Rubin's rules.[45] To account for the small amount of clustering in our data (twin/triplet siblings), the models' standard errors were obtained using Stata's robust cluster estimator 'vce(cluster *idvar*)'. For continuous variables, Cohen's f-squared effect sizes were calculated using $f^2 = (R_{AB}^2 - R_A^2)/(1 - R_{AB}^2)$, where R_{AB}^2 is the R-squared value from a regression model that includes the variable of interest as well as all the covariates used in the model, and R_A^2 is the R-squared value from the regression model that includes only the covariates.[46,47] For binary variables, Cohen's f-squared effect sizes were produced after estimating first the Cohen's d using the formula: $f^2 = \frac{d}{2k}$, where k is the number of groups. As a measure of dispersion, Cohen's d used the average root mean-square error over the MI datasets. Adjusted R-squared values after MI were extracted after estimating the model with the user-written 'mibeta' command with the 'fisherz' option,[48] which calculates R-squared measures for linear regression with MI data. The significance of the joint effect of the categorical variable parity was assessed using `mi test' which performs Wald tests of composite linear hypotheses.

For peer review only

Primary outcome measures were children's total CBCL raw score and Q-CHAT score. S*econdary outcome measures* were CBCL internalising and externalising scores. The effect of maternal EPDS score was adjusted for IMD rank, maternal age, maternal BMI, maternal parity, pregnancy size, and the following child's variables: continuous gestational age, birth weight, Bayley-III cognitive composite score, and corrected age at assessment. The interaction between preterm birth and maternal depressive symptoms was explored using a complete case analysis in both CBCL and Q-CHAT models, using a dichotomised measure of gestational age. EPDS, CBCL and Q-CHAT scores were compared between term (≥37 weeks gestation) and preterm infants (<37 weeks gestation) using the complete case dataset. Our regressions were thus run twice: with and without the interaction term.

As all mothers had their EPDS score measured near term (or term-corrected in the case of mothers of preterm infants), we further investigated the association between time elapsing between baby's birth and mother's EPDS assessment and EPDS score, in order to avoid erroneously identifying 'baby blues' in mothers of term-born infants versus postnatal depression in mothers of preterm infants. This post-hoc analysis was performed using Poisson regression.

Patient and Public Involvement

The current study was developed in consultation with the Weston Programme for Family Centered Research, which involves parents to define what research is valuable to them, and to allow them to lead it with support from the scientists in the Centre for the Developing Brain.

Results

Descriptive statistics

Our sample of 509 toddlers were followed up at a median corrected age of 18.4 months (range 17.3 – 24.3 months). 51 (10.0%) of these were twins, and 3 (0.59%) were triplets. Of the 509, 21 (4.13%) mothers scored above a clinical cut-off (≥13) on the EPDS;(26) the distribution of maternal EPDS scores is shown graphically in Supplementary Figure 1. Demographic data are shown in Table 1. Complete data were available for 400 (78.6%) participants. Missing data were imputed and thus all 509 subjects were included in the primary and secondary analyses. One participant was excluded from the cognition analysis after examining the quintiles of the residuals against the theoretical quintiles of a normal distribution. The mean CBCL T score was 46.9 (SD 9.5) (Table 1); using CBCL-specified

For peer review only - http://bmjopen.bmj.com/site/about/guidelines.xhtml

cut-offs,[39] 449 (90.2%) of participants had a CBCL score in the normal range, 30 (6.0%) were borderline, and 19 (3.8%) scored in the clinical range. The mean Q-CHAT score was 30.5 (SD 9.3) (Table 1). The mean Bayley III Cognitive Composite score in our sample was 100 (SD 11.4) (Table 1), which corresponds to the standardised test mean;[42] 480 (94.3%) of participants had a normal cognitive score, 24 (4.7%) had mild impairment, 5 (1%) had moderate impairment, and nil had severe impairment. This distribution is not dissimilar from that of the normative sample.[42]

Association between maternal EPDS score and toddler CBCL and Q-CHAT scores

Predictors of children's CBCL and Q-CHAT scores after multiple imputation are shown in Table 2. Higher maternal EPDS score was associated with children's higher CBCL Total score (B=0.93, 95% CI 0.43-1.44, $p<0.001$, $f^2=0.05$) and Q-CHAT score (B=0.27, 95% CI 0.03-0.52, p=.031, $f^2=0.01$) (Table 2). These associations are presented graphically in Figure 1 and Figure 2, respectively. Boys had higher CBCL and Q-CHAT scores than girls. Higher Q-CHAT scores were associated with lower IMD rank (i.e., greater socio-economic deprivation) and lower Bayley-III cognitive composite scores. Parity was not a significant predictor of outcome in any of the models (Table 2).

Maternal EPDS score did not disproportionately affect preterm children with respect to CBCL or Q-CHAT scores (Table 3).

Association between maternal EPDS score and toddler CBCL internalising and externalising scores

Higher maternal EPDS score was associated with both internalising (B=0.22, 95% CI 0.08-0.36, $p<0.01$, $f^2=0.03$) and externalising (B=0.40, 95% CI 0.20-0.61, $p<0.001$, $f^2=0.05$) symptoms in children (Supplementary Table 1 and 2, respectively). Comparison of the imputed model analyses to the complete-case analyses showed that results were consistent for the CBCL model (Supplementary Table 3). Comparison for the Q-CHAT model showed that maternal EPDS was a significant predictor in the imputed model, but not in the complete-case analysis (Supplementary Table 3).

Effect of time-lag between baby's birth and mother's EPDS assessment and EPDS score

Mothers who gave birth prematurely (<37 weeks gestation) had their EPDS score assessed on average 7.7 weeks later post-delivery than mothers who gave birth at term (preterm

For peer review only - http://bmjopen.bmj.com/site/about/guidelines.xhtml

participants M=8.9 (SD 4.8), term participants M=1.2 (SD 1.3); t(99.4)=15.5, p<.001). The time-lag between birth and EPDS assessment did not predict maternal EPDS score, and there was no evidence of a significant interaction between gestation and birth-to-assessment time-lag (Supplementary Table 4 and 5, respectively).

Discussion

Principal findings

Our results showed that more maternal self-reported depressive symptoms shortly after birth were associated with greater parent-reported toddlers' behavioural problems. Given that fewer than 5% of the mothers in our cohort had EPDS scores above a clinical threshold,[37] our findings indicate that even subclinical depressive symptoms – i.e. not only diagnostic postnatal depression – adversely impact children's behavioural outcomes. In addition, our cohort was typically developing with few CBCL scores reaching a concerning threshold; our results could be interpreted within the conceptual framework of mental illness lying on a continuum with typical behavioural traits.[49] Our findings further showed that preterm birth did not influence the association between self-reported maternal depressive symptoms and parent-reported infants' behavioural outcomes in toddlerhood. This indicates that in this context preterm birth may not be regarded as a vulnerability or plasticity factor. Interestingly, mothers of preterm infants did not report more depressive symptoms compared to mothers of term infants in this study.

Comparison to prior literature

Our results with respect to internalising and externalising symptoms are in line with previous studies, including large population cohort studies, that showed an association between postnatal maternal depression and young children's emotional and behavioural problems.[11] Another previous study in 18-month old toddlers found that maternal depression was associated with internalising and dysregulated behaviour, but not externalising symptoms.[50] This difference between our and Conroy et al.'s findings may have arisen from their exclusion of infants born <36 weeks and their use of a clinical diagnosis of depression for mothers, rather than the continuous self-reported approach we employed. Interestingly, our finding that even subclinical depressive symptoms may adversely impact parent-reported child behavioural outcomes is in line with recent data showing that low- as well as high-level depressive symptoms are associated with internalising and externalising symptoms in children aged 3 years.[51]

For peer review only - http://bmjopen.bmj.com/site/about/guidelines.xhtml

The results showing an association between maternal postnatal depressive symptoms and the Q-CHAT are less robust and need to be interpreted with caution. Firstly, these results must be viewed in the context of the Q-CHAT having a low positive predictive value for autism, with the measure perhaps being more reflective of developmental immaturity. [41] Although some prior studies have shown an association between antenatal maternal depression and offspring's ASD,[10,52] and postnatal depression has been suggested as a potential focus of cross-domain studies of ASD,[53] there is no clear aetiological role of maternal postnatal depression in the development of ASD *per se*. Also, given that mothers with ASD are more likely to suffer from perinatal depression than mothers without ASD,[54] and ASD is highly heritable,[55] maternal depression may actually be a confounding rather than causative factor in our observed results. Overall, therefore, our findings with respect to the Q-CHAT do not provide support for a role of maternal depression in the aetiology of autism traits, but rather suggest that maternal depression can influence toddler behaviour.

The finding that preterm infants were not disproportionately affected by maternal depressive symptoms supports Hadfield et al.'s findings that maternal distress at 9 months did not differentially impact very preterm (<34 weeks) or late preterm (34-36^{+6} weeks) infants with respect to socioemotional outcomes, although paternal distress did have an impact on very preterm infants' outcomes.[24] However, our results differ from Gueron-Sela et al.'s finding that very preterm (28-33 weeks) 12 month old infants' social outcomes were more influenced by maternal emotional distress at 6 months than term infants' outcomes.[23] The inconsistent findings may be due to methodological differences: for instance, our infant assessment being conducted at 18 months corrected age when social competency is more developed, our assessment of maternal depressive symptoms being in the very early postnatal period, or our use of the CBCL and Q-CHAT tools as markers of toddler behaviour. Importantly, the lack of support for a diathesis-stress or differential susceptibility model of maternal mental state on preterm infants in our study must be viewed in the context of our results also showing no difference in CBCL and Q-CHAT scores between term and preterm infants. This is in contrast to the existing literature that preterm infants are more likely than term infants to develop behavioural problems, such as ADHD, in childhood and adolescence.[20,33] It is possible that the phenotypes of neurodevelopmental and neuropsychiatric disorders assessed with the chosen behavioural measures may not be sufficiently expressed at 18 months

For peer review only - http://bmjopen.bmj.com/site/about/guidelines.xhtml

corrected age.[56] In addition, as briefly discussed above, much of the existing literature emphasises the risk of extreme (<28 weeks) or very preterm (28-33 weeks) birth on later behavioural outcomes,[20,33] whereas only 3.5% and 5.5% of our participants fell within the extreme and very preterm group, respectively, and we thus may not have the power to show any subtle effects.

Strengths & limitations of the study

The strengths of this study lie primarily in its large sample and prospective data collection. Moreover, the use of multiple imputation methodology has facilitated retention of a complete dataset, thus minimising non-response bias and increasing parameter precision. A strength in comparison to prior population cohort studies is that we assessed very early maternal depressive symptoms, and our sample is perhaps more representative of today's society – with increasing maternal age – than large cohort studies conducted in the 1990s-2000s. Given the complex interplay of biological and environmental factors in the aetiology of behavioural disorders, the inclusion of a substantive proportion of preterm infants in our cohort also offers an important insight into the role of preterm birth in behavioural outcomes; moreover, our results represent the full gestational spectrum, rather than discrete gestational categories. In addition, using maternal depressive symptoms as a continuous, rather than dichotomous, variable allows a more nuanced understanding of the role maternal postnatal depressive symptoms may play in influencing children's outcomes.

There are several limitations to this study that necessitate our findings to be considered with caution. Firstly, differences in birth-to-EPDS-assessment time-lags are a potential confounder, given the time-sensitive nature of early-onset temporary baby blues and later-onset pathological postnatal depression. Mothers of infants born at term were assessed early post-delivery, within the period one would anticipate baby blues to present, whereas mothers of preterm participants were on average assessed later, when postnatal depression predominates.[1,57] Although our post-hoc analyses showed that the time elapsed from birth to EPDS assessment was not associated with maternal EPDS score, providing reassurance that our assessments of mothers of term-born infants were not inflated by the common temporary symptoms of baby blues, it is possible that we did not capture the full extent of later-onset depressive symptoms in mothers of term-born infants. This may explain why maternal EPDS scores did not differ between preterm and term groups in our complete dataset analysis, contrary to the current literature,[31] as well as why our rate of postpartum depression, using an

For peer review only - http://bmjopen.bmj.com/site/about/guidelines.xhtml

EPDS cut-off of 13, was low (4.1%) compared to the previously documented UK community prevalence rate of 8.9% at eight weeks postpartum.[58] Our results must therefore be interpreted with some caution.

Secondly, although statistical techniques were used to impute missing data and mitigate this problem, 14.3% of maternal EPDS scores were missing. This rate of missingness may relate to some mothers being reluctant to complete a questionnaire at the time their child is having an MRI, or due to simultaneous childcare duties. Thirdly, a number of important confounders that are likely to affect children's behavioural outcomes were not assessed in this study, including genetic risk for psychiatric disorders,[59] parental psychiatric co-morbidities,[50] chronicity of postnatal depressive symptoms,[51] antenatal maternal depression, paternal depression and subsequent parent-infant attachment, and inter-parental conflict.[11] Thus, we are unable to conclude whether our observed associations between early postnatal maternal depressive symptoms and children's behavioural outcomes are moderated or mediated by other parental and/or psychiatric factors.

Fourthly, whilst our study included a reasonable proportion of preterm infants (97/509, 19%), our sample was not random, as preterm children were selectively recruited for the DHCP; indeed, preterm infants are over-represented in our sample when compared to the UK population incidence (7.3%),[60] which may limit the study's generalisability to the general population. This over-representation of preterm infants may explain why our mean maternal age is higher than the national mean age of 30.7,[61] given that increasing maternal age is associated with increased risk of adverse pregnancy outcomes.[62] Our observed large maternal age range in itself also poses a limitation on the generalisability of our findings to the general population, and further research would be necessary to identify a possible moderation effect of high maternal age on both EPDS scores and child behavioural outcomes. Furthermore, although a 19% prevalence of preterm birth is high for a community sample, the proportion of very and extreme preterm infants in our sample is small, and this may not have provided sufficient power to detect any differential susceptibility effect of preterm birth on outcomes.

Sixthly, the effect sizes of the association between maternal EPDS score and behavioural problems were small; this raises questions regarding the clinical significance of our findings and potentially explains some of the inconsistency between this and previous studies. Even within our analyses, the association between maternal depressive symptoms and Q-CHAT

For peer review only - http://bmjopen.bmj.com/site/about/guidelines.xhtml

scores was not observed in our complete case analysis, thus calling into question the validity of this result. It is also important to highlight again the poor positive predictive value of the Q-CHAT for autism;[41] higher Q-CHAT scores do not imply a diagnosis of ASD, and this distinction may also explain the contrast to previous studies.

Finally, it is well documented that maternal depression influences reporting of Q-CHAT [63] and CBCL scores.[64] Our study used maternal report of maternal depressive symptoms, and our outcome measures were parent-completed questionnaires; despite the CBCL showing good cross-informant agreement,[39] it is thus possible that reporting bias with common method variance could have skewed our results.

Implications of our findings

Of greatest importance to clinicians and policymakers is our finding that even *subclinical* self-reported maternal depressive symptoms are associated with parent-reported behavioural outcomes of offspring. This has significant implications for the risk-stratification of women and their babies in the postnatal period, during which contact with medical professionals is already established. Identifying high risk families and providing appropriate supportive measures at the early postnatal stage may help to prevent future psychiatric morbidity.

Future research

Further follow-up of large cohorts of preterm and term infants, to an age when behavioural phenotypes may become more pronounced, is needed to investigate whether the long-term developmental trajectories of term and ex-preterm infants are differentially susceptible to changes of postnatal maternal mental health. Future research should consider both maternal and paternal mental health, as well as socioeconomic and environmental factors on child outcomes. Such follow-up should use independent, objective assessments of child behavioural outcomes in order to avoid the common method variance inherent to parent-reported measures. Finally, it is crucial for future research to elucidate the interplay of biochemical and neurodevelopmental changes that may mediate and confound the translation of environmental exposures into distal behavioural phenotypes.

Conclusion

This prospective longitudinal cohort study found no evidence to support the concept of preterm birth as a vulnerability or plasticity factor with respect to the effect of maternal

For peer review only - http://bmjopen.bmj.com/site/about/guidelines.xhtml

depressive symptoms on behavioural development. However, we showed that early subclinical maternal postnatal depressive symptoms were associated with behavioural problems in children on parent-reported measures. This adds to the increasing body of literature indicating the role of subclinical and early postnatal depressive symptoms in the aetiology of childhood behavioural disorders. These findings are of great relevance to child and public health, and further research may strengthen its implications for developing strategies to facilitate effective screening and support for women and children, enabling all to reach their full potential.

Contributorship & acknowledgements

We thank all DHCP investigators for their contribution to the study. We thank Dr Oliver Gale-Grant MRes (Centre for the Developing Brain, King's College London; Department of Forensic & Neurodevelopmental Sciences, King's College London) for providing the IMD rank data. We are very grateful to the families who generously took part in this research.

Conceptualization: SC, ADE, CN; **Methodology:** IK, GV, SC, AP, ADE, CN; **Investigation:** SF, AC; **Data curation:** IK, AL; **Formal analysis:** IK, GV, AP, CN; **Writing – original draft preparation:** IK; **Writing – Review & Editing:** GV, AL, SF, AC, SC, AP, ADE, CN; **Visualisation:** IK, GV; **Funding acquisition:** SC, ADE; **Supervision:** AP, ADE, CN.

Funding statement

The DHCP project was funded by the European Research Council under the European Union Seventh Framework Programme (FR/2007-2013)/ERC Grant Agreement no. 319456. The authors acknowledge infrastructure support from the National Institute for Health Research Mental Health Biomedical Research Centre at South London, Maudsley NHS Foundation Trust, King's College London, the National Institute for Health Research Mental Health Biomedical Research Centre at Guys, and St Thomas' Hospitals NHS Foundation Trust. The study was also supported in part by the Engineering and Physical Sciences Research Council / Wellcome Trust Centre for Medical Engineering at King's College London (grant WT 203148/Z/16/Z) and the Medical Research Council (UK) (grants MR/K006355/1 and MR/L011530/1) and the MRC Centre for Neurodevelopmental Disorders at King's College London. AP receives a NIHR SI award (NF-SI-0617-10120). AL is supported by the UK Medical Research Council (MR/N013700) and King's College London member of the MRC

For peer review only - http://bmjopen.bmj.com/site/about/guidelines.xhtml

Doctoral Training Partnership in Biomedical Sciences. The views expressed are those of the authors and not necessarily those of the NHS, the NIHR, or the Department of Health and Social Care.

Conflict of interest / Competing interests

ADE received financial support from the EU-AIMS-Trials (European Research Council under the European Union Seventh Framework Programme) as co-Principal Investigator. ADE received consulting fees from Chiesi Farmaceutici (advice on neuroprotection in newborn infants) and Medtronix (unpaid participation in scientific advice committee). ADE has a patent on Xenon as an organ protectant (No P023708WO). ADE was Chair of the Data Monitoring and Ethics Committee for the Baby-Oscar Trial, and served on the Data Monitoring and Ethics Committee for the PAEN Trial.

There are no other relationships or activities that could appear to have influenced the submitted work.

Ethics approval

This study was approved by the UK National Research Ethics Authority (14/LO/1169) and conducted in accordance with the World Medical Association's Code of Ethics (Declaration of Helsinki).

Consent to participate

Written informed consent was given by children's carer(s) at recruitment into the study.

Consent for publication

Not applicable.

Data sharing

Research data are available upon reasonable request.

Code availability

Not applicable.

For peer review only - http://bmjopen.bmj.com/site/about/guidelines.xhtml

References

1. Woody CA, Ferrari AJ, Siskind DJ, Whiteford HA, Harris MG. A systematic review and meta-regression of the prevalence and incidence of perinatal depression. *J Affect Disord*. 2017;219:86-92. doi:10.1016/j.jad.2017.05.003

2. Rezaie-Keikhaie K, Arbabshastan ME, Rafiemanesh H, Amirshahi M, Ostadkelayeh SM, Arbabisarjou A. Systematic Review and Meta-Analysis of the Prevalence of the Maternity Blues in the Postpartum Period. *J Obstet Gynecol Neonatal Nurs*. 2020;49(2):127-136. doi:10.1016/j.jogn.2020.01.001

3. Beck CT. Predictors of postpartum depression: an update. *Nurs Res*. 2001;50(5):275-285.

4. de Paula Eduardo JAF, de Rezende MG, Menezes PR, Del-Ben CM. Preterm birth as a risk factor for postpartum depression: A systematic review and meta-analysis. *J Affect Disord*. 2019;259:392-403. doi:10.1016/j.jad.2019.08.069

5. Blom EA, Jansen PW, Verhulst FC, et al. Perinatal complications increase the risk of postpartum depression. The Generation R Study. *BJOG Int J Obstet Gynaecol*. 2010;117(11):1390-1398. doi:10.1111/j.1471-0528.2010.02660.x

6. Beck CT. Postpartum depression: a metasynthesis. *Qual Health Res*. 2002;12(4):453-472. doi:10.1177/104973202129120016

7. Grace SL, Evindar A, Stewart DE. The effect of postpartum depression on child cognitive development and behavior: a review and critical analysis of the literature. *Arch Womens Ment Health*. 2003;6(4):263-274. doi:10.1007/s00737-003-0024-6

8. Lebel C, Walton M, Letourneau N, Giesbrecht GF, Kaplan BJ, Dewey D. Prepartum and postpartum maternal depressive symptoms are related to children's brain structure in preschool. *Biol Psychiatry*. 2016;80(11):859-868. doi:10.1016/j.biopsych.2015.12.004

9. Goodman SH, Rouse MH, Connell AM, Broth MR, Hall CM, Heyward D. Maternal depression and child psychopathology: a meta-analytic review. *Clin Child Fam Psychol Rev*. 2011;14(1):1-27. doi:10.1007/s10567-010-0080-1

10. Hagberg KW, Robijn AL, Jick S. Maternal depression and antidepressant use during pregnancy and the risk of autism spectrum disorder in offspring. *Clin Epidemiol*. 2018;10:1599-1612. doi:10.2147/CLEP.S180618

11. Stein A, Pearson RM, Goodman SH, et al. Effects of perinatal mental disorders on the fetus and child. *The Lancet*. 2014;384(9956):1800-1819. doi:10.1016/S0140-6736(14)61277-0

12. Van Batenburg-Eddes T, Brion MJ, Henrichs J, et al. Parental depressive and anxiety symptoms during pregnancy and attention problems in children: a cross-cohort consistency study. *J Child Psychol Psychiatry*. 2013;54(5):591-600. doi:10.1111/jcpp.12023

13. Barker ED, Jaffee SR, Uher R, Maughan B. The contribution of prenatal and postnatal maternal anxiety and depression to child maladjustment. *Depress Anxiety*. 2011;28(8):696-702. doi:10.1002/da.20856

14. Pearson RM, Evans J, Kounali D, et al. Maternal depression during pregnancy and the postnatal period: risks and possible mechanisms for offspring depression at 18 years. *JAMA Psychiatry*. 2013;70(12):1312-1319. doi:10.1001/jamapsychiatry.2013.2163

For peer review only

15. Kleine I, Falconer S, Roth S, et al. Early postnatal maternal trait anxiety is associated with the behavioural outcomes of children born preterm <33 weeks. *J Psychiatr Res*. 2020;131:160-168. doi:10.1016/j.jpsychires.2020.09.010

16. Feder A, Nestler EJ, Charney DS. Psychobiology and molecular genetics of resilience. *Nat Rev Neurosci*. 2009;10(6):446-457. doi:10.1038/nrn2649

17. World Health Organization. Preterm birth. Published February 19, 2018. Accessed May 18, 2022. https://www.who.int/news-room/fact-sheets/detail/preterm-birth

18. Dimitrova R, Pietsch M, Christiaens D, et al. Heterogeneity in brain microstructural development following preterm birth. *Cereb Cortex*. 2020;30(9):4800-4810. doi:10.1093/cercor/bhaa069

19. Fitzallen GC, Sagar YK, Taylor HG, Bora S. Anxiety and depressive disorders in children born preterm: a meta-analysis. *J Dev Behav Pediatr JDBP*. 2021;42(2):154-162. doi:10.1097/DBP.0000000000000898

20. Allotey J, Zamora J, Cheong-See F, et al. Cognitive, motor, behavioural and academic performances of children born preterm: a meta-analysis and systematic review involving 64 061 children. *BJOG Int J Obstet Gynaecol*. 2018;125(1):16-25. doi:10.1111/1471-0528.14832

21. Jaekel J, Pluess M, Belsky J, Wolke D. Effects of maternal sensitivity on low birth weight children's academic achievement: a test of differential susceptibility versus diathesis stress. *J Child Psychol Psychiatry*. 2015;56(6):693-701. doi:10.1111/jcpp.12331

22. Belsky J, Bakermans-Kranenburg MJ, van IJzendoorn MH. For better and for worse: differential susceptibility to environmental influences. *Curr Dir Psychol Sci*. 2007;16(6):300-304. doi:10.1111/j.1467-8721.2007.00525.x

23. Gueron-Sela N, Atzaba-Poria N, Meiri G, Marks K. The caregiving environment and developmental outcomes of preterm infants: diathesis stress or differential susceptibility effects? *Child Dev*. 2015;86(4):1014-1030. doi:https://doi.org/10.1111/cdev.12359

24. Hadfield K, O'Brien F, Gerow A. Is level of prematurity a risk/plasticity factor at three years of age? *Infant Behav Dev*. 2017;47:27-39. doi:10.1016/j.infbeh.2017.03.003

25. Belsky J, Pluess M. Beyond diathesis stress: differential susceptibility to environmental influences. *Psychol Bull*. 2009;135(6):885-908. doi:10.1037/a0017376

26. Pluess M, Belsky J. Prenatal programming of postnatal plasticity? *Dev Psychopathol*. 2011;23(1):29-38. doi:10.1017/S0954579410000623

27. DeMaster D, Bick J, Johnson U, Montroy JJ, Landry S, Duncan AF. Nurturing the preterm infant brain: leveraging neuroplasticity to improve neurobehavioral outcomes. *Pediatr Res*. 2019;85(2):166-175. doi:10.1038/s41390-018-0203-9

28. World Health Organization (WHO). Global nutrition targets 2025: low birth weight policy brief. Published online 2014.

29. Kramer MS. Determinants of low birth weight: methodological assessment and meta-analysis. *Bull World Health Organ*. 1987;65(5):663-737.

30. Goldenberg RL, Culhane JF, Iams JD, Romero R. Epidemiology and causes of preterm birth. *Lancet Lond Engl*. 2008;371(9606):75-84. doi:10.1016/S0140-6736(08)60074-4

For peer review only

For peer review only - http://bmjopen.bmj.com/site/about/guidelines.xhtml

31. Carson C, Redshaw M, Gray R, Quigley MA. Risk of psychological distress in parents of preterm children in the first year: evidence from the UK Millennium Cohort Study. *BMJ Open*. 2015;5(12):e007942. doi:10.1136/bmjopen-2015-007942

32. Vigod SN, Villegas L, Dennis CL, Ross LE. Prevalence and risk factors for postpartum depression among women with preterm and low-birth-weight infants: a systematic review. *BJOG Int J Obstet Gynaecol*. 2010;117(5):540-550. doi:10.1111/j.1471-0528.2009.02493.x

33. Johnson S, Wolke D. Behavioural outcomes and psychopathology during adolescence. *Early Hum Dev*. 2013;89(4):199-207. doi:10.1016/j.earlhumdev.2013.01.014

34. Lautarescu A, Victor S, Lau-Zhu A, Counsell SJ, Edwards AD, Craig MC. The factor structure of the Edinburgh Postnatal Depression Scale among perinatal high-risk and community samples in London. *Arch Womens Ment Health*. 2022;25(1):157-169. doi:10.1007/s00737-021-01153-0

35. Ministry of Housing, Communities & Local Government. The English indices of deprivation 2019. Published online 2019. https://assets.publishing.service.gov.uk/government/uploads/system/uploads/attachment_data/file/835115/IoD2019_Statistical_Release.pdf

36. Boyd A, Golding J, Macleod J, et al. Cohort Profile: The ‘Children of the 90s’—the index offspring of the Avon Longitudinal Study of Parents and Children. *Int J Epidemiol*. 2013;42(1):111-127. doi:10.1093/ije/dys064

37. Cox JL, Holden JM, Sagovsky R. Detection of postnatal depression: development of the 10-item Edinburgh Postnatal Depression Scale. *Br J Psychiatry*. 1987;150(6):782-786. doi:10.1192/bjp.150.6.782

38. Levis B, Negeri Z, Sun Y, Benedetti A, Thombs BD. Accuracy of the Edinburgh Postnatal Depression Scale (EPDS) for screening to detect major depression among pregnant and postpartum women: systematic review and meta-analysis of individual participant data. *BMJ*. 2020;371. doi:10.1136/bmj.m4022

39. Achenbach TM, Rescorla LA. *Manual for the ASEBA Preschool Forms & Profiles*.; 2000.

40. Allison C, Baron-Cohen S, Wheelwright S, et al. The Q-CHAT (Quantitative Checklist for Autism in Toddlers): a normally distributed quantitative measure of autistic traits at 18-24 months of age: preliminary report. *J Autism Dev Disord*. 2008;38(8):1414-1425. doi:10.1007/s10803-007-0509-7

41. Allison C, Matthews FE, Ruta L, et al. Quantitative Checklist for Autism in Toddlers (Q-CHAT). A population screening study with follow-up: the case for multiple time-point screening for autism. *BMJ Paediatr Open*. 2021;5(1):e000700. doi:10.1136/bmjpo-2020-000700

42. Bayley N. *Bayley Scales of Infant and Toddler Development - Third Edition*. 3rd ed. Harcourt Assessment; 2006.

43. Albers CA, Grieve AJ. Review of Bayley scales of infant and toddler development-third edition. *J Psychoeduc Assess*. 2007;25(2):180-190. doi:10.1177/0734282906297199

44. Anderson PJ, De Luca CR, Hutchinson E, Roberts G, Doyle LW, the Victorian Infant Collaborative Group. Underestimation of developmental delay by the new Bayley-III scale. *Arch Pediatr Adolesc Med*. 2010;164(4):352-356. doi:10.1001/archpediatrics.2010.20

45. Rubin DB. Inference and missing data. *Biometrika*. 1976;63(3):581-592. doi:10.2307/2335739

For peer review only

For peer review only - http://bmjopen.bmj.com/site/about/guidelines.xhtml

46. Cohen J. *Statistical Power Analysis for the Behavioral Sciences*. 2nd ed. L. Erlbaum Associates; 1988.

47. Selya AS, Rose JS, Dierker LC, Hedeker D, Mermelstein RJ. A practical guide to calculating Cohen's f2, a measure of local effect size, from PROC MIXED. *Front Psychol*. 2012;3. doi:10.3389/fpsyg.2012.00111

48. Harel O. The estimation of R 2 and adjusted R 2 in incomplete data sets using multiple imputation. *J Appl Stat*. 2009;36(10):1109-1118. doi:10.1080/02664760802553000

49. Plomin R, Haworth CMA, Davis OSP. Common disorders are quantitative traits. *Nat Rev Genet*. 2009;10(12):872-878. doi:10.1038/nrg2670

50. Conroy S, Pariante CM, Marks MN, et al. Maternal psychopathology and infant development at 18 months: the impact of maternal personality disorder and depression. *J Am Acad Child Adolesc Psychiatry*. 2012;51(1):51-61. doi:10.1016/j.jaac.2011.10.007

51. Kingston D, Kehler H, Austin MP, et al. Trajectories of maternal depressive symptoms during pregnancy and the first 12 months postpartum and child externalizing and internalizing behavior at three years. *PloS One*. 2018;13(4):e0195365. doi:10.1371/journal.pone.0195365

52. Wiggins LD, Rubenstein E, Daniels J, et al. A phenotype of childhood autism is associated with preexisting maternal anxiety and depression. *J Abnorm Child Psychol*. 2019;47(4):731-740. doi:10.1007/s10802-018-0469-8

53. Babb JA, Deligiannidis KM, Murgatroyd CA, Nephew BC. Peripartum depression and anxiety as an integrative cross-domain target for psychiatric preventative measures. *Behav Brain Res*. 2015;0:32-44. doi:10.1016/j.bbr.2014.03.039

54. Pohl AL, Crockford SK, Blakemore M, Allison C, Baron-Cohen S. A comparative study of autistic and non-autistic women's experience of motherhood. *Mol Autism*. 2020;11(1):3. doi:10.1186/s13229-019-0304-2

55. Colvert E, Tick B, McEwen F, et al. Heritability of autism spectrum disorder in a UK population-based twin sample. *JAMA Psychiatry*. 2015;72(5):415-423. doi:10.1001/jamapsychiatry.2014.3028

56. Egger HL, Emde RN. Developmentally sensitive diagnostic criteria for mental health disorders in early childhood: the diagnostic and statistical manual of mental disorders-IV, the research diagnostic criteria-preschool age, and the diagnostic classification of mental health and developmental disorders of infancy and early childhood-revised. *Am Psychol*. 2011;66(2):95-106. doi:10.1037/a0021026

57. Henshaw C. Mood disturbance in the early puerperium: a review. *Arch Women's Ment Health*. 2003;6(2):s33-s42. doi:10.1007/s00737-003-0004-x

58. Heron J, O'Connor TG, Evans J, Golding J, Glover V. The course of anxiety and depression through pregnancy and the postpartum in a community sample. *J Affect Disord*. 2004;80(1):65-73. doi:10.1016/j.jad.2003.08.004

59. Biel MG. Parental psychiatric symptoms and children's outcomes: toward understanding and responding to intergenerational risk in child psychiatry. *J Am Acad Child Adolesc Psychiatry*. 2018;57(9):632-633. doi:10.1016/j.jaac.2018.06.010

For peer review only - http://bmjopen.bmj.com/site/about/guidelines.xhtml

60. National Institute for Health and Care Excellence. Preterm labour and birth (NICE guideline 25). Published online November 2015. Accessed January 27, 2019. https://www.nice.org.uk/guidance/ng25

61. Office for National Statistics. Birth characteristics in England and Wales: 2020. Published January 13, 2022. Accessed February 5, 2022. https://www.ons.gov.uk/peoplepopulationandcommunity/birthsdeathsandmarriages/livebirths/bulletins/birthcharacteristicsinenglandandwales/2020#birth-characteristics

62. Londero AP, Rossetti E, Pittini C, Cagnacci A, Driul L. Maternal age and the risk of adverse pregnancy outcomes: a retrospective cohort study. *BMC Pregnancy Childbirth*. 2019;19(1):261. doi:10.1186/s12884-019-2400-x

63. Goh DA, Gan D, Kung J, et al. Child, maternal and demographic factors influencing caregiver-reported autistic trait symptomatology in toddlers. *J Autism Dev Disord*. 2018;48(4):1325-1337. doi:10.1007/s10803-018-3471-7

64. Friedlander S, Weiss DS, Traylor J. Assessing the influence of maternal depression on the validity of the Child Behavior Checklist. *J Abnorm Child Psychol*. 1986;14(1):123-133. doi:10.1007/bf00917228

For peer review only

Table 1: Socio-demographic, maternal and clinical characteristics (n=509)

**Variable**	**Number (%)***
Sex: Male	274 (53.8)
Index of Multiple Deprivation (IMD) quintiles	
1 (least deprived) [a]	65 (12.8)
2	87 (17.2)
3	108 (21.3)
4	173 (34.2)
5 (most deprived)	73 (14.4)
Gestational age at birth (weeks), median [range]	39.7 [20 – 43]
Gestational category	
Extremely preterm (<28 weeks)	18 (3.5)
Very preterm (28-32 weeks)	28 (5.5)
Late preterm (32-37 weeks)	51 (10.0)
Term (≥37 weeks)	412 (80.9)
Birthweight (g), median [range]	3290 [450 – 4750]
Multiple pregnancy	54 (10.6)
Maternal parity	
0	332 (65.2)
1	124 (24.4)
2	32 (6.3)
3+	21 (4.2)
Maternal BMI (kg/m^2), median [range]	23.2 [15.3 – 43.6]
Maternal age at infant's birth (years), mean (SD) [range]	34.2 (4.8) [17 – 52]
Maternal ethnicity	
White	272 (53.4)
Black/Black British	56 (11.0)
Asian/Asian British	28 (5.5)
Chinese	18 (3.5)
Mixed – White & Asian	4 (0.8)
Mixed – White & Black	4 (0.8)
Any other	30 (5.9)
Do not wish to answer	9 (1.8)
No data	88 (17.3)
Bayley III cognitive composite score, mean (SD) [range]	100 (11.4) [55 – 125]
CBCL total T score, mean (SD) [range]	46.9 (9.5) [28 – 69]
Q-CHAT total score, mean (SD) [range]	30.5 (9.3) [8 – 70]
EPDS score, median [range]	4 [0 – 28]
EPDS score, n (%)	
<13	415 (8.2)
≥13	21 (4.1)
No data	73 (14.3)

[a] Quintile 1 corresponds to the highest, least deprived, IMD rankings.

* unless otherwise specified

For peer review only - http://bmjopen.bmj.com/site/about/guidelines.xhtml

Table 2: CBCL and Q-CHAT model predictors using multiple imputation without interaction. (Cf. Supplementary Table 3 for complete case analysis)

	CBCL			Q-CHAT		
	**B [95%CI]**	**p**	**f^2**	**B [95%CI]**	**p**	**f^2**
**Maternal EPDS**	0.93 [0.43, 1.44]	<.001 ***	0.05	0.27 [0.03, 0.52]	.031 *	0.01
**Maternal BMI**	-0.09 [-0.44, 0.26]	.621	-	0.06 [-0.13, 0.24]	.538	-
**Multiple pregnancy**	3.15 [-3.07, 9.37]	.320	-	1.33 [-2.62, 5.28]	.509	-
**Parity**						
**1**	-2.52 [-5.96, 0.93]	.151	-	-2.14 [-4.02, -0.27]	.025 [a]	-
**2**	-3.23 [-9.16, 2.70]	.285	-	0.88 [-1.99, 3.75]	.548	-
**3+**	-1.37 [-8.36, 5.61]	.699	-	-0.49 [-4.57, 3.60]	.815	-
**IMD rank**	-1.48 [-3.33, 0.37]	.117	-	-1.50 [-2.60, -0.40]	.008 **	0.02
**Gestational age at birth (weeks)**	0.10 [-0.65, 0.85]	.786	-	0.26 [-0.17, 0.70]	.233	-
**Birthweight (kg)**	0.56 [-2.65, 3.78]	.731	-	-1.24 [-2.93, 0.46]	.151	-
**Sex:female**	-4.14 [-6.96, -1.31]	.004 **	0.06	-1.95 [-3.42, -0.48]	.009 **	0.05
**Corrected age at assessment (months)**	-0.90 [-2.17, 0.37]	.166	-	-0.16 [-0.91, 0.59]	.677	-
**Cognition**	-0.05 [-0.20, 0.09]	.467	-	-0.27 [-0.35, -0.20]	<.001 ***	0.12

p<0.05 *; p<0.01 **; p<0.001 ***
CBCL model adjusted R^2 = 0.0676. Q-CHAT model adjusted R^2 = 0.193.
B = unstandardised coefficient.
CBCL = Child Behaviour Checklist score at 18 months. Q-CHAT = Quantitative Checklist for Autism in Toddlers score at 18 months. Maternal EPDS = maternal Edinburgh Postnatal Depression Scale score at term-equivalent age. Multiple pregnancy = dummy variable of twin/triplet pregnancy. Parity = dummy variable, one/two/three+ previous child(ren). Corrected age at assessment (months) = age at behavioural assessment, corrected for gestational age. Cognition = infant Bayley III score at 18 months.
[a] Wald test of whole parity variable in Q-CHAT model: F(3, 476.9) = 1.88, p = .133
Effect size (Cohen's f^2, calculated from squared part correlations for predictors significant to 0.05): 0.02 = small, 0.15 = medium and 0.35 = large.[46]
- indicates data not given, as predictor not significant to 0.05.

For peer review only - http://bmjopen.bmj.com/site/about/guidelines.xhtml

Table 3: CBCL and Q-CHAT model predictors using complete case analysis with interaction of 'EPDS x term'.

	CBCL		Q-CHAT	
	**B [95%CI]**	**p**	**B [95%CI]**	**p**
**Maternal EPDS**	0.89 [-0.24, 2.02]	.121	0.24 [-0.28, 0.75]	.365
**Maternal BMI**	-0.01 [-0.39, 0.37]	.955	0.00 [-0.16, 0.17]	.982
**Multiple pregnancy**	1.76 [-6.65, 10.17]	.681	0.97 [-2.07, 4.01]	.532
**Parity**				
**1**	-2.75 [-6.49, 0.99]	.149	-1.42 [-3.30, 0.46]	.139
**2**	-3.49 [-10.36, 3.37]	.317	0.16 [-2.84, 3.16]	.917
**3+**	-1.17 [-9.69, 7.35]	.788	-1.13 [-4.24, 1.98]	.476
**IMD rank**	-1.41 [-3.54, 0.73]	.195	-1.68 [-2.64, -0.72]	.001 **
**Gestation: term**	1.25 [-8.34, 10.85]	.797	2.64 [-1.74, 7.02]	.236
**Birthweight (kg)**	-1.01 [-4.08, 2.05]	.516	-2.25 [-3.73, -0.78]	.003 **
**Sex: female**	-4.64 [-7.83, -1.44]	.005 **	-2.22 [-3.72, -0.71]	.004 **
**Corrected age at assessment (months)**	-0.83 [-2.27, 0.62]	.261	-0.39 [-1.18, 0.04]	.335
**Cognition**	-0.03 [-0.20, 0.14]	.720	-0.22 [-0.29, -.0.15]	<.001 ***
**EPDS x gestation:term**	-0.01 [-1.30, 1.28]	.991	-0.02 [-0.60, 0.56]	.950

$p<0.05$ *; $p<0.01$ **; $p<0.001$ ***
CBCL model adjusted $R^2 = 0.0865$. Q-CHAT model adjusted $R^2 = 0.215$.
B = unstandardised coefficient.
CBCL = Child Behaviour Checklist score at 18 months. Q-CHAT = Quantitative Checklist for Autism in Toddlers score at 18 months. Maternal EPDS = maternal Edinburgh Postnatal Depression Scale score at term-equivalent age. Multiple pregnancy = dummy variable of twin/triplet pregnancy. Parity = dummy variable, one/two/three+ previous child(ren). Gestation: term = dummy variable, term (≥37 weeks) vs preterm (<37 weeks) gestation at birth. Corrected age at assessment (months) = age at behavioural assessment, corrected for gestational age. Cognition = infant Bayley III score at 18 months. EPDS x gestation:term = interaction term between maternal EPDS score and term gestation at birth.

For peer review only - http://bmjopen.bmj.com/site/about/guidelines.xhtml

Fig.1 Children's predicted CBCL scores at 18 months are positively correlated to the maternal EPDS score at term-equivalent age.

Fig.2 Children's predicted Q-CHAT scores at 18 months are positively correlated to the maternal EPDS score at term-equivalent age.

For peer review only

For peer review only - http://bmjopen.bmj.com/site/about/guidelines.xhtml

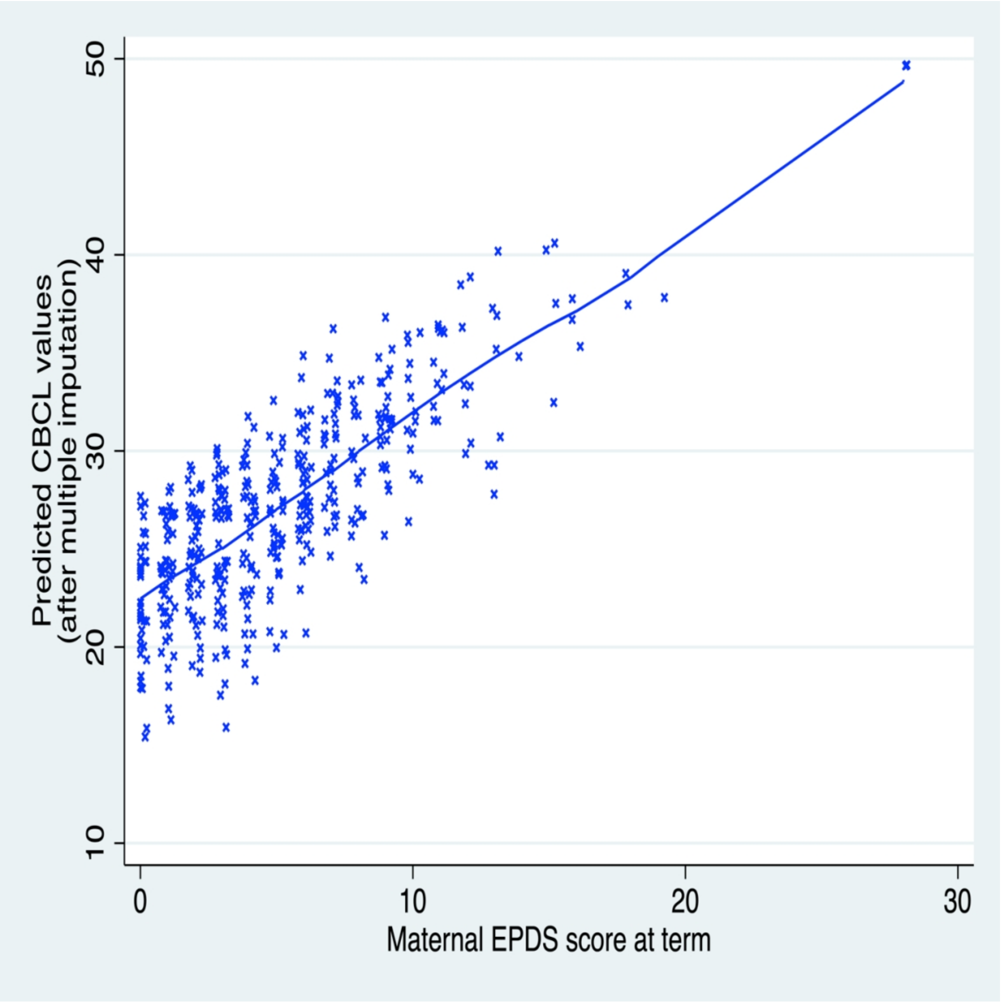

Fig.1 Children's predicted CBCL scores at 18 months are positively correlated to the maternal EPDS score at term-equivalent age

158x158mm (220 x 220 DPI)

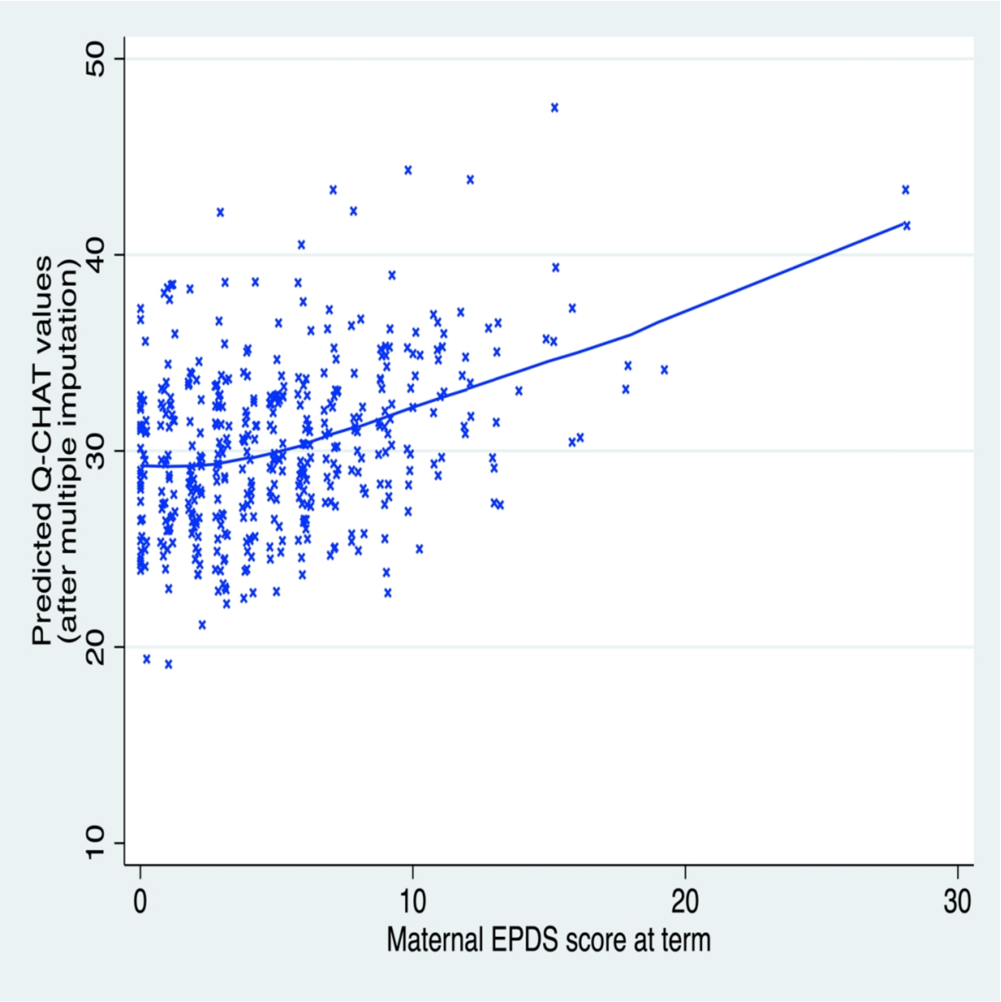

Fig.2 Children’s predicted Q-CHAT scores at 18 months are positively correlated to the maternal EPDS score at term-equivalent age.

158x158mm (220 x 220 DPI)

Supplemental material

Supplementary Table 1: CBCL internalising symptom model predictors using multiple imputation.

Supplementary Table 2: CBCL externalising symptom model predictors using multiple imputation.

Supplementary Table 3: CBCL and Q-CHAT model predictors using complete case analysis without interaction.

Supplementary Table 4: EPDS score predictors.

Supplementary Table 5: EPDS score predictors including interaction 'term x time-lag'.

Supplementary Figure 1: Histogram showing the distribution of maternal EPDS scores at term-equivalent age.

For peer review only

Supplementary Table 1: CBCL internalising symptom model predictors using multiple imputation**.**

	B [95%CI]	p	f^2
**Maternal EPDS**	0.22 [0.08, 0.36]	.003 **	0.03
**Maternal BMI**	-0.04 [-0.13, 0.06]	.436	-
**Multiple pregnancy**	0.58 [-1.10, 2.27]	.497	-
**Parity**			
**1**	-0.37 [-1.41, 0.67]	.487	-
**2**	-1.33 [-3.09, 0.42]	.136	-
**3+**	0.64 [-1.34, 2.62]	.524	-
**IMD rank**	-0.41 [-0.91, 0.10]	.115	-
**Gestational age at birth (weeks)**	0.22 [-0.00, 0.44]	.053	-
**Birthweight (kg)**	-0.97 [-1.90, -0.05]	.038 *	0.005
**Sex: female**	-0.51 [-1.35, 0.33]	.232	-
**Corrected age at assessment (months)**	0.02 [-0.41, 0.45]	.923	-
**Cognition**	-0.05 [-0.10, -0.01]	.016 *	0.01

p<0.05 *; p<0.01 **; p<0.001 ***
Adjusted R^2 = 0.0566.
B = unstandardised coefficient.
Outcome variable = Child Behaviour Checklist internalising sub-score at 18 months. Maternal EPDS = maternal Edinburgh Postnatal Depression Scale score at term-equivalent age. Multiple pregnancy = dummy variable of twin/triplet pregnancy. Parity = dummy variable, one/two/three+ previous child(ren). Corrected age at assessment (months) = age at behavioural assessment, corrected for gestational age. Cognition = infant Bayley III score at 18 months.
Effect size (Cohen's f^2, calculated from squared part correlations for predictors significant to 0.05): 0.02 = small, 0.15 = medium and 0.35 = large.[1]
- indicates data not given, as predictor not significant to 0.05.

For peer review only

Supplementary Table 2: CBCL externalising symptom model predictors using multiple imputation.

	**B [95%CI]**	**p**	**f^2**
**Maternal EPDS**	0.40 [0.20, 0.61]	<.001 ***	0.05
**Maternal BMI**	0.01 [-0.17, 0.15]	.933	-
**Multiple pregnancy**	2.51 [-0.29, 5.31]	.079	-
**Parity**			
**1**	-1.06 [-2.53, 0.42]	.160	-
**2**	-0.61 [-3.55, 2.33]	.682	-
**3+**	-0.96 [-4.47, 2.56]	.593	-
**IMD rank**	-0.24 [-1.11, 0.63]	.585	-
**Gestational age at birth (weeks)**	-0.07 [-0.40, 0.27]	.701	-
**Birthweight (kg)**	1.03 [-0.38, 2.44]	.153	-
**Sex: female**	-1.80 [-3.07, -0.53]	.006 **	0.06
**Corrected age at assessment (months)**	-0.40 [-0.95, 0.16]	.161	-
**Cognition**	0.03 [-0.03, 0.10]	.322	-

p<0.05 *; p<0.01 **; p<0.001 ***
Adjusted R^2 = 0.0612.
B = unstandardised coefficient.
Outcome variable = Child Behaviour Checklist externalising sub-score at 18 months. Maternal EPDS = maternal Edinburgh Postnatal Depression Scale score at term-equivalent age. Multiple pregnancy = dummy variable of twin/triplet pregnancy. Parity = dummy variable, one/two/three+ previous child(ren). Corrected age at assessment (months) = age at behavioural assessment, corrected for gestational age. Cognition = infant Bayley III score at 18 months.
Effect size (Cohen's f^2, calculated from squared part correlations for predictors significant to 0.05): 0.02 = small, 0.15 = medium and 0.35 = large.[1]
- indicates data not given, as predictor not significant to 0.05.

For peer review only

Supplementary Table 3: CBCL and Q-CHAT model predictors using complete case analysis without interaction.

	CBCL		Q-CHAT	
	**B [95%CI]**	**p**	**B [95%CI]**	**p**
**Maternal EPDS**	0.88 [0.35, 1.41]	.001 **	0.21 [-0.02, 0.44]	.069
**Maternal BMI**	-0.01 [-0.38, 0.37]	.963	0.01 [-0.16, 0.17]	.930
**Multiple pregnancy**	1.50 [-6.87, 9.87]	.724	0.43 [-2.50, 3.37]	.772
**Parity**				
**1**	-2.83 [-6.60, 0.94]	.141	-1.54 [-3.41, 0.34]	.108
**2**	-3.49 [-10.3, 3.35]	.316	0.13 [-2.85, 3.10]	.933
**3+**	-1.38 [-10.0, 7.30]	.755	-1.43 [-4.50, 1.64]	.360
**IMD rank**	-1.44 [-3.56, 0.67]	.181	-1.75 [-2.70, -0.79]	<.001 ***
**Gestational age at birth (weeks)**	0.01 [-0.90, 0.91]	.987	0.10 [-0.33, 0.54]	.639
**Birthweight (kg)**	-0.65 [-4.38, 3.08]	.733	-1.81 [-3.63, 0.00]	.050
**Sex: female**	-4.57 [-7.82, -1.31]	.006 **	-2.12 [-3.60, -0.64]	.005 **
**Corrected age at assessment (months)**	-0.84 [-2.26, 0.59]	.247	-0.41 [-1.18, 0.35]	.290
**Cognition**	-0.03 [-0.20, 0.13]	.689	-0.23 [-0.30, -0.15]	<.001 ***

$p<0.05$ *; $p<0.01$ **; $p<0.001$ ***
CBCL adjusted $R^2 = 0.0862$. Q-CHAT adjusted $R^2 = 0.2103$.
B = unstandardised coefficient.
CBCL = Child Behaviour Checklist externalising sub-score at 18 months. Q-CHAT = Quantitative Checklist for Autism in Toddlers score at 18 months. Maternal EPDS = maternal Edinburgh Postnatal Depression Scale score at term-equivalent age. Multiple pregnancy = dummy variable of twin/triplet pregnancy. Parity = dummy variable, one/two/three+ previous child(ren). Gestation (weeks) = dummy variable: 34-36+6 weeks and ≥37 weeks gestation at birth. Corrected age at assessment (months) = age at behavioural assessment, corrected for gestational age. Cognition = infant Bayley III score at 18 months.

For peer review only

Supplementary Table 4: EPDS score predictors.

	IRR [95%CI]	p
**Time-lag (weeks)**	1.01 [0.97, 1.05]	.647
**Gestation:term**	0.91 [0.64, 1.31]	.627
**IMD rank**	1.00 [1.00, 1.00]	.103
**Multiple pregnancy**	0.66 [0.46, 0.96]	.031 *
**Parity**		
**1**	0.79 [0.66, 0.95]	.011 *
**2**	0.87 [0.60, 1.28]	.491
**3+**	0.84 [0.54, 1.31]	.445
**Birthweight (kg)**	0.98 [0.83, 1.17]	.847
**Sex:female**	1.13 [0.98, 1.31]	.098

$p<0.05$ *; $p<0.01$ **; $p<0.001$ ***
Pseudo $R^2 = 0.0228$
IRR = incidence rate ratio
Outcome variable = maternal Edinburgh Postnatal Depression Scale (EPDS) score at term-equivalent age. Time-lag (weeks) = time in weeks between birth and EPDS assessment. Gestation:term = dummy variable, term (≥37 weeks) vs preterm (<37 weeks) gestation at birth. Multiple pregnancy = dummy variable of twin/triplet pregnancy. Parity = dummy variable, one/two/three+ previous child(ren).

For peer review only

Supplementary Table 5: EPDS score predictors including interaction 'term x time-lag'.

	IRR [95%CI]	p
**Time-lag (weeks)**	1.00 [0.97, 1.04]	.823
**Gestation: term**	0.88 [0.58, 1.34]	.553
**IMD rank**	1.00 [1.00, 1.00]	.104
**Multiple pregnancy**	0.66 [0.46, 0.96]	.029 *
**Parity**		
**1**	0.79 [0.66, 0.95]	.010 *
**2**	0.87 [0.60, 1.28]	.480
**3+**	0.85 [0.55, 1.31]	.458
**Birthweight (kg)**	0.97 [0.83, 1.15]	.756
**Sex:female**	1.13 [0.98, 1.30]	.100
**Term x time-lag (weeks)**	1.01 [0.94, 1.10]	.735

p<0.05 *; p<0.01 **; p<0.001 ***
Pseudo $R^2 = 0.0230$
IRR = incidence rate ratio
Outcome variable = maternal Edinburgh Postnatal Depression Scale (EPDS) score at term-equivalent age. Time-lag (weeks) = time in weeks between birth and EPDS assessment. Gestation: term = dummy variable, term (≥37 weeks) vs preterm (<37 weeks) gestation at birth. Multiple pregnancy = dummy variable of twin/triplet pregnancy. Parity = dummy variable, one/two/three+ previous child(ren). Term x time-lag (weeks): interaction term between term gestation at birth and time-lag between birth and maternal EPDS assessment.

For peer review only

Supplementary Figure 1: Histogram showing the distribution of maternal EPDS scores at term-equivalent age.

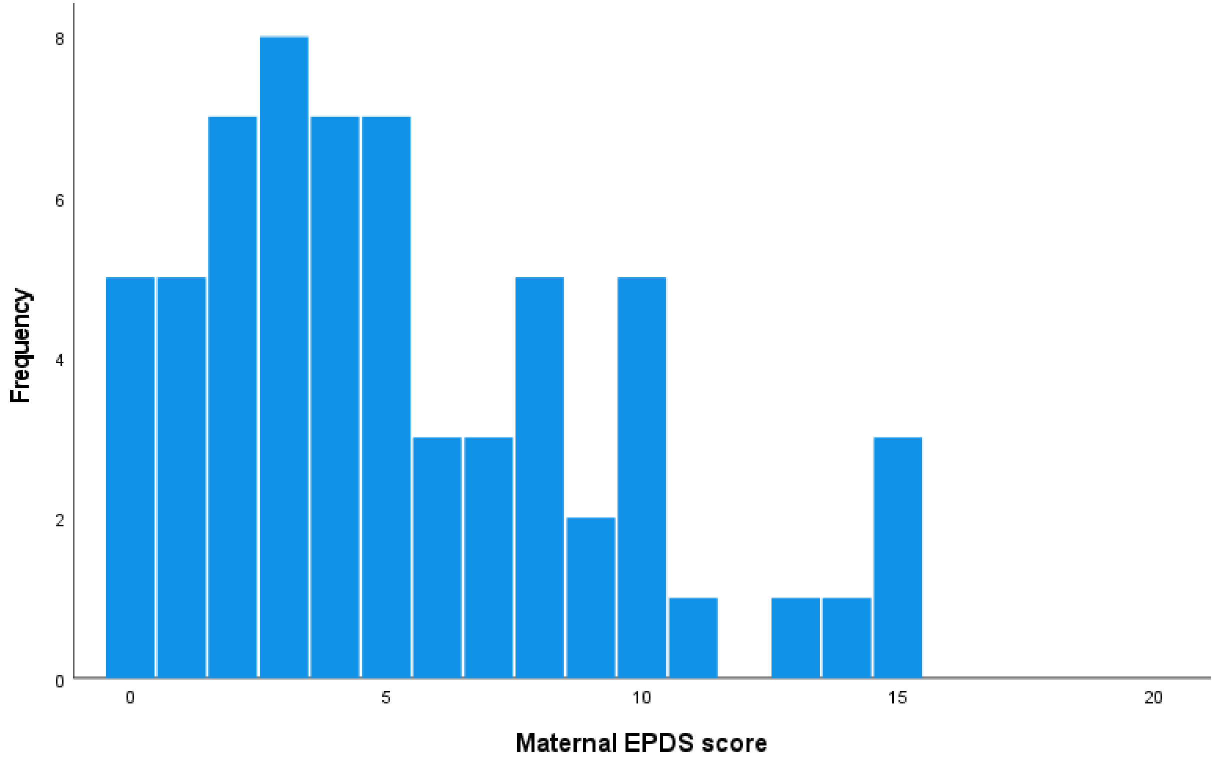

Supplemental reference list

1. Cohen J. *Statistical Power Analysis for the Behavioral Sciences*. 2nd ed. L. Erlbaum Associates; 1988.

For peer review only

STROBE Statement—Checklist of items that should be included in reports of ***cohort studies***

	Item No	Recommendation	Page No
**Title and abstract**	1	(*a*) Indicate the study's design with a commonly used term in the title or the abstract<br>(*b*) Provide in the abstract an informative and balanced summary of what was done and what was found	1, 2
**Introduction**			
Background/rationale	2	Explain the scientific background and rationale for the investigation being reported	5, 6
Objectives	3	State specific objectives, including any prespecified hypotheses	6
**Methods**			
Study design	4	Present key elements of study design early in the paper	6, 7
Setting	5	Describe the setting, locations, and relevant dates, including periods of recruitment, exposure, follow-up, and data collection	7
Participants	6	(*a*) Give the eligibility criteria, and the sources and methods of selection of participants. Describe methods of follow-up<br>(*b*) For matched studies, give matching criteria and number of exposed and unexposed	7
Variables	7	Clearly define all outcomes, exposures, predictors, potential confounders, and effect modifiers. Give diagnostic criteria, if applicable	7, 8
Data sources/ measurement	8*	For each variable of interest, give sources of data and details of methods of assessment (measurement). Describe comparability of assessment methods if there is more than one group	7, 8
Bias	9	Describe any efforts to address potential sources of bias	9
Study size	10	Explain how the study size was arrived at	7
Quantitative variables	11	Explain how quantitative variables were handled in the analyses. If applicable, describe which groupings were chosen and why	9, 10
Statistical methods	12	(*a*) Describe all statistical methods, including those used to control for confounding<br>(*b*) Describe any methods used to examine subgroups and interactions<br>(*c*) Explain how missing data were addressed<br>(*d*) If applicable, explain how loss to follow-up was addressed<br>(*e*) Describe any sensitivity analyses	9, 10, 11
**Results**			
Participants	13*	(a) Report numbers of individuals at each stage of study—eg numbers potentially eligible, examined for eligibility, confirmed eligible, included in the study, completing follow-up, and analysed<br>(b) Give reasons for non-participation at each stage<br>(c) Consider use of a flow diagram	7, 11
Descriptive data	14*	(a) Give characteristics of study participants (eg demographic, clinical, social) and information on exposures and potential confounders<br>(b) Indicate number of participants with missing data for each variable of interest<br>(c) Summarise follow-up time (eg, average and total amount)	9, 11
Outcome data	15*	Report numbers of outcome events or summary measures over time	11

For peer review only

Main results	16	(*a*) Give unadjusted estimates and, if applicable, confounder-adjusted estimates and their precision (eg, 95% confidence interval). Make clear which confounders were adjusted for and why they were included<br>(*b*) Report category boundaries when continuous variables were categorized<br>(*c*) If relevant, consider translating estimates of relative risk into absolute risk for a meaningful time period	11, 12
Other analyses	17	Report other analyses done—eg analyses of subgroups and interactions, and sensitivity analyses	11, 12
**Discussion**			
Key results	18	Summarise key results with reference to study objectives	12
Limitations	19	Discuss limitations of the study, taking into account sources of potential bias or imprecision. Discuss both direction and magnitude of any potential bias	15, 16, 17
Interpretation	20	Give a cautious overall interpretation of results considering objectives, limitations, multiplicity of analyses, results from similar studies, and other relevant evidence	13, 14, 17
Generalisability	21	Discuss the generalisability (external validity) of the study results	13, 14
**Other information**			
Funding	22	Give the source of funding and the role of the funders for the present study and, if applicable, for the original study on which the present article is based	3

*Give information separately for exposed and unexposed groups.

Note: An Explanation and Elaboration article discusses each checklist item and gives methodological background and published examples of transparent reporting. The STROBE checklist is best used in conjunction with this article (freely available on the Web sites of PLoS Medicine at http://www.plosmedicine.org/, Annals of Internal Medicine at http://www.annals.org/, and Epidemiology at http://www.epidem.com/). Information on the STROBE Initiative is available at http://www.strobe-statement.org.

For peer review only
